# The World Mental Health International College Student Survey in Canada: Protocol for a Mental Health and Substance Use Trend Study

**DOI:** 10.2196/35168

**Published:** 2022-07-29

**Authors:** Laura B Jones, Carolina Judkowicz, Kristen L Hudec, Richard J Munthali, Ana Paula Prescivalli, Angel Y Wang, Lonna Munro, Hui Xie, Krishna Pendakur, Brian Rush, James Gillett, Marisa Young, Diana Singh, Antoaneta A Todorova, Randy P Auerbach, Ronny Bruffaerts, Sarah M Gildea, Irene McKechnie, Anne Gadermann, Chris G Richardson, Nancy A Sampson, Ronald C Kessler, Daniel V Vigo

**Affiliations:** 1 Department of Psychiatry Faculty of Medicine University of British Columbia Vancouver, BC Canada; 2 Faculty of Health Sciences Simon Fraser University Burnaby, BC Canada; 3 Department of Economics Simon Fraser University Burnaby, BC Canada; 4 Social & Behavioural Health Sciences Division Dalla Lana School of Public Health University of Toronto Toronto, ON Canada; 5 Department of Psychiatry University of Toronto Toronto, ON Canada; 6 Department of Health, Aging & Society Faculty of Social Sciences McMaster University Hamilton, ON Canada; 7 Department of Sociology Faculty of Social Sciences McMaster University Hamilton, ON Canada; 8 Dalla Lana School of Public Health University of Toronto Toronto, ON Canada; 9 Department of Psychiatry Columbia University New York, NY United States; 10 Center for Public Health Psychiatry Katholieke Universiteit Leuven Leuven Belgium; 11 Department of Health Care Policy Harvard Medical School Boston, MA United States; 12 Okanagan Planning and Institutional Research University of British Columbia Kelowna, BC Canada; 13 School of Population and Public Health Faculty of Medicine University of British Columbia Vancouver, BC Canada

**Keywords:** mental health, substance use, student health, World Mental Health International College Student Initiative, Canada, Canadian, online survey, survey, questionnaire, screen, epidemiology, depression, anxiety, trend

## Abstract

**Background:**

The World Health Organization World Mental Health International College Student (WMH-ICS) initiative aims to screen for mental health and substance use problems among postsecondary students on a global scale as well as to develop and evaluate evidence-based preventive and ameliorative interventions for this population. This protocol paper presents the Canadian version of the WMH-ICS survey, detailing the adapted survey instrument, the unique weekly cross-sectional administration, the multitiered recruitment strategy, and the associated risk mitigation protocols.

**Objective:**

This paper aims to provide a methodological resource for researchers conducting cross-national comparisons of WMH-ICS data, as well as to serve as a useful guide for those interested in replicating the outlined cross-sectional methodology to better understand how mental health and substance use vary over time among university students.

**Methods:**

The online survey is based on the WMH-ICS survey instrument, modified to the Canadian context by the addition of questions pertaining to Canadian-based guidelines and the translation of the survey to Canadian French. The survey is administered through the Qualtrics survey platform and is sent to an independent stratified random sample of 350 students per site weekly, followed by two reminder emails. Upon survey closure every week, a random subsample of 70 nonresponders are followed up with via phone or through a personal email in an effort to decrease nonresponder bias. The survey is accompanied by an extensive risk mitigation protocol that stratifies respondents by the level of need and provides tailored service recommendations, including a facilitated expedited appointment to student counseling services for those at increased risk of suicide. The anticipated sample size is approximately 5500 students per site per year.

**Results:**

In February 2020, the Canadian survey was deployed at the University of British Columbia. This was followed by deployment at Simon Fraser University (November 2020), McMaster University (January 2021), and the University of Toronto (January 2022). Data collection at all 4 sites is ongoing. As of May 6, 2022, 29,503 responses have been collected.

**Conclusions:**

Based on international collaboration, the Canadian version of the WMH-ICS survey incorporates a novel methodological approach centered on the weekly administration of a comprehensive cross-sectional survey to independent stratified random samples of university students. After 27 months of consecutive survey administration, we have developed and refined a survey protocol that has proven effective in engaging students at four Canadian institutions, allowing us to track how mental health and substance use vary over time using an internationally developed university student survey based on the criteria from the Diagnostic and Statistical Manual of Mental Disorders (Fifth Edition).

**International Registered Report Identifier (IRRID):**

RR1-10.2196/35168

## Introduction

The university years coincide with a critical developmental period, a time when many common mental disorders emerge [1]. This developmental period, coupled with life changes that often accompany the transition to university life, such as separation from family and friends, a new environment, and increasing academic pressure, is likely to contribute to increased rates of mental and substance use problems and disorders, potentially overwhelming available services [2-5]. In response to this public health concern, the World Health Organization (WHO) World Mental Health International College Student (WMH-ICS) initiative was developed. The initiative aims to screen for mental health and substance use problems among college and university students on a global scale as well as to develop and evaluate evidence-based preventive and ameliorative interventions for this population. The epidemiologic surveys—a core component of the WMH-ICS initiative—are self-administered online questionnaires that generate diagnostic estimates for a wide range of common mental disorders and include questions that probe symptom severity, help-seeking behaviors, and treatment barriers [6]. To date, the WMH-ICS survey has assessed 95,000 students across 16 countries worldwide [7] and has been repeatedly used in scientific studies [8-13]. Although procedures vary by country and site depending on local resources and priorities, all WMH-ICS surveys include core questions about stressors, disorders, impairments, and treatment.

In February 2020, the Canadian version of the WMH-ICS survey initiative was deployed at the University of British Columbia (UBC). This was followed by deployment at Simon Fraser University (SFU; November 2020), McMaster University (January 2021), and the University of Toronto (UofT; January 2022). Additional Canadian sites are currently in various stages of assessment and implementation. The methodology of these Canadian surveys is notably distinct from the traditional WMH-ICS procedures, which invite all potential respondents (typically first-year students) at one time point and often incorporate follow-up surveys later in their university career. In comparison, the Canadian version is deployed as a repeated cross-sectional survey, where a new stratified random sample of students is invited to participate weekly (additional details provided in the Methods section). Whereas all WMH-ICS surveys aim to provide a detailed picture of the common mental and substance use problems and disorders affecting students, the ongoing administration of the Canadian survey to representative samples of the student population offers the added benefit of providing time-based data that can be used to better understand how these problems vary throughout the year. These data can also be used to understand variation in response to external stressors such as exam periods or global events like the COVID-19 pandemic. The ongoing nature of the survey in Canada also provides the opportunity to add new survey content (eg, related to the COVID-19 pandemic) or revise questions based on existing data and student feedback. Another strength of the Canadian survey design is the use of a multitiered recruitment process, where a subsample of initial nonresponders is selected for follow-up through alternative recruitment strategies, leading to high response rates that minimize nonresponder bias. Finally, the Canadian survey stratifies respondents by level of need and provides tailored service recommendations, including facilitating a follow-up call within 24 hours and a counselling interview within 5 days for those at increased risk of suicide.

This protocol paper presents the Canadian version of the WMH-ICS survey, detailing the adapted survey instrument, the weekly cross-sectional administration, the multitiered recruitment strategy, and the associated risk mitigation protocols. In doing so, it aims to provide a methodological resource for researchers interested in better understanding how mental health and substance use vary over time, with an ultimate goal of improving prevention and intervention efforts for mental health problems among university students. In addition, it aims to serve as a useful guide for those interested in replicating the outlined cross-sectional methodology or conducting cross-national comparisons of WMH-ICS data.

## Methods

### Survey Instrument and Revisions

This survey is administered as part of the WHO WMH-ICS initiative [6] and is a web-based survey tool designed to assess mental health and substance use in postsecondary students. On average, the survey takes students approximately 20 minutes to complete. Questions assess students’ well-being (ie, physical and mental health, stresses, self-harm, and suicidal thoughts and behaviors), substance use (ie, alcohol and street drugs), health and social functioning (ie, impairments to daily activities), treatment (ie, use of health services, barriers to help seeking, and readiness for change), and respondent characteristics (eg, sociodemographics or childhood experiences).

The WMH-ICS survey instrument uses validated screening scales to generate lifetime and 12-month prevalence estimates for the following common disorders from the *Diagnostic and Statistical Manual of Mental Disorders*: major depressive disorder, bipolar disorder, generalized anxiety disorder, panic disorder, alcohol use disorder, and substance use disorder [8]. Optional disorder sections used in this survey include screening questions for intermittent explosive disorder, social anxiety disorder, posttraumatic stress disorder, eating disorders, and attention-deficit/hyperactivity disorder [8,14]. While previous publications from the WMH-ICS initiative have used *Diagnostic and Statistical Manual of Mental Disorders* (Fourth Edition) diagnostic criteria [15], a *Diagnostic and Statistical Manual of Mental Disorders* (Fifth Edition) version was recently developed, which is being implemented for the first time in the Canadian survey [7,16]. As is the case with all WMH-ICS surveys, disorder assessments are based on the Composite International Diagnostic Interview Screening Scales [17,18] and the Alcohol Use Disorders Identification Test [19].

In September 2019, the survey was adapted to the Canadian context by a research team based at UBC. During the adaptation process, alcohol and substance use questions were added to generate 30-day estimates, as well as to assess adherence to Canadian low-risk drinking and cannabis use guidelines [20-23]. Additional questions regarding opioid use were also added to obtain information about adherence to the Canadian Opioid Prescribing Guidelines [24]. Additional questions were included on topics of interest to the research team, including past use and willingness to use e–mental health services. A French translation of this Canadian survey was developed using the WMH-ICS France survey as the base content, with adjustments made by native Québécois French speakers to account for the Canadian context and associated regional language variations.

As the survey is deployed on a weekly basis, there is an ongoing opportunity to respond to student feedback. While feedback is not formally requested from respondents, many students from across study sites may contact the principal investigator and research team via email to share feedback on how to improve the survey. Each suggestion is evaluated by the research team to examine feasibility and alignment with the other WMH-ICS surveys. The research team gains additional student feedback by convening a student advisory board consisting of a group of approximately 13 student representatives from organizations, groups, or clubs focused on mental health and substance use issues on campus. Student consultations and the resulting survey adaptations are focused on the language and phrasing of survey questions, ensuring that the survey is inclusive and applicable to the diverse student body (eg, expanding gender identity response options and including content warnings ahead of questions relating to potentially traumatic past experiences and health impacts of COVID-19). Indeed, the flexibility of running weekly surveys allows the research team to keep up with current institutional, local, and global events such as the pandemic. Questions pertaining to COVID-19 were added to the survey more than a month before the declaration of a British Columbia Provincial public health emergency and have allowed continuous measurement of students’ exposure to and experience of COVID-19 symptoms, feelings of stress associated with the pandemic, consequences of the pandemic (ie, death of relations/acquaintances and disruption to daily life, housing situations, and classes), the transition to remote learning and then return to in-person learning, and vaccination state. The current versions of the survey can be found in Multimedia Appendices 1 and 2 (English and French, respectively).

### Ethics

The authors assert that all procedures contributing to this work comply with the ethical standards of the relevant national and institutional committees on human experimentation and with the Helsinki Declaration of 1975, as revised in 2013 [25]. All procedures were approved by the UBC Behavioural Research Ethics Board in a harmonized review process with SFU (approval number H19-02538), the McMaster University Research Ethics Board Office (approval number 3695), and the UofT Research Ethics Board (approval number 39919). Initial approval for the survey was acquired in 2019, and all revisions in both English and French have been approved on an ongoing basis. Written informed consent is obtained from all participants (Multimedia Appendix 3).

### Sampling

The following section outlines a general approach to the sampling process. There are slight variations between institutions based on site-specific regulations and preferences for data management. The proposed study duration is 1 year but can be extended by sites based on interest and funding availability. The anticipated sample size is approximately 5500 students per site per year.

Prior to survey launch and at the start of every semester for the duration of the study, a *data pull* process is initiated in which a deidentified data set of students is provided to the research team by the university registrar containing randomly assigned alphanumeric IDs and stratifying variables. All actively enrolled students are included in the data set, with the only exclusion criteria being previously sampled students. Using the software SAS (SAS Institute Inc), independent stratified random samples of 350 IDs are drawn for each week of the semester. The stratifying variables—degree type, year of study, international or Canadian student status, gender, and age—are employed in this process to ensure that each weekly sample is representative of the student population, as well as to provide the opportunity to statistically weight final data to reflect the population from which the samples are drawn.

Samples are randomly ordered using Excel’s (Microsoft Corporation) random number generator and assigned a week for implementation. The samples are then sent back to the university registrar, and files containing the selected students’ first names, email addresses, and phone numbers are returned. To ensure confidentiality of student data, these files returned to the research team do not contain any of the stratifying variables, which are never linked nor linkable by the research team to the student’s name, email, or phone. For each sample, a cross check of five random IDs and email addresses is performed by a second university registrar employee to confirm proper data set linkage. This process is repeated each semester and is initiated once the academic deadline for course withdrawal/drop has passed. During each data pull, several additional samples are drawn in case there are processing delays in the subsequent semester, so the additional samples can be used in the interim to prevent disruption to the weekly data collection process.

### Online Survey Delivery

The screening tool is programmed into Qualtrics software [26]. The survey is administered from Canadian Qualtrics licenses that store data in cloud-based servers located in Canada. The survey is initially programmed in English, and the Canadian French translation is manually inputted for each question in the “Translate Survey” page of Qualtrics. The “Look and Feel” tool is used to personalize the logo and color scheme of surveys for their respective institutions. Responses are anonymized so that respondents’ IP addresses, location data, and contact information are not recorded. Survey access is set to invitation only, and the “prevent multiple submissions” setting is turned on. In Qualtrics, a folder is added for each weekly cohort invited to the survey. Each weekly folder contains three identical copies of the survey: the initial survey, filled by students who responded to the email invitations (I); the survey for initial nonresponders with a phone number on record, also known as “hard to reach” students (“NRa Phones”); and the survey for initial nonresponders without a phone number on record, also known as “very hard to reach” students (“NRb No Phones”).

### Survey Administration

Every Sunday, a new independent sample of 350 students receives an email invitation to participate in the survey through the online platform. The invitation is codeveloped and cosigned by the principal investigator and a senior-level administrator in charge of the student services portfolio (eg, vice president, students) to convey that the endeavor is a research and quality improvement undertaking with organizational support. After 3 and 7 days, those who have not completed the survey receive similar reminder emails. Three days after the second reminder (ie, 10 days after the initial invitation), the initial survey closes, the distribution history is downloaded, and a random sample of 70 nonresponders is drawn. These students are deemed “hard to reach” and are assigned for follow-up.

Selected students who have a valid North American phone number on file with the university registrar are sent an email that notifies them of their selection for follow-up and includes a new survey link (to the NRa Phones survey). In addition, it informs them that they will be called the following day, with an option to opt-out of receiving the phone call. The next morning, the students are called by a research assistant who explains the importance of the student’s participation to increase the validity of the results by including initial nonresponder data, answers any questions, and offers to resend the survey link in case it had been lost or deleted. If the phone call is not answered, the research assistant hangs up and makes a second call several hours later on the same day. If this second call is not answered, a voicemail is left that contains similar content to the phone call, and a callback number is provided in case the student had any questions. In the event that a voicemail system is not set up, a SMS text message is sent with an abbreviated version of the message.

**Figure 1:**
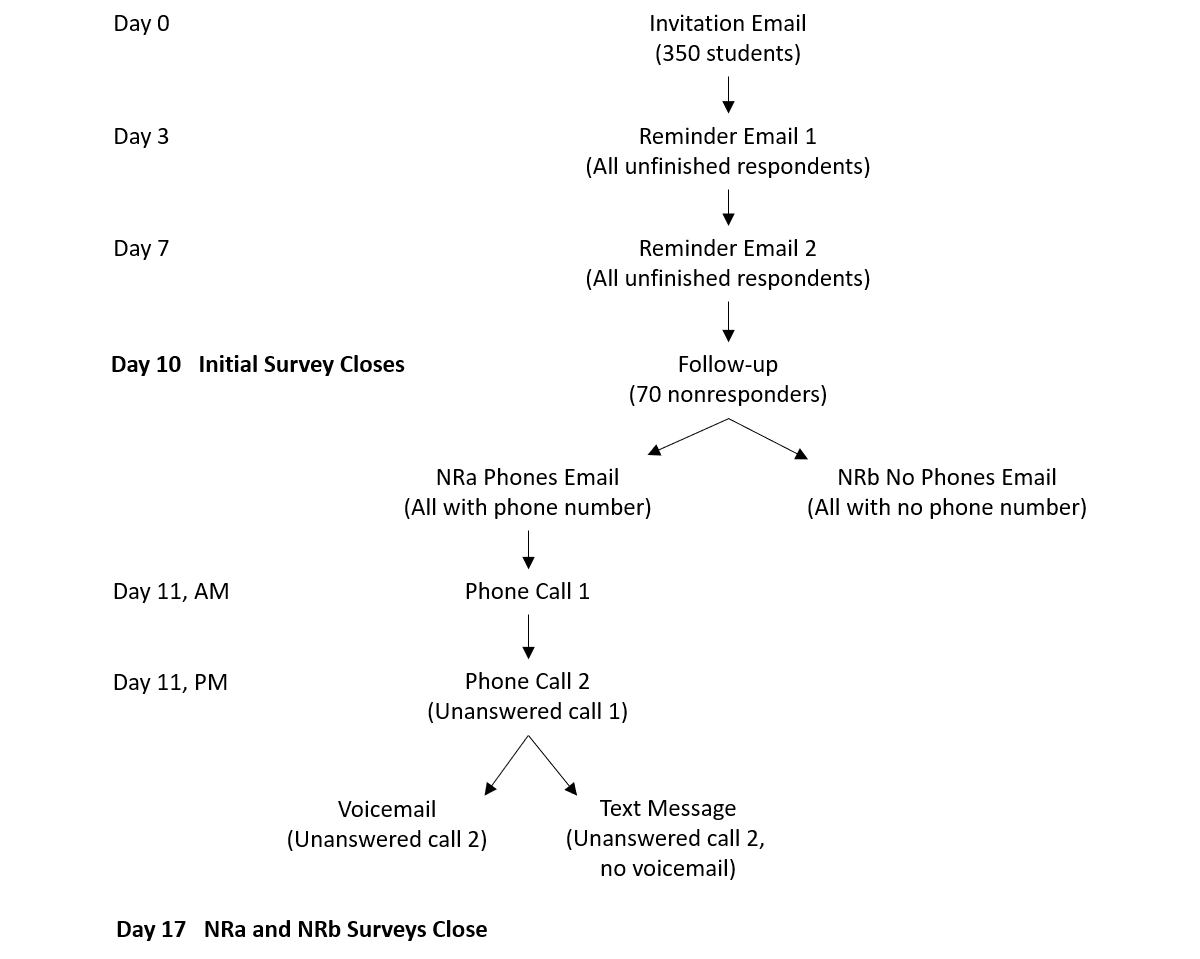
A visual representation of the survey administration process for each weekly sample. NRa: hard-to-reach students; NRb: very hard-to-reach students.

Selected students who do not have a valid North American phone number on file are sent a personalized email from the principal investigator that explains the importance of their response and includes a new survey link to the NRb No Phones survey. These surveys for “hard to reach” and “very hard to reach” students (NRa Phones and NRb No Phones) close 7 days after the follow-up email that contained the new survey link. This process is outlined as a flowchart (Figure 1), and sample email templates and call, voicemail, and text scripts are included in Multimedia Appendix 4.

### Risk Mitigation Protocol

Upon completion of the survey, all students are redirected to a new page on the Qualtrics platform that provides them with a targeted list of resources based on their survey responses. These lists of resources are from 1 of 6 prespecified protocols and are developed by the research team in collaboration with each study site’s university health and counseling services. The protocols are programmed in a hierarchical fashion, with lower numbered protocols taking precedence over all those numbered higher (ie, each respondent triggered only 1 of these protocols as per the hierarchy). A summary of each protocol is provided below (with reference to the specific survey questions used to identify risk), and the full risk mitigation protocol with textboxes and emails can be found in Multimedia Appendix 5.

#### Protocol 1: Protocol for Participants With Increased Likelihood of Acting on a Recent Suicide Plan in the Coming Year

Participants acknowledging any suicide plan during the past 12 months (response to question G10 different than 0) plus reporting that acting upon such a plan during the coming 12 months was “somewhat likely” or “very likely” (question G11) are presented upon survey completion with a textbox that contains (a) information on relevant clinical resources and support services, as well as (b) a request for the student’s consent and permission to contact the university counseling services on their behalf to obtain an expedited appointment. They also receive this information in email format immediately after completing the survey and 28 days later.

If the student consents and provides their contact details, an email containing this information is automatically sent to the designated university counseling services’ point person by the online platform. If the student chooses to consent and provide their contact details by replying to the emailed resource list, their reply goes to the designated “Protocol 1 email inbox” and is then automatically forwarded to the designated university counseling services’ point person. As a fail-safe mechanism, the principal investigator of the project also receives an automated email notifying them that a Protocol 1 has been triggered, but their email does not contain the student’s name or contact details.

The designated counseling services employee reaches out to the student within 1 business day of receiving the email and offers them an appointment within 1 week. This outreach includes up to two phone calls. If the second phone call is unanswered, a voicemail is left. For students without a voicemail setup, an email is sent to the address they provide. After completing this outreach, the point person emails the principal investigator to confirm that outreach occurred, identifying the protocol by the date and time it is triggered to maintain student anonymity. If student services does not reach out to confirm to the research team that contact had been attempted or established, the research team follows up with student services to confirm that the automatic Protocol 1 email had been received.

#### Protocol 2: Protocol for Participants With History of Manic Episodes

Students screening positive for or reporting a lifetime manic episode (shown question E27 or response to question B5b is “yes”) receive an automated textbox and email upon survey completion that provides information on the clinical resources and support services available for this level of need as per each site’s service provision protocols.

#### Protocol 3: Protocol for Participants With a History of Psychosis

Students reporting a lifetime history of psychotic experiences (response to question E46 or question E47 is “yes”) receive an automated textbox and email upon survey completion that provides information on the clinical resources and support services available for this level of need as per each site’s service provision protocols.

#### Protocol 4: Protocol for Participants With History of Suicide Attempts, a Recent Suicide Attempt (Past Year), Suicide Plan During the Past 12 Months, or Suicidal Ideation During the Past 30 Days

Participants indicating any lifetime history of suicide attempts (response to question G16 is “yes”), any suicide plan during the past 12 months (response to question G10 different than 0), any suicide attempt during the past 12 months (response to question G19 different than 0), or any suicidal ideation during the past 30 days (response to question G6 different than “None of the time”) receive an automated textbox and email upon survey completion that provides information on relevant clinical resources and support services.

#### Protocol 5: Protocol for Participants With Severe Impairment Resulting From Any Mental or Substance Use Disorder

Students screening positive for severe impairments resulting from any mental or substance use disorder (response to question B3b, B3c, B4b, or B4c is “Very severe interference” or “severe interference”) receives an automated textbox and email upon survey completion that provides information on the clinical resources and support services available for this level of need as per each site’s service provision protocols.

#### Protocol 6: Standard General Protocol

As a standard general precaution, all participants who do not trigger a specific protocol are shown a textbox that contains information on general mental health and wellness resources and support services. These students are provided the option to have the resources emailed to them if they click on the link to a new online form and input their email address.

Each of the 6 risk mitigation protocols described above are programmed as embedded data in the online platform, and the resource list emails are sent through an automated email trigger function. Emails are programmed to be sent to the respondent using piped text (a line of code that pulls information from different sources; eg, responses to previous questions or embedded data) and selecting “Recipient Email” under “Panels Field” in Qualtrics. Conditions are added to specify the protocol triggered and the language used to complete the survey. Textboxes are programmed into a different survey file, which respondents are redirected to upon completion of the main survey through a query string URL inserted as a “custom end of survey message.” The Protocol 1 text includes a checkbox requesting students’ consent to contact university health services on their behalf, and a form that requests name, email, and phone numbers. If a student consents by ticking the checkbox, the form responses are emailed to university counseling services using piped text and the email trigger function described above. The form questions are set to “exclude from analysis” so that identifying information is not saved as data and is not accessible to the research team. To deploy the postsurvey protocols, there is one textbox redirect file per institution to ensure only institutionally and locally relevant resources are shown to students.

### Data Download

Upon survey closure, the data are exported from the digital platform in SPSS format (IBM Corp). As 3 surveys close each week, 3 SPSS files are uploaded weekly to a secure institutional storage platform. Only specific team members who need access to the data folders are given access, as per the Data Security Protocols (see below).

### Data Security

All data—including student contact information and survey responses—related to the project are stored on a secure file storage platform endorsed by the respective university. The secure platforms are stored and hosted in Canada, meeting the Freedom of Information and Protection of Privacy Act’s data residency requirements. To ensure that all data are handled securely, the Mental Health Systems and Services Laboratory generated a “Data Management Guidelines” document, which outlines the stringent processes that need to be followed when accessing, storing, and downloading data. As the project works with both student survey responses and student contact information on a weekly basis, it is vital to uphold a high level of security across study sites. Of note, the survey responses are always anonymous (as opposed to anonymized or deidentified) since at no point are the survey responses linked to the student’s name or any identifying administrative data.

The primary tenet for data security is that all data access is on a need-to-know basis. This rule is reflected in all facets of data management in the survey administration process and culminates in the data deletion process. When all the survey administration and calls are completed for the week, all documents containing student contact information are deleted while screen recording. This includes any downloaded distribution histories, call history, documents provided by the university registrar, etc. The deletion videos are then uploaded to the secure platform for the purpose of auditing by the research team and university administrators.

### Remuneration

At most sites, survey participants are entered into a yearly draw for a CAD $1,000 (approximately US $750) gift card. There is one winner per institution per calendar year. The remuneration strategy is open to modification (eg, more draws worth less value) if requested by an institutional research ethics board.

### Statistical Analysis Plan

Survey data will be examined independently for Canadian-specific analyses and as part of WMH-ICS cross-national studies. Analyses will aim to provide weighted prevalence estimates for a range of mental disorders, such as major depressive disorder, bipolar disorder, generalized anxiety disorder, panic disorder, alcohol use disorder, and drug use disorder. In addition, associations of demographic determinants and past experiences with meeting criteria for various disorders and suicidality, as well as with accessing mental health services will be evaluated with multivariable regression models. Canadian-specific analyses will be undertaken to explore changes in disorder and symptom prevalence across the calendar and academic years, as well as in response to specific events (eg, natural disasters or pandemics). In line with the WMH-ICS initiative’s goals, analyses will also seek to identify areas of unmet treatment need, particularly for marginalized and racialized groups.

## Results

Funded in July 2019 by Health Canada, the Canadian version of the WMH-ICS survey was ultimately launched at UBC on February 9, 2020. SFU followed suit on November 29, 2020; McMaster University on January 10, 2021; and the UofT on January 16, 2022. As of May 6, 2022, 29,503 responses have been collected (UBC: n=12,990; SFU: n=8886; McMaster University: n=6505; and UofT: n=1122). Data analysis is ongoing, with the first paper on anxiety and depression during COVID-19 published in March 2021 and additional manuscripts in various stages of the preparation and submission process.

## Discussion

In 27 months, the Canadian version of the WMH-ICS survey has provided data from nearly 30,000 students, enabling us to estimate prevalence for a wide range of mental disorders and assess variation throughout the year and in response to specific events. While this survey is grounded in the international WMH-ICS initiative, several methodological features distinguish it from previous work. The weekly administration to independent stratified random samples of university students is a novel approach within the international initiative and is allowing us to track how mental health and substance use vary over time, which has proven to be particularly enlightening in the context of the COVID-19 pandemic that has unfolded during the past 2 years. In addition, the locally developed risk mitigation protocols associated with our survey enables us to offer targeted resources to students who report mental health concerns and facilitate connections to services for students at increased risk of suicide. This feature, facilitated by the digital nature of the survey, provides real-time feedback and an opportunity for preventative efforts to minimize the risk of adverse outcomes such as suicide. These innovative features are in addition to the strengths of the broader WMH-ICS initiative, such as the use of a validated screening tool that has proven effective in engaging students on a global scale.

The Canadian survey data will provide a comprehensive and comparative picture of the common mental and substance use problems affecting students, assist us in identifying the best and most timely ways to engage university students through cost-effective e–mental health interventions, and allow us to explore novel health systems integration strategies between digital and brick-and-mortar services. Collecting data from multiple institutions across Canada as part of the WMH-ICS initiative enables this survey to contribute to knowledge building and service planning on a local, national, and international level. Specifically in Canada, the data collected will contribute to the development of e–mental health intervention resources. We intend for this protocol paper to serve as a guide for institutions considering ongoing mental health and substance use online screening protocols.

## Appendix

Multimedia Appendix 1 English survey instrument.

Multimedia Appendix 2 French survey instrument.

Multimedia Appendix 3 Study cover letter and consent form.

Multimedia Appendix 4 Sample recruitment emails and follow-up scripts.

Multimedia Appendix 5 Risk mitigation protocol.

## Abbreviations

SFU: Simon Fraser University
UBC: The University of British Columbia
UofT: University of Toronto
WHO: World Health Organization
WMH-ICS: World Mental Health International College Student
