# Supplementary material for: The World Mental Health International College Student Survey in Canada: Protocol for a Mental Health and Substance Use Trend Study
Source: JMIR Res Protoc. 2022 Jul 29;11(7):e35168. doi: 10.2196/35168 (PMC9377443; doi:10.2196/35168)
Supplement: Multimedia Appendix 1 [file resprot_v11i7e35168_app1.pdf]

Multimedia Appendix 1. English survey instrument.

DSM-5 World Mental Health

University Student Survey

Baseline

SHORT FORM

VERSION 2

September 24, 2019

Ronald C. Kessler, Ph.D.
McNeil Family Professor of Health Care Policy
Harvard Medical School

Revised and adapted for Canadian students by Daniel Vigo

July 16, 2021

____

Comments to: RC Kessler, Department of Health Care Policy, Harvard Medical School, 180 Longwood Avenue, Boston, MA, USA 02115; kessler@hcp.med.harvard.edu; 617-432-3587 (Voice); 617-432-3588 (Fax)

SECTION ORDER:

SECTION A: YOUR BACKGROUND
SECTION B: YOUR HEALTH
SECTION D: ATTENTION AND CONCENTRATION
SECTION E: EMOTIONAL PROBLEMS
SECTION F: ALCOHOL AND DRUGS
SECTION G: SELF-HARM
SECTION H: SEEKING TREATMENT
SECTION I: CHILDHOOD BACKGROUND
SECTION J: RECENT EXPERIENCES
SECTION K: SEXUALITY

Note: **Please DO NOT hit your internet browser's back arrow as you will be ejected from the survey**. You can use the blue "Back" button within the survey if available. If it is not present, you are unable to go back to the previous question.

SECTION A: YOUR BACKGROUND

A1. How old are you?

[DROPDOWN LIST] 16 or younger, 17, … , 35, 36 or older

A2. What was your sex at birth?

- ◯ Male
- ◯ Female

A2a. What is your current gender?

- ◯ Male
- ◯ Female
- ◯ Non-binary
- ◯ Two-spirit
- ◯ Other *(Briefly describe in the text box below)*

____

A3. What is your current student status?

- ◯ Undergraduate degree, 1st year
- ◯ Undergraduate degree, 2nd year
- ◯ Undergraduate degree, 3rd year
- ◯ Undergraduate degree, 4th year
- ◯ Undergraduate degree, 5th year
- ◯ Graduate degree student
- ◯ Non-degree student
- ◯ Recently graduated
- ◯ Other *(Briefly describe in the text box below)*

____

A3a. What is the status of the courses you are currently taking?

- ◯ Entirely online
- ◯ A combination of online and in-person
- ◯ Entirely in-person
- ◯ I'm not taking courses right now
- ◯ Other *(Briefly describe in the text box below)*

____

A4. What is your race, ethnicity, or heritage? *(Check all that apply.)*

- ☐ White
- ☐ Hispanic or Latino
- ☐ Black
- ☐ First Nations, Métis, or Inuit

- ☐ South Asian (e.g., East Indian, Pakistani, Sri Lankan, etc.
- ☐ Southeast Asian (e.g., Vietnamese, Cambodian, Laotian, Thai, etc.)
- ☐ West Asian (e.g., Afghan, Iranian, etc.)
- ☐ Chinese
- ☐ Filipino
- ☐ Arab
- ☐ Korean
- ☐ Japanese
- ☐ Other *(Please briefly describe)*

____

A5. What is your primary language? (*Check all that apply.*)

- ☐ English
- ☐ French
- ☐ Other language *(Please briefly describe)*

____

A6. Were you born in Canada?

- ○ Yes → GO TO A8
- ○ No

[PROGRAMMER: SKIP MISSING WITH “YES”]

A7. How old were you when you <u>first</u> moved to Canada? *(Give your best estimate if you cannot remember your exact age. If less than one year old, select “4 or younger.”)*

[PROGRAMMER NOTE: IF R SELECTS NUMBER GREATER THAN CURRENT AGE, OR IF BA1 = MISSING, ADD POP UP BOX “PLEASE SELECT A NUMBER NO GREATER THAN YOUR CURRENT AGE.”]

[DROPDOWN LIST] 4 or younger, 5, …, 35, 36 or older

A8. How many of your parents were born in Canada?

- ○ Both
- ○ One
- ○ Neither

A8a. Are you currently living in Canada?

- ○ Yes
- ○ No

A9. What’s your current housing situation?

- ○ With parents or other relatives
- ○ In your own home or apartment (owned or rented)
- ○ In a university owned or operated residence
- ○ In a fraternity or sorority house

- ◯ In a shared house, apartment, or flat
- ◯ Other *(Please briefly describe)*

____

CKPT.A10a.

1. A8a = "YES" OR MISSING, GO TO A10a.
2. A8a = "NO", GO TO A10b.

A10a. Please provide the postal code of the area where you are currently living.

____

GO TO B1

A10b. Please choose the region that best describes where you are currently living:

[DROPDOWN LIST] North America, Central America and the Caribbean, South America, Western Europe, Central Europe, Eastern Europe, Central Asia, East Asia and Pacific region, South Asia, Sub-Saharan Africa, North Africa

END OF SECTION

SECTION B: YOUR HEALTH

The following section is about your health. It includes questions about COVID-19 with the goals of understanding its impact on the well-being of our community, and improving the health system's response. All answers are anonymous and cannot be traced back to respondents. If these questions are stressful or you prefer not to answer, you may skip them. At the end of the survey you will find mental health resources available to you in the context of COVID-19.

B1. How would you rate your overall physical health?

- ○ Excellent
- ○ Very good
- ○ Good
- ○ Fair
- ○ Poor

B2. How would you rate your overall mental health?

- ○ Excellent
- ○ Very good
- ○ Good
- ○ Fair
- ○ Poor

B3. During the <u>past 12 months</u>, how much did each of the following kinds of problems interfere with your performance at school?

		Very severe interference	Severe	Moderate	Mild	No interference
a.	Physical health problems	○	○	○	○	○
b.	Mental or emotional health problems (e.g., anxiety, depression)	○	○	○	○	○
c.	Alcohol or drug use problems	○	○	○	○	○

B4. During the <u>past 12 months</u>, how much did each of the following kinds of problems interfere with your personal or social life?

		Very severe interference	Severe	Moderate	Mild	No interference
a.	Physical health problems	○	○	○	○	○
b.	Mental or emotional health problems (e.g., anxiety, depression)	○	○	○	○	○
c.	Alcohol or drug use problems	○	○	○	○	○

B5. Have you <u>ever in your life</u> had any of the following emotional problems?

		Yes	No
a.	Depression	○	○
b.	Manic-depression, mania, or bipolar disorder	○	○

c.	Panic attacks	○	○
d.	Problems with anxiety (nerves, worries, fears, compulsions, obsessions)	○	○
e.	Any other serious emotional problem	○	○

B6. In the <u>past 30 days</u>, how often did you have each of the following?

		All or almost all of the time	Most of the time	Some of the time	A little of the time	None of the time
a.	Pain in your back, neck, arms, legs, or joints (knees, hips, etc.)	○	○	○	○	○
b.	Headaches	○	○	○	○	○
c.	Pain in any other part of the body	○	○	○	○	○
d.	Muscle tension	○	○	○	○	○
e.	Dizziness	○	○	○	○	○

B7. Health Canada indicates the following are common symptoms of COVID-19: cough, difficulty breathing, fever, chills, fatigue, body aches, headaches, loss of smell or taste, and gastrointestinal symptoms. Have you had any of these symptoms since January, 2020?

○ Yes, and I tested positive for COVID-19 (or was diagnosed with COVID-19 by a health provider)
○ Yes, and I tested negative for COVID-19
○ Yes, but I didn't or couldn't get tested for COVID-19
○ No

CKPT.B7a.

1. B7 = "NO," GO TO B8
2. ALL OTHERS GO TO B7a

B7a. Have you (B7= "YES, AND I TESTED POSITIVE FOR COVID-19 (OR WAS DIAGNOSED WITH COVID-19 BY A HEALTH PROVIDER)": tested positive or been diagnosed/B7= "YES, AND I TESTED NEGATIVE FOR COVID-19": tested negative/ALL OTHERS: had these symptoms) in the past two weeks?

○ Yes
○ No

CKPT.B7b.

1. B7 = "YES, BUT I DIDN'T OR COULDN'T GET TESTED FOR COVID-19," GO TO B7b
2. ALL OTHERS GO TO B7c

B7b. Do you think you <u>definitely</u> had COVID-19, <u>probably</u> had it, possibly, probably <u>did not</u> have it, or <u>definitely</u> did not have it?

○ Definitely had it
○ Probably had it
○ Possibly
○ Probably did not
○ Definitely did not

B7c. How were your symptoms treated?

- ◯ Hospitalized and intubated (i.e., put on a ventilator)
- ◯ Hospitalized but not intubated (i.e., not put on a ventilator)
- ◯ Quarantined or isolated at home under orders of a health care professional
- ◯ Given instructions on self-treatment and told to avoid public places but not told to quarantine or isolate
- ◯ Not treated

B8. Do you know anyone who you think tested positive for COVID-19 or was diagnosed with COVID-19 by a health provider? *(If you know people in more than one place, choose the option closest to where you are currently living.)*

- ◯ Yes, I know someone in the city I am living in
- ◯ Yes, I know someone in the country I am living in, but not the city
- ◯ Yes, I know someone but not in the country I am living in
- ◯ No

CKPT.B9.

1. B8 = "NO," GO TO B11
2. ALL OTHERS GO TO B9

B9. How many people of each type were infected with COVID-19?

		**Number of people**
a.	Someone living with you at the time	
b.	Any close loved one not living with you at the time (e.g., parent, child, sibling, partner other relative)	
c.	Someone working with you	
d.	Someone taking a course with you	
e.	Close friend not living, working, or doing coursework with you at the time	
f.	Anyone else you know (e.g., neighbor, acquaintance)	

B10a. Have they been diagnosed with COVID-19 in the past two weeks? *(If you know more than one person who has been positive for COVID-19 in the past two weeks think about the individual with the most severe symptoms.)*

- ◯ Yes, and they have been hospitalized for severe symptoms
- ◯ Yes, and they have/had mild-moderate symptoms
- ◯ Yes, and they were asymptomatic
- ◯ No

CKPT.B10b.

1. B9a = 1 OR MORE OR B9b = 1 OR MORE OR B9c = 1 OR MORE OR B9d = 1 OR MORE OR B9e = 1 OR MORE OR B9f = 1 OR MORE, GO TO B10b
2. ALL OTHERS GO TO B11

B10b. (B9a + B9b + B9c + B9d + B9e +B9f = 1: Did this person die from COVID-19?/B9a + B9b + B9c + B9d + B9e + B9f = 2: Did either of these people die from COVID-19?/ALL OTHERS: Did any of these people die from COVID-19?)

- ◯ Yes
- ◯ No

CKPT.B10c.

1. B10b = "YES," GO TO B10c
2. ALL OTHERS GO TO B11

B10c. Who died from COVID-19? *(Check all that apply.)*

- ☐ One of your parents
- ☐ Your significant other, partner, or spouse
- ☐ Any other close relative
- ☐ A close friend
- ☐ Someone you work with
- ☐ Anyone else you know (e.g., neighbor, acquaintance)

B11. Does anyone (B9a > "0" : else) in your residence/house/apartment have symptoms such as a cough, difficulty breathing, fever, chills, fatigue, body aches, headaches, loss of smell or taste, or gastrointestinal symptoms?

- ◯ Yes, but they tested negative for COVID-19
- ◯ Yes, but they did not get tested or I am not aware of whether they got tested
- ◯ Not that I know of

CKPT.B12.

1. A3a = "ENTIRELY IN-PERSON" OR "A COMBINATION OF ONLINE AND IN-PERSON", GO TO B12
2. ALL OTHERS GO TO B13

B12. Does anyone (B9d > "0": else) in your courses have symptoms such as a cough, difficulty breathing, fever, chills, fatigue, body aches, headaches, loss of smell or taste, or gastrointestinal symptoms?

- ◯ Yes, but they tested negative for COVID-19
- ◯ Yes, and I am not aware of whether they got tested
- ◯ Not that I know of

B13. Has the COVID-19 pandemic affected your emotional wellbeing?

- ◯ No
- ◯ Yes, but I was able to manage it
- ◯ Yes, I was/am overwhelmed and couldn't/can't find help
- ◯ Yes, I was/am overwhelmed but I was/am able to get help

B14. Compared to before the COVID-19 pandemic, how "normal" would you say your life is now, with 0 being "completely disrupted" and 100 being "completely normal"?

Completely disrupted								Completely normal		
**0**	**10**	**20**	**30**	**40**	**50**	**60**	**70**	**80**	**90**	**100**

B15. Think of the month when your stress related to the COVID-19 outbreak was greatest. During <u>that month</u>, how much stress did the outbreak cause due to the following problems?

		Very severe stress	Severe	Moderate	Mild	No stress
a.	Family finances (e.g., lost income or investments)	○	○	○	○	○
b.	Increased social isolation	○	○	○	○	○
c.	Difficulty getting needed help to your loved ones	○	○	○	○	○
d.	Increased arguments with your family or friends	○	○	○	○	○
e.	Worry about you getting infected	○	○	○	○	○
f.	Worry about loved ones getting infected	○	○	○	○	○

B16. What is your vaccination status?

○ I am fully vaccinated
○ I've had the first of two doses
○ I will get vaccinated as soon as I can
○ I choose not to get vaccinated
○ I cannot get vaccinated due to disease, allergy, or treatment

END OF SECTION

SECTION D: ATTENTION AND CONCENTRATION

D1. The next question is about difficulties with organization, concentration, or impulsivity. How often did you have each of the following difficulties in the <u>past 6 months?</u>

	Very often	Often	Sometimes	Rarely	Never
a. You avoided or delayed getting started when you had a task that required a lot of thought	○	○	○	○	○
b. You had problems remembering appointments or obligations	○	○	○	○	○
c. You had difficulty getting things in order when you had to do a task that required organization	○	○	○	○	○
d. You had trouble wrapping up the final details of a project once the challenging parts were done	○	○	○	○	○
e. You felt overly active and compelled to do things, like you were driven by a motor	○	○	○	○	○
f. You fidgeted or squirmed with your hands or feet when you had to sit down for a long time	○	○	○	○	○

CKPT.D2.

1. THREE OR MORE RESPONSES IN D1a-f SERIES = AT LEAST "SOMETIMES," GO TO D2
2. ALL OTHERS GO TO NEXT SECTION

D2. About how old were you the <u>very first time</u> you had problems with organization, concentration, or impulsivity?

[DROPDOWN LIST] 4 or younger, 5, …, 35, 36 or older

END OF SECTION

SECTION E: EMOTIONAL PROBLEMS

E1. The next questions are about emotional difficulties you might have experienced at some time in your life.

(B5a = "YES": Earlier in the survey you reported having a history of depression. Think about the times lasting 2 weeks or longer when you had the strongest feelings of this sort. During those times, how often did you have each of the following experiences?/ALL OTHERS: Virtually everyone has times in their life when they feel sad, depressed, or discouraged about how things are going in their life. Think about the times lasting 2 weeks or longer when you had the strongest feelings of this sort. During those times, how often did you have each of the following experiences? *(If you are one of the few people that never had such times, mark "none of the time" to all the following questions.)***)**

		All or almost all the time	Most of the time	Some of the time	A little of the time	None of the time
a.	Feel sad or depressed?	○	○	○	○	○
b.	Feel discouraged about how things were going in your life?	○	○	○	○	○
c.	Take little or no interest or pleasure in things?	○	○	○	○	○
d.	Feel down on yourself, no good, or worthless?	○	○	○	○	○

CKPT.E2.

1. (E1a OR E1b = AT LEAST "MOST OF THE TIME") OR (E1c = AT LEAST "MOST OF THE TIME"), GO TO E2
2. E1a = "NONE OF THE TIME" AND E1b = "NONE OF THE TIME" AND E1c = "NONE OF THE TIME" AND E1d = "NONE OF THE TIME," GO TO E7
3. ALL OTHERS GO TO E6

E2. During those times, how often did you have each of the following experiences?

		All or almost all the time	Most of the time	Some of the time	A little of the time	None of the time
a.	Think a lot about death (either your own, someone else's, or death in general)?	○	○	○	○	○
b.	Have trouble concentrating or making day-to-day decisions?	○	○	○	○	○
c.	Have a poor appetite or overeat?	○	○	○	○	○
d.	Have problems falling asleep, staying asleep, waking up too early, or sleeping too much?	○	○	○	○	○
e.	Talk or move more slowly than usual?	○	○	○	○	○
f.	Feel tired out, low in energy, or easily fatigued?	○	○	○	○	○
g.	Feel so low that it either caused distress or interfered with your activities at home, work, school, or in your social life?	○	○	○	○	○

CKPT.E3.

1. (FIVE OR MORE RESPONSES IN (E1a OR E1b), E1c, E1d, E2a, E2b, E2c, E2d, E2e, E2f = AT LEAST "SOME OF THE TIME") AND [(E1a OR E1b = AT LEAST "MOST OF THE TIME") OR (E1c = AT LEAST "MOST OF THE TIME")], GO TO E3

2. ALL OTHERS GO TO E6

E3. **About how old were you the <u>very first time</u> you (E1a = AT LEAST "MOST OF THE TIME": felt sad or depressed/E1b = AT LEAST "MOST OF THE TIME": felt discouraged/ALL OTHERS: took little interest or pleasure in things) and had some of the other problems you just mentioned that lasted at least 2 weeks?**

[DROPDOWN LIST] 4 or younger, 5, …, 35, 36 or older

E4. **About how many different years in your life did you have problems like these that lasted at least 2 weeks?**

[DROPDOWN LIST] 1, 2, …, 35, 36 or more

E5. **About how many months in the <u>past 12</u> did you have problems like these?**

[DROPDOWN LIST] 0, 1, …, 11, 12

E6. **In the <u>past 30 days</u>, how often did you have each of the following experiences?**

		All or almost all the time	Most of the time	Some of the time	A little of the time	None of the time
a.	Feel sad or depressed?	○	○	○	○	○
b.	Feel discouraged about how things were going in your life?	○	○	○	○	○
c.	Take little or no interest or pleasure in things?	○	○	○	○	○
d.	Feel down on yourself, no good, or worthless?	○	○	○	○	○

E7. **(B5d = "YES": Earlier in the survey you reported having a history of anxiety. Think about the times lasting 1 month or longer in your life when you had the strongest feelings of this sort. During those times, how often did you have each of the following experiences?/ALL OTHERS: Virtually everyone has times in their life when they feel worried or anxious. Think about the times lasting 1 month or longer in your life when you had the strongest feelings of this sort. During those times, how often did you have each of the following experiences?** *(If you are one of the few people that never had such times, mark "none of the time" to all the following questions.)***)**

		All or almost all the time	Most of the time	Some of the time	A little of the time	None of the time
a.	Feel worried or anxious?	○	○	○	○	○
b.	Worry about a number of different things in your life, such as your work, family, health, or finances?	○	○	○	○	○
c.	Feel more worried than other people in your same situation?	○	○	○	○	○
d.	Worry excessively or too much?	○	○	○	○	○

CKPT.E8.
1. E7a = AT LEAST "MOST OF THE TIME" AND E7b = AT LEAST "MOST OF THE TIME," GO TO E8
2. E7a = "NONE OF THE TIME" AND E7b = "NONE OF THE TIME" AND E7c = "NONE OF THE TIME" AND E7d = "NONE OF THE TIME," GO TO E14

3. ALL OTHERS GO TO E13

E8. During those times, how often did you have each of the following experiences?

		All or almost all the time	Most of the time	Some of the time	A little of the time	None of the time
a.	Have trouble controlling your worry?	○	○	○	○	○
b.	Feel restless, keyed up, or on edge?	○	○	○	○	○
c.	Feel tired out, low in energy, or easily fatigued?	○	○	○	○	○
d.	Have difficulty concentrating or your mind going blank?	○	○	○	○	○
e.	Feel irritated, annoyed, or grouchy?	○	○	○	○	○
f.	Have muscle aches or tension?	○	○	○	○	○
g.	Have difficulty falling or staying asleep or have restless, unsatisfying sleep?	○	○	○	○	○
h.	Feel so upset that it either caused distress or interfered with your activities at home, work, school, or in your social life?	○	○	○	○	○

CKPT.E9.

1. (E7a = AT LEAST "MOST OF THE TIME") AND (E7b = AT LEAST "MOST OF THE TIME") AND (THREE OR MORE RESPONSES IN E8b, E8c, E8d, E8e, E8f, E8g = AT LEAST "SOME OF THE TIME"), GO TO E9
2. ALL OTHERS GO TO E13

E9. How long during those times did your worry and anxiety usually last?

- ○ Less than 1 month
- ○ 1-2 months
- ○ 3-5 months
- ○ 6-7 months
- ○ 8-12 months

E10. About how old were you the <u>very first time</u> you felt worried, anxious, and had some of the other problems you just mentioned that lasted at least 1 month?

[DROPDOWN LIST] 4 or younger, 5, …, 35, 36 or older

E11. About how many different years in your life did you have problems like these that lasted at least 1 month?

[DROPDOWN LIST] 1, 2, …, 35, 36 or more

E12. About how many months in the <u>past 12</u> did you have problems like these?

[DROPDOWN LIST] 0, 1, …, 11, 12

E13. In the <u>past 30 days</u>, how often did you have each of the following experiences?

		All or almost all the time	Most of the time	Some of the time	A little of the time	None of the time
a.	Feel worried or anxious?	○	○	○	○	○
b.	Worry about a number of different things in your life, such as your work, family, health, or finances?	○	○	○	○	○
c.	Feel more worried than other people in your same situation?	○	○	○	○	○
d.	Worry excessively or too much?	○	○	○	○	○

E14. (B5c = "YES": Earlier in the survey you reported having a history of panic attacks. About how many panic attacks did you ever have in your life?/ALL OTHERS: The next question is about panic attacks, also sometimes called anxiety attacks. These are sudden, strong feelings of fear or anxiety that reach their peak within a few minutes and are usually accompanied by physical reactions like racing heart, sweating, shortness of breath, feeling faint, or feeling sick to your stomach. People who have panic attacks sometimes feel like they might lose control, go crazy, or suddenly die. With this definition in mind, about how many panic attacks did you ever have in your life?) *(You can use any number between 0 and 999 to answer.)*

_____ NUMBER OF PANIC ATTACKS

CKPT.E15.
1. E14 ≥ 1 AND ≠ MISSING, GO TO E15
2. ALL OTHERS GO TO CKPT.E23

E15. Which of the following problems do you usually have during these attacks? *(Check all that apply.)*

- ☐ A pounding or racing heart
- ☐ Sweating
- ☐ Trembling or shaking
- ☐ Shortness of breath
- ☐ Feeling like you might throw up
- ☐ Chest pain or discomfort
- ☐ Feelings of choking
- ☐ Feeling dizzy, light-headed, or faint
- ☐ Chills or heat sensations
- ☐ Numbness or tingling
- ☐ Fear of losing control or going crazy
- ☐ Fear of dying
- ☐ Feeling like things around you were unreal or like a dream
- ☐ Feeling like you were "not really there," like you were watching a movie of yourself

CKPT.E16.
3. (E14 = 1 OR 2) AND (FOUR OR MORE RESPONSES CHECKED IN E15), GO TO E22
4. FOUR OR MORE RESPONSES CHECKED IN E15, GO TO E16
5. ALL OTHERS GO TO CKPT.E23

E16. Attacks like these sometimes happen without provocation ("out of the blue") and other times occur in situations where a person has a strong fear (e.g., a fear of heights or of snakes) or is in real danger (e.g., a motor vehicle accident). When did your attacks occur?

○ <u>All</u> of your attacks occurred <u>without</u> provocation ("out of the blue")

- ◯ Some of your attacks occurred "out of the blue" and others in situations where you had a strong fear or were in real danger
- ◯ All of your attacks occurred in situations where you had a strong fear or were in real danger

CKPT.E17.

1. E16 = "ALL OF YOUR ATTACKS OCCURRED WITHOUT PROVOCATION," GO TO E18
2. E16 = "SOME OF YOUR ATTACKS OCCURRED 'OUT OF THE BLUE' AND OTHERS IN SITUATIONS WHERE YOU HAD A STRONG FEAR OR WERE IN REAL DANGER," GO TO E17
3. E16 = "ALL OF YOUR ATTACKS OCCURRED IN SITUATIONS WHERE YOU HAD A STRONG FEAR OR WERE IN REAL DANGER," GO TO E22
4. ALL OTHERS GO TO CKPT.E23

E17. About how many "out of the blue" attacks did you ever have in your life? *(You can use any number between 1 and 999 to answer.)*

______ NUMBER OF ATTACKS

CKPT.E18.

1. E16 = "SOME OF YOUR ATTACKS OCCURRED 'OUT OF THE BLUE' AND OTHERS IN SITUATIONS WHERE YOU HAD A STRONG FEAR OR WERE IN REAL DANGER" AND E17 = 0-2, GO TO E22
2. ALL OTHERS GO TO E18

E18. (E16 = "SOME OF YOUR ATTACKS OCCURRED "OUT OF THE BLUE" AND OTHERS IN SITUATIONS WHERE YOU HAD A STRONG FEAR OR WERE IN REAL DANGER": During the time when these "out of the blue" attacks were most severe or frequent, how often did you…/ALL OTHERS: During the time when these attacks were most severe or frequent, how often did you…)

		All or almost all the time	Most of the time	Some of the time	A little of the time	None of the time
a.	worry about having another attack?	◯	◯	◯	◯	◯
b.	avoid going certain places or doing certain things because you might have another attack or change your everyday activities to avoid having another attack?	◯	◯	◯	◯	◯

CKPT.E19.

1. E18a OR E18b = AT LEAST "A LITTLE OF THE TIME," GO TO E19
2. ALL OTHERS GO TO E22

E19. (E16 = "SOME OF YOUR ATTACKS OCCURRED "OUT OF THE BLUE" AND OTHERS IN SITUATIONS WHERE YOU HAD A STRONG FEAR OR WERE IN REAL DANGER": During the time when these "out of the blue" attacks were most severe or frequent, how long did you worry about having another attack or change your behaviour because of these attacks?/ALL OTHERS: During the time when these attacks were most severe or frequent, how long did you worry about having another attack or change your behaviour because of these attacks?)

- ◯ Less than 1 month
- ◯ 1-2 months
- ◯ 3-5 months
- ◯ 6-7 months
- ◯ 8-12 months

E20. **(E16 = "SOME OF YOUR ATTACKS OCCURRED "OUT OF THE BLUE" AND OTHERS IN SITUATIONS WHERE YOU HAD A STRONG FEAR OR WERE IN REAL DANGER": About how old were you the <u>very first time</u> you had an "out of the blue" panic attack?/ALL OTHERS: About how old were you the <u>very first time</u> you had a panic attack?)**

[DROPDOWN LIST] 4 or younger, 5, …, 35, 36 or older

E21. **(E16 = "SOME OF YOUR ATTACKS OCCURRED "OUT OF THE BLUE" AND OTHERS IN SITUATIONS WHERE YOU HAD A STRONG FEAR OR WERE IN REAL DANGER": About how many different years in your life did you have at least one of these "out of the blue" attacks?/ALL OTHERS: About how many different years in your life did you have at least one of these attacks?)**

[DROPDOWN LIST] 1, 2, …, 35, 36 or more

NOTE: In the original World Mental Health College Student Survey, Question E22 asked: "About how many days out of 365 in the past year did you have one or more of these attacks?" This question was edited in the current version of the survey to instead ask about the number of months in the past year. These need to be converted to the same metric when doing analysis.

E22. **(E16 = "SOME OF YOUR ATTACKS OCCURRED "OUT OF THE BLUE" AND OTHERS IN SITUATIONS WHERE YOU HAD A STRONG FEAR OR WERE IN REAL DANGER": About how many months in the <u>past 12</u> did you have one or more "out of the blue" panic attacks?/ALL OTHERS: About how many months in the <u>past 12</u> did you have one or more panic attacks?)**

[DROPDOWN LIST] 0, 1, …, 11, 12

CKPT.E22a.
1. E22 = 1-12, GO TO E22a
2. ALL OTHERS GO TO CKPT.E23

E22a. **(E16 = "SOME OF YOUR ATTACKS OCCURRED "OUT OF THE BLUE" AND OTHERS IN SITUATIONS WHERE YOU HAD A STRONG FEAR OR WERE IN REAL DANGER": About how many days in the <u>past 30</u> did you have one or more "out of the blue" panic attacks?/ALL OTHERS: About how many days in the <u>past 30</u> did you have one or more panic attacks)?**

[DROPDOWN LIST] 0, 1, …, 29, 30

CKPT.E23.
1. B5b = "YES," GO TO E24
2. ALL OTHERS GO TO E23

E23. **The next question is about whether you ever in your life had an episode lasting several days or longer when your mood was much higher than usual and you were much more excitable or hyper than usual. We don't mean an expected reaction to something that was fun or exciting, but an episode when you were really different from usual in terms of your reactions to things. Please carefully read the following description of these episodes:**

I. **These episodes usually last between several days and several weeks. During these episodes, people feel one or more of the following experiences:**

- **Much happier, in a much better mood, or much more excitable than usual**
- **Much more self-confident or optimistic than usual**
- **Or much more irritable, grumpy, or quick-tempered than usual**

II. **During these episodes, people often are:**

- **Much more energetic, active, or hyper than usual**
- **Much more talkative, open, or outgoing than usual**
- **Or much more productive or engaged than usual in work, school, or other activities**

III. People sometimes do things that are unusual for them during these episodes, such as one or more of the following:

- **Spending too much money on things they don't need**
- **Getting into relationships they would not usually get into**
- **Doing other things they would normally be too worried or embarrassed to do**

With this definition in mind, do you think you ever in your life had an episode of this sort?

◯ Yes
◯ No

CKPT.E24.

1. E23 = "YES," GO TO E24
2. ALL OTHERS GO TO E34

E24. (B5b = "YES": Earlier in the survey you reported having a history of manic-depression, mania, or bipolar disorder. Think about a typical intense episode lasting several days or longer when your mood was much higher than usual and you were much more excitable or hyper than usual./ALL OTHERS: Think about a typical intense episode of this sort lasting several days or longer.) How much of the time during that episode…

		All or almost all the time	Most of the time	Some of the time	A little of the time	None of the time
a.	were you in a much better mood, much happier, or much more excitable than usual?	◯	◯	◯	◯	◯
b.	were you much more irritable or quick to take offense than usual?	◯	◯	◯	◯	
c.	were you a lot more self-confident or optimistic than usual or believed you could do anything?	◯	◯	◯	◯	◯

CKPT.E25.

1. E24a OR E24b = AT LEAST "SOME OF THE TIME," GO TO E25
2. ALL OTHERS GO TO E34

E25. How much of the time during that episode were you…

		All or almost all the time	Most of the time	Some of the time	A little of the time	None of the time
a.	much more active or energetic than usual?	◯	◯	◯	◯	◯
b.	much more hyper or wound up than usual?	◯	◯	◯	◯	◯
c.	much more engaged, busy, or productive than usual at school or work?	◯	◯	◯	◯	◯
d.	much more sociable or outgoing than usual?	◯	◯	◯	◯	◯
e.	much more involved than usual in thinking about or doing something sexual?	◯	◯	◯	◯	◯

E26. How much of the time during that episode…

	All or almost all the time	Most of the time	Some of the time	A little of the time	None of the time
a. did you sleep much less than usual and still did not get tired or sleepy?	○	○	○	○	○
b. did you talk so much that other people couldn't get their say?	○	○	○	○	○
c. did thoughts race through your mind so fast you could hardly keep track of them?	○	○	○	○	○
d. did you have a hard time concentrating on what you were doing?	○	○	○	○	○
e. did you make bad decisions that could have caused problems for you?	○	○	○	○	○

CKPT.E27.

1. (E24a OR E24b = AT LEAST "SOME OF THE TIME") AND (ONE OR MORE RESPONSES IN E25a-e SERIES = AT LEAST "SOME OF THE TIME") AND (TWO OR MORE RESPONSES IN E24c, (E25a OR E25b OR E25c OR E25d OR E25e), E26a, E26b, E26c, E26d, E26e = AT LEAST "SOME OF THE TIME"), GO TO E27
2. ALL OTHERS GO TO E34

E27. About how old were you the <u>very first time</u> you had an episode of this sort that lasted several days or longer?

[DROPDOWN LIST] 4 or younger, 5, …, 35, 36 or older

E28. About how many different years in your life did you have an episode of this sort that lasted several days or longer?

[DROPDOWN LIST] 1, 2, …, 35, 36 or more

E29. What was the longest number of days <u>in a row</u> you ever had an episode of this sort?

_____ NUMBER OF DAYS

E30. How much did episodes of this sort ever interfere with your activities at home, work, school, or in your social life?

○ Extremely
○ A lot
○ Some
○ A little
○ Not at all

E31. How often during episodes of this sort did anyone notice or comment that you were much more energetic, wound up, productive, or outgoing than usual?

○ Often
○ Sometimes
○ Rarely
○ Never

E32. **Were you ever hospitalized for one of these episodes?**

○ Yes
○ No

NOTE: In the original World Mental Health College Student Survey, Question E33 asked: "About how many days out of 365 in the past year did you have an episode of this sort?" This question was edited in the current version of the survey to instead ask about the number of months in the past year. These need to be converted to the same metric when doing analysis.

E33. **About how many months in the <u>past 12</u> did you have an episode of this sort?**

[DROPDOWN LIST] 0, 1, …, 11, 12

CKPT.E33a.

1. E33 = 1-11, GO TO E33a
2. ALL OTHERS GO TO E34

E33a. **Did you have an episode of this sort at any time in the <u>past 30 days</u>?**

○ Yes
○ No

E34. **Did you ever in your life have repeated attacks of anger when all of a sudden you lost control and either broke or smashed something, hit or tried to hurt someone, or threatened someone?**

○ Yes
○ No

CKPT.E35.

1. E34 = "YES," GO TO E35
2. ALL OTHERS GO TO E36

E35. **About how many months in the <u>past 12</u> did you have one or more of these anger attacks?**

[DROPDOWN LIST] 0, 1, …, 11, 12

CKPT.E35a.

1. E35 = 1-11, GO TO E35a
2. ALL OTHERS GO TO E36

E35a. **Did you have one of these attacks at any time in the <u>past 30 days</u>?**

○ Yes
○ No

E36. **Did you ever in your life have times lasting 1 month or longer after an extremely stressful experience when you had frequent upsetting memories or dreams, felt jumpy, felt emotionally distant or depressed, and had trouble sleeping or concentrating?**

○ Yes
○ No

CKPT.E37.
1. E36 = "YES," GO TO E37
2. ALL OTHERS GO TO E38

E37. About how many months in the <u>past 12</u> did you have reactions like these to any extremely stressful experience?

[DROPDOWN LIST] 0, 1, …, 11, 12

CKPT.E37a.
1. E37 = 1-11, GO TO E37a
2. ALL OTHERS GO TO E38

E37a. Did you have reactions like these at any time in the <u>past 30 days</u>?

- ○ Yes
- ○ No

E38. Did you ever have a time in your life when you were much more fearful, anxious, or shy than other people about being in social situations (e.g., meeting new people, attending a party, eating in public, talking to people in authority, speaking up in class)?

- ○ Yes
- ○ No

CKPT.E39.
1. E38 = "YES," GO TO E39
2. ALL OTHERS GO TO E42

E39. How often do you try to avoid these social situations?

- ○ All or almost all the time
- ○ Most of the time
- ○ Some of the time
- ○ A little of the time
- ○ None of the time

E40. How much does your fear, anxiety, or avoidance of social situations ever interfere with your life?

- ○ Extremely
- ○ A lot
- ○ Some
- ○ A little
- ○ Not at all

E41. About how many months in the <u>past 12</u> were you much more fearful, anxious, or shy than other people about being in social situations?

[DROPDOWN LIST] 0, 1, …, 11, 12

CKPT.E41a.
1. E41 = 1-11, GO TO E41a
2. ALL OTHERS GO TO E42

E41a. Did you have this fear or anxiety at any time in the past 30 days?

- ◯ Yes
- ◯ No

E42. Did you ever in your life have times lasting 3 months or longer when you had eating binges at least once a week; that is, your eating was out of control and you ate a very large amount of food over a short period of time (2 hours or less)?

- ◯ Yes
- ◯ No

CKPT.E43.
1. E42 = "YES," GO TO E43
2. ALL OTHERS GO TO E44

E43. About how many months in the past 12 did you have eating binges?

[DROPDOWN LIST] 0, 1, …, 11, 12

CKPT.E43a.
1. E43 = 1-11, GO TO E43a
2. ALL OTHERS GO TO E44

E43a. Did you have eating binges at any time in the past 30 days?

- ◯ Yes
- ◯ No

E44. Did you ever in your life have times lasting 3 months or longer when you made yourself vomit or took laxatives or did other things to avoid gaining weight after binge eating?

- ◯ Yes
- ◯ No

CKPT.E45.
1. E44 = "YES," GO TO E45
2. ALL OTHERS GO TO E46

E45. About how many months in the past 12 did you make yourself vomit or take laxatives or do other things to avoid gaining weight after binge eating?

[DROPDOWN LIST] 0, 1, …, 11, 12

CKPT.E45a.
1. E45 = 1-11, GO TO E45a
2. ALL OTHERS GO TO E46

E45a. Did you do these things at any time in the past 30 days?

- ○ Yes
- ○ No

E46. Did you ever have an unusual experience like seeing things that other people couldn't see or hearing things that other people couldn't hear, not including when you were half-asleep or drinking alcohol or taking drugs?

- ○ Yes
- ○ No

E47. Did you ever have strange thoughts like believing your mind was being controlled by outside forces, that someone or something was sending you special signs (like through the TV), or that someone or something was plotting to harm you?

- ○ Yes
- ○ No

END OF SECTION

SECTION F: ALCOHOL AND DRUGS

F1. The following questions have to do with drinking alcohol.

How often do you have a drink containing alcohol?

- ○ 4 or more times a week
- ○ 2-3 times a week
- ○ 2-4 times a month
- ○ Monthly or less
- ○ Never

CKPT.F2.
1. F1 = "NEVER," GO TO F10.1
2. ALL OTHERS GO TO F2

F2. How many drinks containing alcohol do you have on a typical day when you are drinking?

- ○ 10 or more
- ○ 7 to 9
- ○ 5 or 6
- ○ 4
- ○ 3
- ○ 2
- ○ 1
- ○ I never drink alcohol [PROGRAMMER NOTE: RESPONSE OPTION ONLY DISPLAYS IF F1 = MISSING]

CKPT.F3.
1. F1 = MISSING AND F2 = MISSING OR "I NEVER DRINK ALCOHOL", GO TO F10.1
2. ALL OTHERS GO TO F2a

F2a. How many drinks containing alcohol do you have during a typical week?

- ○ More than 15
- ○ Between 11 and 15
- ○ 10 or less

F2b. How often do you have (A2 = "MALE": 4 or more/ALL OTHERS: 3 or more) drinks on one occasion?

- ○ Daily or almost daily
- ○ Weekly
- ○ Monthly
- ○ Less than monthly
- ○ Never

CKPT.F3.
1. F2b = "NEVER," GO TO F4
2. ALL OTHERS GO TO F3

F3. How often do you have (A2 = "MALE": 5 or more/ALL OTHERS: 4 or more) drinks on one occasion?

- ○ Daily or almost daily

○ Weekly
○ Monthly
○ Less than monthly
○ Never

CKPT.F3a.
1. F3 = "NEVER," GO TO F4
2. ALL OTHERS GO TO F3a

F3a. How often do you have 6 or more drinks on one occasion?

○ Daily or almost daily
○ Weekly
○ Monthly
○ Less than monthly
○ Never

F4. How often in the past 12 months have you…

		Daily or almost daily	Weekly	Monthly	Less than monthly	Never
a.	found that you were not able to stop drinking once you had started?	○	○	○	○	○
b.	failed to do what was normally expected of you because of drinking?	○	○	○	○	○
c.	needed a first drink in the morning to get yourself going after a heavy drinking session?	○	○	○	○	○
d.	had a feeling of guilt or remorse after drinking?	○	○	○	○	○
e.	been unable to remember what happened the night before because of your drinking?	○	○	○	○	○

F5. Have you or someone else been injured because of your drinking?

○ Yes, in the past 12 months
○ Yes, but not in the past 12 months
○ No

F6. Has a relative, friend, doctor, or other health care worker been concerned about your drinking or suggested you cut down?

○ Yes, in the past 12 months
○ Yes, but not in the past 12 months
○ No

CKPT.F7.
1. F3= AT LEAST "LESS THAN MONTHLY," GO TO F7
2. ONE OR MORE RESPONSES IN F4a-e SERIES = AT LEAST "LESS THAN MONTHLY," GO TO F7

3. F5 OR F6 = "YES, IN THE PAST 12 MONTHS" OR "YES, BUT NOT IN THE PAST 12 MONTHS," GO TO F7
4. ALL OTHERS GO TO F10.1

F7. [(F3 = AT LEAST "LESS THAN MONTHLY") AND (ALL RESPONSES IN F4a-e SERIES = "NEVER" OR MISSING) AND (F5 = "NO" OR MISSING) AND (F6 = "NO" OR MISSING) AND (A2 = "MALE"): You reported having 5 or more drinks on at least one occasion in your life./(F3 = AT LEAST "LESS THAN MONTHLY") AND (ALL RESPONSES IN F4a-e SERIES = "NEVER" OR MISSING) AND (F5 = "NO" OR MISSING) AND (F6 = "NO" OR MISSING) AND (A2 ≠ "MALE"): You reported having 4 or more drinks on at least one occasion in your life./(F3 = "NEVER" OR MISSING) AND (F4a = AT LEAST "LESS THAN MONTHLY") AND (F4b AND F4c AND F4d AND F4e = "NEVER" OR MISSING) AND (F5 = "NO" OR MISSING) AND (F6 = "NO" OR MISSING): You reported that you were not able to stop drinking once you had started./(F3 = "NEVER" OR MISSING) AND (F4b = AT LEAST "LESS THAN MONTHLY") AND (F4a AND F4c AND F4d AND F4e = "NEVER" OR MISSING) AND (F5 = "NO" OR MISSING) AND (F6 = "NO" OR MISSING): You reported that you failed to do what was expected of you because of drinking./(F3 = "NEVER" OR MISSING) AND (F4c = AT LEAST "LESS THAN "MONTHLY") AND (F4a AND F4b AND F4d AND F4e = "NEVER" OR MISSING) AND (F5 = "NO" OR MISSING) AND (F6 = "NO" OR MISSING): You reported that you needed a drink in the morning to get yourself going./(F3 = "NEVER" OR MISSING) AND (F4d = AT LEAST "LESS THAN MONTHLY") AND (F4a AND F4b AND F4c AND F4e = "NEVER" OR MISSING) AND (F5 = "NO" OR MISSING) AND (F6 = "NO" OR MISSING): You reported that you felt guilt or remorse after drinking./(F3 = "NEVER" OR MISSING) AND (F4e = AT LEAST "LESS THAN MONTHLY") AND (F4a AND F4b AND F4c AND F4d = "NEVER" OR MISSING) AND (F5 = "NO" OR MISSING) AND (F6 = "NO" OR MISSING): You reported that you were unable to remember what happened the night before because of drinking./(F3 = "NEVER" OR MISSING) AND (ALL RESPONSES IN F4a-e SERIES = "NEVER" OR MISSING) AND (F5 = "YES, IN THE PAST 12 MONTHS" OR "YES, BUT NOT IN THE PAST 12 MONTHS") AND (F6 = "NO" OR MISSING): You reported that you or someone else has been injured because of your drinking./(F3 = "NEVER" OR MISSING) AND (ALL RESPONSES IN F4a-e SERIES = "NEVER" OR MISSING) AND (F5 = "NO" OR MISSING) AND (F6 = "YES, IN THE PAST 12 MONTHS" OR "YES, BUT NOT IN THE PAST 12 MONTHS"): You reported that others have been concerned about your drinking./ALL OTHERS: You reported several experiences associated with drinking alcohol (related to frequency of drinks, quantity of drinks, and/or behaviours that were concerning to you or others).] About how old were you the <u>very first time</u> you had [(TWO OR MORE RESPONSES IN F3, F4a-e SERIES = AT LEAST "LESS THAN MONTHLY") OR (ONE RESPONSE IN F3, F4a-e SERIES = AT LEAST "LESS THAN MONTHLY" AND F5 OR F6 = "YES, IN THE PAST 12 MONTHS" OR "YES, BUT NOT IN THE PAST 12 MONTHS") OR (F5 AND F6 = "YES, IN THE PAST 12 MONTHS" OR "YES, BUT NOT IN THE PAST 12 MONTHS"): any of these experiences/ALL OTHERS: this experience]?

[DROPDOWN LIST] 4 or younger, 5, …, 35, 36 or older

F8. About how many different years in your life did you have [(TWO OR MORE RESPONSES IN F3, F4a-e SERIES = AT LEAST "LESS THAN MONTHLY") OR (ONE RESPONSE IN F3, F4a-e SERIES = AT LEAST "LESS THAN MONTHLY" AND F5 OR F6 = "YES, IN THE PAST 12 MONTHS" OR "YES, BUT NOT IN THE PAST 12 MONTHS") OR (F5 AND F6 = "YES, IN THE PAST 12 MONTHS" OR "YES, BUT NOT IN THE PAST 12 MONTHS"): any of these experiences/ALL OTHERS: this experience] associated with drinking alcohol? *(If less than 1 full year, choose "1.")*

[DROPDOWN LIST] 1, 2, …, 35, 36 or more

F9. About how many months in the <u>past 12</u> did you have [(TWO OR MORE RESPONSES IN F3, F4a-e SERIES = AT LEAST "LESS THAN MONTHLY") OR (ONE RESPONSE IN F3, F4a-e SERIES = AT LEAST "LESS THAN MONTHLY" AND F5 OR F6 = "YES, IN THE PAST 12 MONTHS" OR "YES, BUT NOT IN THE PAST 12 MONTHS") OR (F5 AND F6 = "YES, IN THE PAST 12 MONTHS" OR "YES, BUT NOT IN THE PAST 12 MONTHS"): any of these experiences/ALL OTHERS: this experience] associated with drinking alcohol?

[DROPDOWN LIST] 0, 1, …, 11, 12

F9a. Did you have [(TWO OR MORE RESPONSES IN F3, F4a-e SERIES = AT LEAST "LESS THAN MONTHLY") OR (ONE RESPONSE IN F3, F4a-e SERIES = AT LEAST "LESS THAN MONTHLY" AND F5 OR F6 = "YES, IN THE PAST 12 MONTHS" OR "YES, BUT NOT IN THE PAST 12 MONTHS") OR (F5

AND F6 = "YES, IN THE PAST 12 MONTHS" OR "YES, BUT NOT IN THE PAST 12 MONTHS"): any of these experiences/ALL OTHERS: this experience] associated with drinking alcohol at any time in the past 30 days?

- ○ Yes
- ○ No

F10.1. The following questions ask about your experience with various drugs

Think of the times in your life when you took each of the following medications most often for medical reasons, with a doctor's prescription and taken as prescribed. During those times, how often did you take each medication?

		Every or nearly every day	3-4 days a week	1-2 days a week	1-3 days a month	Less than once a month	Never
a.	Cannabis **with** a doctor's prescription and **taken as prescribed**	○	○	○	○	○	○
b.	Any prescription stimulant (e.g., amphetamine, methylphenidate, modafinil) **with** a doctor's prescription and **taken as prescribed**	○	○	○	○	○	○
c.	Any pharmaceutical opioid (e.g., oxycodone, morphine, hydromorphone, meperidine, fentanyl patches, codeine or codeine-containing products such as Tylenol #1, #2, or #3) **with** a doctor's prescription and **taken as prescribed**	○	○	○	○	○	○
d.	Any other prescription drug, such as anxiolytics, benzodiazepines [e.g., lorazepam (Activan), alprazolam (Xanax), diazepam (valium), clonazepam], or muscle relaxants (e.g., methocarbamol, baclofen) **with** a doctor's prescription and **taken as prescribed**	○	○	○	○	○	○

F10. Now think of the times in your life when you used each of the following substances most often without a doctor's prescription or in larger doses than prescribed. During those times, how often did you use each substance?

		Every or nearly every day	3-4 days a week	1-2 days a week	1-3 days a month	Less than once a month	Never
a.	Any prescription stimulant (e.g., amphetamine, methylphenidate, modafinil) either **without** a doctor's prescription or in **larger doses than prescribed** to get high, buzzed, or numbed out	○	○	○	○	○	○

b.	Any pharmaceutical opioid (e.g., oxycodone, morphine, hydromorphone, meperidine, fentanyl patches, codeine or codeine-containing products such as Tylenol #1, #2, or #3) either **without** a doctor's prescription or in **larger doses than prescribed** to get high, buzzed, or numbed out	○	○	○	○	○	○
c.	Any other prescription drug, such as anxiolytics, benzodiazepines [e.g., lorazepam (Ativan), alprazolam (Xanax), diazepam (valium), clonazepam], or muscle relaxants (e.g., methocarbamol, baclofen) either **without** a doctor's prescription or in **larger doses than prescribed** to get high, buzzed, or numbed out	○	○	○	○	○	○
d.	Cannabis (e.g., marijuana, pot, grass, hash) for non-medical reasons	○	○	○	○	○	○
e.	Any street stimulant (e.g., cocaine, crack, meth, crystal meth) or any other stimulant obtained "on the street"	○	○	○	○	○	○
f.	Any street opioid (e.g., heroin, fentanyl) or any other opioid obtained "on the street"	○	○	○	○	○	○
g.	Any other street drugs (e.g., LSD, ecstasy, PCP, mushrooms)	○	○	○	○	○	○

CKPT.F11.

1. ONE OR MORE RESPONSES IN F10a-g SERIES = AT LEAST "LESS THAN ONCE A MONTH," GO TO F11
2. ALL OTHERS GO TO CKPT.F18

F11. Think of the 1 year in your life when your drug use (street drugs, drugs used <u>without</u> a doctor's prescription, or in <u>larger doses than prescribed</u>) interfered most with your life. During <u>that year</u>, how often did you have each of the following experiences?

		Every or nearly every day	3-4 days a week	1-2 days a week	1-3 days a month	Less than once a month	Never
a.	How often did your drug use or being under the influence interfere with your responsibilities at home, work, or school?	○	○	○	○	○	○
b.	How often did you continue to use even when it caused arguments or other serious problems with your family or friends?	○	○	○	○	○	○
c.	How often were you under the influence in situations where you could get hurt?	○	○	○	○	○	○
d.	How often did you greatly reduce important activities with family, friends, or at work because of your drug use?	○	○	○	○	○	○

e.	How often did you either use more or spend more time using than you intended when you started?	○	○	○	○	○	○

F12. During <u>that year</u>, how often did you have each of these other experiences?

		Every or nearly every day	3-4 days a week	1-2 days a week	1-3 days a month	Less than once a month	Never
a.	How often did you spend a great deal of time obtaining, using, or recovering from drug use?	○	○	○	○	○	○
b.	How often did you have a strong desire or craving to use?	○	○	○	○	○	○
c.	How often did you feel the need to cut down or stop your drug use?	○	○	○	○	○	○
d.	How often did you continue to use even when it either caused or worsened a physical or emotional health problem?	○	○	○	○	○	○
e.	You developed tolerance; that is, either the same amount no longer had the same effect or you needed to use a lot more to get the same effect?	○	○	○	○	○	○
f.	You experienced withdrawal symptoms like trouble sleeping, emotional problems, restlessness, sweating, or nausea when you tried to cut down your use or you continued to use in order to avoid having withdrawal symptoms?	○	○	○	○	○	○

CKPT.F13.
1. TWO OR MORE RESPONSES IN F11a-e SERIES, F12a-f SERIES = AT LEAST "LESS THAN ONCE A MONTH," GO TO F13
2. ALL OTHERS GO TO CKPT.F18

F13. You reported several experiences associated with using street drugs, drugs without a doctor's prescription, or in larger doses than prescribed. About how old were you the <u>very first time</u> you had any of these experiences?

[DROPDOWN LIST] 4 or younger, 5, …, 35, 36 or older

F14. About how many different years in your life did you have any of these experiences?

[DROPDOWN LIST] 1, 2, …, 35, 36 or more

F15. About how many months in the <u>past 12</u> did you have any of these experiences?

[DROPDOWN LIST] 0, 1, …, 11, 12

CKPT.F15a.
1. F15 = 1-11, GO TO F15a
2. ALL OTHERS GO TO CKPT.F18

F15a. Did you have any of these experiences in the <u>past 30 days</u>?

- ◯ Yes
- ◯ No

CKPT.F18.

1. (TWO OR MORE RESPONSES IN F10a-g SERIES = AT LEAST "LESS THAN ONCE A MONTH") AND (TWO OR MORE RESPONSES IN F11a-e SERIES, F12a-f SERIES = AT LEAST "LESS THAN ONCE A MONTH"), GO TO F18
2. F10.1a OR F10d = AT LEAST "LESS THAN ONCE A MONTH," GO TO F19
3. ONE OR MORE RESPONSES IN F10.1a-d AND/OR F10a-g SERIES = AT LEAST "LESS THAN ONCE A MONTH," GO TO F22
4. F1 = AT LEAST "MONTHLY OR LESS" OR F2 = 1 OR MORE, GO TO F22
5. ALL OTHERS GO TO NEXT SECTION

F18. Earlier you mentioned that you have used more than one drug. Which drug or drugs caused most of the experiences you described above? *(Check all that apply.)*

- ☐ Cannabis for non-medical reasons
- ☐ Stimulants (including prescription medications taken without a doctor's prescription and street drugs)
- ☐ Opioids (including prescription medications taken without a doctor's prescription and street drugs)
- ☐ Any other prescription drug taken without a doctor's prescription
- ☐ Any other street drugs

CKPT.F19.

1. F10.1a OR F10d = AT LEAST "LESS THAN ONCE A MONTH," GO TO F19
2. ALL OTHERS GO TO F22

F19. You mentioned that you use or used cannabis. Are you aware of the existence of two main components of cannabis, namely tetrahydrocannabinol (THC) and cannabidiol (CBD), and of their different properties?

- ◯ <u>Yes,</u> I'm aware of their existence <u>and</u> of their different properties
- ◯ <u>Yes</u>, I'm aware of their existence <u>but not</u> of their different properties
- ◯ No, I'm not aware of their existence

CKPT.F20.

1. F19 ≠ "NO, I'M NOT AWARE OF THEIR EXISTENCE," GO TO F20
2. ALL OTHERS GO TO F21

F20. If able to choose, do you choose higher tetrahydrocannabinol (THC) or higher cannabidiol (CBD) content?

- ◯ Higher THC
- ◯ Higher CBD
- ◯ I don't care or don't know for sure

F21. Which of the following statements is true about your use of cannabis? *(Check all that apply.)*

- ☐ I use/have used synthetic cannabis (spice, K2, etc.)
- ☐ I mostly <u>smoke</u> cannabis (i.e., a joint)

- ☐ I mostly <u>smoke</u> cannabis in combination with tobacco
- ☐ When I smoke cannabis, I inhale deeply and/or hold my breath to increase the effect
- ☐ I mostly vape cannabis flowers and/or leaves, or take edibles
- ☐ I mostly vape cannabis extracts, concentrates, oils, or other processed forms of cannabis
- ☐ I use only once a week, on weekends, or less

F22. With respect to driving or operating any machinery…

- ◯ I absolutely <u>never</u> drive or operate machinery after drinking alcohol, taking or using any of the medications or drugs listed previously (or I do not ever drive or operate machinery)
- ◯ I only <u>rarely</u> drive or operate heavy machinery after drinking alcohol, taking or using any of the medications or drugs listed previously
- ◯ I <u>sometimes</u> drive or operate heavy machinery after drinking alcohol, taking or using any of the medications or drugs listed previously

CKPT.F23.

1. A2 = "FEMALE" AND F1 = AT LEAST "MONTHLY OR LESS" OR F2 = 1 OR MORE, GO TO F23
2. A2 = "FEMALE" AND ONE OR MORE RESPONSES IN F10a-g SERIES = AT LEAST "LESS THAN ONCE A MONTH," GO TO F23
3. ALL OTHERS GO TO CKPT.F24

F23. Have you used alcohol or drugs while pregnant or breastfeeding? For this question, only consider street drugs or other drugs used <u>without</u> a doctor's prescription or in <u>larger doses than prescribed</u>.

- ◯ I absolutely <u>never</u> use/used alcohol or drugs while pregnant or breastfeeding
- ◯ I <u>sometimes</u> use/used alcohol or drugs while pregnant or breastfeeding
- ◯ I only <u>rarely</u> use/used alcohol or drugs while pregnant or breastfeeding
- ◯ I've never been pregnant or breastfeeding

CKPT.F24.

1. F10.1c OR F10b OR F10f = AT LEAST "LESS THAN ONCE A MONTH," GO TO F24
2. ALL OTHERS GO TO NEXT SECTION

F24. You mentioned that you use or used opioids. In which circumstance did you first use opioids?

- ◯ They were prescribed to me for cancer-related pain
- ◯ They were prescribed to me for non-cancer pain (e.g., a sports lesion, back pain, etc.)
- ◯ They were prescribed to a family member, roommate or close acquaintance who shared them with me, or from whom I took some
- ◯ I was offered them by someone who used them to get high, relax, party, or other non-medically indicated reason
- ◯ I bought them on the street or took them from someone without their knowledge
- ◯ Other *(Please briefly describe)*

____

CKPT.F25.

1. F24= "THEY WERE PRESCRIBED TO ME FOR NON-CANCER PAIN," GO TO F25
2. ALL OTHERS GO TO NEXT SECTION

F25. What kind of non-cancer pain did/do you have?

- ○ Of the muscles, joints, or soft tissue, such as resulting from sports or work injuries, fibromyalgia, chronic back or neck pain, etc.
- ○ Neuropathic, such as due to diabetes, nerve injury, or infections
- ○ Visceral pain, such as due to lesions or disease of internal organs
- ○ Post-surgical pain
- ○ Other

F26. When you first consulted for such pain, did you receive non-opioid medication (such as acetaminophen or non-steroidal anti-inflammatories including ibuprofen, diclofenac, celecoxib, etc., or antidepressants) and/or non-pharmacologic interventions (such as exercise, mindfulness, yoga, Tai-Chi, massage or manipulation, or acupuncture)?

- ○ Yes, I received **optimal trials** of non-opioid medication plus non-pharmacologic interventions (i.e., adequately monitored interventions lasting one month or longer) **before** resorting to opioids
- ○ Yes, I **briefly** tried non-opioids and non-pharmacologic interventions (i.e., for days to weeks), but **quickly** concluded they wouldn't work for me
- ○ No, I was prescribed opioids directly

F27. When you first started opioid therapy, do you recall the first drug you used and in which daily dose?

- ○ Morphine, 50mg or less
- ○ Morphine, more than 50mg
- ○ Morphine, don't remember the dose
- ○ Codeine, 300mg or less
- ○ Codeine, more than 300mg
- ○ Codeine, don't remember the dose
- ○ Oxycodone, 30mg or less
- ○ Oxycodone, more than 30mg
- ○ Oxycodone, don't remember the dose
- ○ Hydromorphone, 10mg or less
- ○ Hydromorphone, more than 10mg
- ○ Hydromorphone, don't remember the dose
- ○ Tramadol, 300mg or less
- ○ Tramadol, more than 300mg
- ○ Tramadol, don't remember the dose
- ○ Fentanyl patch, 25mcg/h or less
- ○ Fentanyl patch, more than 25mcg/h
- ○ Fentanyl, don't remember the dose
- ○ Other, don't remember

F28. During the course of your treatment with opioids, did your doctor maintain the initial drug and dose, switch to a different opioid, or significantly increase the dose?

- ○ My doctor maintained the initial dose and achieved pain control with it
- ○ My doctor maintained the initial dose despite <u>not achieving</u> pain control with it
- ○ My doctor switched me to one or more different opioids because of persistent pain
- ○ My doctor significantly increased my dose because of persistent pain
- ○ Don't know

F29. Did your doctor attempt to taper down (i.e., gradually decrease) or discontinue your opioid treatment?

- ○ Yes, we successfully tapered down and discontinued the opioid. I never used opioids again.
- ○ Yes, we successfully tapered down and discontinued the opioid. I had to start again later because of recurring pain.
- ○ Yes, we successfully tapered down to the lowest effective dose, which I'm still taking.
- ○ Yes, we tried to taper down the dose but were not able due to recurring pain or abstinence, so I'm still taking it.
- ○ Yes, we tried to taper down the dose but were not able due to recurring pain or abstinence, so I was referred to a specialized multidisciplinary program.
- ○ No, my doctor didn't try to taper down, I'm still taking the same dose
- ○ No, my doctor didn't try to taper down. I eventually stopped using on my own

F30. Is your pain under control now?

- ○ Yes, completely
- ○ Yes, partially
- ○ Not at all

F31. Are you using opioids now?

- ○ Yes, street opioids
- ○ Yes, pharmaceutical opioids, as prescribed
- ○ Yes, pharmaceutical opioids, without prescription or in larger amounts than prescribed
- ○ No

END OF SECTION

SECTION G: SELF-HARM

The following sections include questions about sensitive topics, such as self-harm and traumatic experiences. Your responses are completely anonymous, and will improve our understanding of the mental health needs of students. If these questions are stressful or you prefer not to answer, you may skip them.

G1. Did you ever in your life wish you were dead or would go to sleep and never wake up?

- ○ Yes
- ○ No

G2. Did you ever in your life have thoughts of killing yourself?

- ○ Yes
- ○ No

CKPT.G3.

1. G1 OR G2 = "YES," GO TO G3
2. ALL OTHERS GO TO G16

G3. About how old were you the <u>very first time</u> you (G1 = "YES" AND G2 = "NO" OR MISSING: wished you were dead or would go to sleep and never wake up/G1 = "NO" OR MISSING AND G2 = "YES": had thoughts of killing yourself/ALL OTHERS: either wished you were dead, wished you would go to sleep and never wake up, or had thoughts of killing yourself)?

[DROPDOWN LIST] 4 or younger, 5, …, 35, 36 or older

G4. About how many different years in your life did you (G1 = "YES" AND G2 = "NO" OR MISSING: wish you were dead or would go to sleep and never wake up/G1 = "NO" OR MISSING AND G2 = "YES": have thoughts of killing yourself/ALL OTHERS: either wish you were dead, wish you would go to sleep and never wake up, or have thoughts of killing yourself)? *(If less than 1 full year, choose "1.")*

[DROPDOWN LIST] 1, 2, …, 35, 36 or more

G5. About how many months in the <u>past 12</u> did you (G1 = "YES" AND G2 = "NO" OR MISSING: wish you were dead or would go to sleep and never wake up/G1 = "NO" OR MISSING AND G2 = "YES": have thoughts of killing yourself/ALL OTHERS: either wish you were dead, wish you would go to sleep and never wake up, or have thoughts of killing yourself)?

[DROPDOWN LIST] 0, 1, …, 11, 12

CKPT.G6.

1. G5 = 1-12, GO TO G6
2. ALL OTHERS GO TO G7

G6. In the <u>past 30 days</u>, how often did you (G1 = "YES" AND G2 = "NO" OR MISSING: wish you were dead or would go to sleep and never wake up/G1 = "NO" OR MISSING AND G2 = "YES": have thoughts of killing yourself/ALL OTHERS: either wish you were dead, wish you would go to sleep and never wake up, or have thoughts of killing yourself)?

- ○ All or almost all of the time
- ○ Most of the time

- ◯ Some of the time
- ◯ A little of the time
- ◯ None of the time

G7. Did you ever think about how you might kill yourself (e.g., taking pills, shooting yourself) or work out a plan of how to kill yourself?

- ◯ Yes
- ◯ No

CKPT.G8.

1. G7 = "NO" OR MISSING, GO TO G12
2. ALL OTHERS GO TO G8

G8. About how old were you the <u>very first time</u> you thought about how you might kill yourself or worked out a plan of how to kill yourself?

[DROPDOWN LIST] 4 or younger, 5, …, 35, 36 or older

G9. About how many different years in your life did you think about how you might kill yourself or work out a plan of how to kill yourself? *(If less than 1 full year, choose "1.")*

[DROPDOWN LIST] 1, 2, …, 35, 36 or more

G10. About how many months in the <u>past 12</u> did you think about how you might kill yourself or work out a plan of how to kill yourself?

[DROPDOWN LIST] 0, 1, …, 11, 12

G11. How likely do you think it is that you will act on this plan in the <u>next 12 months</u>?

- ◯ Very likely
- ◯ Somewhat likely
- ◯ Not very likely
- ◯ Not at all likely

G12. Think of the one week in your life when you thought most about (G1 = "YES" AND G2 = "NO" OR MISSING: wanting to be dead/G1 = "NO" OR MISSING AND G2 = "YES": killing yourself/ALL OTHERS: wanting to be dead or about killing yourself). How many days during that worst week did you have those thoughts?

[DROPDOWN LIST] 1, …, 6, 7

G13. (G12 ≥ 2 AND ≠ MISSING: How long during that worst week did those thoughts usually last on the days that you had them?/ALL OTHERS: How long during that worst week did those thoughts last on the day that you had them?)

- ◯ Just a few seconds or minutes
- ◯ Less than 1 hour
- ◯ 1-4 hours
- ◯ 5-8 hours
- ◯ 9 or more hours

G14. During that worst week, how easy was it for you to control those thoughts or push them out of your mind when you wanted to?

- ○ Easy
- ○ A little difficult
- ○ Somewhat difficult
- ○ Very difficult
- ○ Impossible; unable to control the thoughts

CKPT.G15.

1. G1 OR G2 = "YES," GO TO G15
2. ALL OTHERS GO TO G16

G15. People who think about wanting to die sometimes do dangerous things as a way to tempt fate (e.g., take a lot of drugs, drive too fast, volunteer for dangerous missions, or act recklessly). How often in your life did you ever do dangerous things like that to tempt fate?

- ○ Very often
- ○ Often
- ○ Sometimes
- ○ Rarely
- ○ Never

G16. Have you ever made a suicide attempt (i.e., purposefully hurt yourself with at least some intent to die)?

- ○ Yes
- ○ No

CKPT.G17.

1. G16 = "NO" OR MISSING, GO TO G20
2. ALL OTHERS GO TO G17

G17. About how old were you the <u>very first time</u> you made a suicide attempt?

[DROPDOWN LIST] 4 or younger, 5, …, 35, 36 or older

G18. How many different suicide attempts have you ever made?

_____ NUMBER OF SUICIDE ATTEMPTS IN LIFETIME

G19. How many different suicide attempts have you made in the <u>past 12 months</u>?

_____ NUMBER OF SUICIDE ATTEMPTS IN PAST YEAR

G20. Did you ever do something to hurt yourself on purpose, without wanting to die (e.g., cutting yourself, hitting yourself, or burning yourself)?

- ○ Yes
- ○ No

CKPT.G21.

1. G20 = "NO" OR MISSING, GO TO NEXT SECTION
2. ALL OTHERS GO TO G21

G21. About how old were you the <u>very first time</u> you did something to hurt yourself on purpose, without wanting to die?

[DROPDOWN LIST] 4 or younger, 5, …, 35, 36 or older

G22. About how many times in your life did you do something to hurt yourself on purpose, without wanting to die?

[DROPDOWN LIST]

- ○ 1-2 times
- ○ 3-5 times
- ○ 6-10 times
- ○ 11-20 times
- ○ 21-30 times
- ○ 31-50 times
- ○ 51-100 times
- ○ 101 or more times

G23. How many times in the <u>past 12 months</u> did you do something to hurt yourself on purpose, without wanting to die?

[DROPDOWN LIST]

- ○ 0 times
- ○ 1-2 times
- ○ 3-4 times
- ○ 5-10 times
- ○ 11-20 times
- ○ 21-30 times
- ○ 31-50 times
- ○ 51-100 times
- ○ 101 or more times

END OF SECTION

SECTION H: SEEKING TREATMENT

H1. Did you ever in your life receive psychological counselling or medication for an emotional or substance use problem?

		Yes	No
a.	Psychological counselling	◯	◯
b.	Medication	◯	◯

CKPT.H2.

1. H1a = "NO" OR MISSING AND H1b = "NO" OR MISSING, GO TO H6
2. ALL OTHERS GO TO H2

H2. How old were you the <u>very first time</u> you received psychological counselling or medication for an emotional or substance use problem?

[DROPDOWN LIST] 4 or younger, 5, …, 35, 36 or older

H3. About how many months in the <u>past 12</u> did you receive psychological counselling or medication?

[DROPDOWN LIST] 0, 1, …, 11, 12

CKPT.H4.

1. H3 = 1-12, GO TO H5
2. ALL OTHERS GO TO H4

H4. How old were you the <u>most recent time</u> you received psychological counselling or medication for an emotional or substance use problem?

[DROPDOWN LIST] 4 or younger, 5, …, 35, 36 or older

H5. Are you still in treatment or have you stopped?

◯ Still in treatment
◯ Stopped

CKPT.H6.

1. H5 = "STILL IN TREATMENT," GO TO H9
2. ALL OTHERS GO TO H6

H6. How would you rate your readiness or willingness to change any emotional or substance use problems you are experiencing <u>at this time</u>?

◯ I do not have a problem that I need to change
◯ I have a problem, but I am not yet sure I want to take action to change it
◯ I have a problem and I intend to address it
◯ I have a problem and I already am working actively to change it
◯ I had a problem but I have addressed it and things are better now

CKPT.H7.

1. H3 = 1-12, GO TO H9
2. ALL OTHERS GO TO H7

H7. Was there ever a time in the <u>past 12 months</u> when you felt that you might need psychological counselling or medication for any emotional or substance use problems?

○ Yes
○ No ⟶ GO TO H9

[PROGRAMMER: SKIP MISSING WITH "NO"]

H8. How important were each of the following reasons for why you did NOT seek help for your problem(s)?

		Very important	Important	Moderately important	Of little importance	Unimportant
a.	You were not sure if available treatments were very effective	○	○	○	○	○
b.	You wanted to handle the problem on your own	○	○	○	○	○
c.	You were too embarrassed	○	○	○	○	○
d.	You talked to friends or relatives instead	○	○	○	○	○
e.	It costs too much money	○	○	○	○	○
f.	You were unsure of where to go or who to see	○	○	○	○	○
g.	You had problems with time, transportation, or scheduling	○	○	○	○	○
h.	You were afraid it might harm your school or professional career	○	○	○	○	○
i.	You worried that people would treat you differently if they knew you were in treatment	○	○	○	○	○
j.	Some other reason *(Briefly describe in the text box below)*	○	○	○	○	○

H9. In the past, have you ever looked for information, help, or treatment for any emotional or substance use problem on the internet, through apps or online tools?

○ Yes
○ No

H10. Now or in the future, if you wanted to receive support managing stress and responsibilities, help dealing with emotional or substance use issues, or psychotherapy, how likely would you be to try an e-mental health resource, such as an app or an online tool?

○ Very likely
○ Somewhat likely

- ○ Not very likely
- ○ Not at all likely

END OF SECTION

SECTION I: CHILDHOOD BACKGROUND

I1. Is your mother still alive or is she deceased?

- ◯ Alive
- ◯ Deceased

CKPT.I1a.

1. I1 = "ALIVE" OR MISSING, GO TO I1c
2. ALL OTHERS GO TO I1b

I1b. How much education did she have?

- ◯ None
- ◯ Elementary school
- ◯ Secondary school
- ◯ Some post-secondary education
- ◯ University graduate
- ◯ Doctoral degree
- ◯ Don't know

GO TO I2

I1c. How much education does she have?

- ◯ None
- ◯ Elementary school
- ◯ Secondary school
- ◯ Some post-secondary education
- ◯ University graduate
- ◯ Doctoral degree
- ◯ Don't know

I2. Is your father still alive or is he deceased?

- ◯ Alive
- ◯ Deceased

CKPT.I2a.

1. I2 = "ALIVE" OR MISSING, GO TO I2c
2. ALL OTHERS GO TO I2b

I2b. How much education did he have?

- ◯ None
- ◯ Elementary school
- ◯ Secondary school
- ◯ Some post-secondary education
- ◯ University graduate

○ Doctoral degree
○ Don’t know

GO TO CKPT.I3a

I2c. How much education does he have?

○ None
○ Elementary school
○ Secondary school
○ Some post-secondary education
○ University graduate
○ Doctoral degree
○ Don’t know

CKPT.I3a.

1. I1 = “DECEASED” AND I2 = “ALIVE” OR MISSING, GO TO I3a
2. I1 = “ALIVE” OR MISSING AND I2 = “DECEASED,” GO TO I3b
3. I1 = “DECEASED” AND I2 = “DECEASED,” GO TO I3c
4. I1 = “ALIVE” OR MISSING AND I2 = “ALIVE” OR MISSING, GO TO I3d

I3a. At the time your mother died, were your parents married to each other, separated, or divorced?

○ Married
○ Separated
○ Divorced
○ They were never married

GO TO I4

I3b. At the time your father died, were your parents married to each other, separated, or divorced?

○ Married
○ Separated
○ Divorced
○ They were never married

GO TO I4

I3c. At the time the first of your parents died, were they married to each other, separated, or divorced?

○ Married
○ Separated
○ Divorced
○ They were never married

GO TO I4

I3d. Are your parents married to each other, separated, or divorced?

○ Married

○ Separated
○ Divorced
○ They were never married

I4. How much of the time did you have each of the following experiences up through age 17?

		Very often	Often	Sometimes	Rarely	Never
a.	One of your parents (or the people who raised you) had a serious emotional or mental health problem	○	○	○	○	○
b.	One of your parents (or the people who raised you) had a serious alcohol or drug problem	○	○	○	○	○
c.	One of your parents (or the people who raised you) attempted suicide or died by suicide	○	○	○	○	○
d.	One of your parents (or the people who raised you) was involved in criminal activities	○	○	○	○	○
e.	Your parents (or the people who raised you) hit each other or were violent to each other	○	○	○	○	○
f.	Someone in your family hit you so hard that it left bruises or marks	○	○	○	○	○

I5. And how much of the time did you have each of these experiences up through age 17?

		Very often	Often	Sometimes	Rarely	Never
a.	You were physically abused at home	○	○	○	○	○
b.	Someone in your family repeatedly said hurtful or insulting things to you	○	○	○	○	○
c.	You were emotionally abused at home	○	○	○	○	○
d.	Someone in your family made you feel special or important	○	○	○	○	○
e.	Someone in your family touched you or made you touch them in a sexual way	○	○	○	○	○

I6. And how much of the time did you have each of these experiences up through age 17?

		Very often	Often	Sometimes	Rarely	Never
a.	You were sexually abused at home	○	○	○	○	○
b.	You were seriously neglected at home (e.g., nobody took care of you or protected you or made sure you had the things you needed)	○	○	○	○	○
c.	You had to do chores too hard or dangerous for someone your age	○	○	○	○	○
d.	You felt loved and cared for by your family/at home	○	○	○	○	○

e.	People in your family looked out for you and took care of you	○	○	○	○	○
f.	You felt emotionally close to your family members	○	○	○	○	○

I7. Were you physically or sexually abused outside your home or family up through age 17?

- ○ No
- ○ Yes, physically
- ○ Yes, sexually
- ○ Yes, both

END OF SECTION

SECTION J: RECENT EXPERIENCES

J1. Did you have any of the following stressful experiences in the past 12 months?

		Yes	No
a.	A life-threatening illness or injury of a very close friend or family member	○	○
b.	The death of a close friend or family member	○	○
c.	A break-up with a romantic partner	○	○
d.	You discovered that a romantic partner cheated on you	○	○
e.	A serious betrayal by someone else close to you	○	○
f.	Serious ongoing arguments or break-ups with some other close friend or family member	○	○

J2. Did you have any of the following stressful experiences in the past 12 months?

		Yes	No
a.	You were involved in a life-threatening accident	○	○
b.	You were bullied	○	○
c.	You were physically assaulted (e.g., mugged)	○	○
d.	You were sexually assaulted or raped	○	○
e.	You were sexually harassed (e.g., someone made inappropriate sexual remarks about you or your body)	○	○
f.	You had trouble with the police	○	○
g.	You spent time in jail	○	○
h.	You had a serious legal problem	○	○
i.	Any other very stressful event *(Briefly describe in the text box below)*	○	○

J3. How much stress do you currently have in each of the following areas of your life?

		Very severe	Severe	Moderate	Mild	None
a.	Your financial situation	○	○	○	○	○
b.	Your health	○	○	○	○	○
c.	Your academic performance	○	○	○	○	○
d.	Your love life	○	○	○	○	○
e.	Your relationships with your family	○	○	○	○	○
f.	Problems getting along with people at work or school	○	○	○	○	○
g.	The health of your loved ones	○	○	○	○	○
h.	Other problems experienced by your loved ones	○	○	○	○	○
i.	Your life overall	○	○	○	○	○

J4. The next few questions are about your social life. How often do you talk on the phone, text, or chat online with friends or relatives?

- ◯ Almost every day
- ◯ A few times a week
- ◯ A few times a month
- ◯ Once a month
- ◯ Less than once a month
- ◯ Never

J5. How often do you get together in person with friends for social or recreational activities?

- ◯ Almost every day
- ◯ A few times a week
- ◯ A few times a month
- ◯ Once a month
- ◯ Less than once a month
- ◯ Never

CKPT.J6.

1. J4 OR J5 = AT LEAST "ONCE A MONTH," GO TO J6
2. ALL OTHERS GO TO J7

J6. About how many friends and relatives do you talk to on the phone, text, chat with online, or get together with at least once a month?

______ NUMBER OF FRIENDS AND RELATIVES

J7. How often do you attend meetings of religious, fraternal, social, or recreational groups you belong to?

- ◯ Almost every day
- ◯ A few times a week
- ◯ A few times a month
- ◯ Once a month
- ◯ Less than once a month
- ◯ Never

J8. How much could you rely on people in your personal life for support and comfort if you had a serious personal problem?

- ◯ A lot
- ◯ Some
- ◯ A little → GO TO J10
- ◯ Not at all → GO TO J10

[PROGRAMMER: SKIP MISSING WITH "A LOT"]

J9. How many people could you rely on for support and comfort if you had a serious personal problem?

_______ NUMBER OF PEOPLE

J10. How many people in your personal life would you feel comfortable opening up to and confiding in without feeling embarrassed if you had a serious personal problem? *(If none, enter "0".)*

_______ NUMBER OF PEOPLE

J11. How often do the people in your personal life make too many demands on you?

- ◯ Often
- ◯ Sometimes
- ◯ Rarely
- ◯ Never

J12. How often do the people in your personal life argue with you or say things that make you feel bad about yourself?

- ◯ Often
- ◯ Sometimes
- ◯ Rarely
- ◯ Never

END OF SECTION

SECTION K: SEXUALITY

K1. What is your sexual orientation?

- ◯ Heterosexual or straight
- ◯ Gay or lesbian
- ◯ Bisexual
- ◯ Asexual
- ◯ Not sure
- ◯ Other *(Briefly describe in the text box below)*

____

K2. Which of the following best describes your feelings of sexual attraction to women?

- ◯ Very sexually attracted
- ◯ A good deal sexually attracted
- ◯ Somewhat sexually attracted
- ◯ A little sexually attracted
- ◯ Not at all sexually attracted

K3. Which of the following best describes your feelings of sexual attraction to men?

- ◯ Very sexually attracted
- ◯ A good deal sexually attracted
- ◯ Somewhat sexually attracted
- ◯ A little sexually attracted
- ◯ Not at all sexually attracted

K4. In the <u>past 5 years</u>, who have you had sex with? *(We use the word "sex" to mean any kind of sexual contact, not just sexual intercourse, but also, for example, oral sex or masturbation.)*

- ◯ Men only
- ◯ Women only
- ◯ Non-binary only
- ◯ More than one of the above
- ◯ I have not had sex

K5. What is your marital status?

- ◯ Married ⟶ GO TO CKPT.K7
- ◯ Separated
- ◯ Divorced
- ◯ Widowed
- ◯ Never married

[PROGRAMMER: SKIP MISSING WITH "NEVER MARRIED"]

K6. Which of the following best describes your current relationship situation?

- ◯ Living with someone in a marriage-like relationship or engaged to be married
- ◯ Steadily dating one person, but not engaged
- ◯ Dating multiple people in committed relationships
- ◯ Dating one or more people, but not in a steady relationship
- ◯ Not currently dating

CKPT.K7.

1. K4 ≠ "I HAVE NOT HAD SEX," GO TO K7
2. ALL OTHERS END SURVEY

K7. Have you ever used Viagra (sildenafil), Cialis (tadalafil) or other similar drugs to improve your sexual function?

- ◯ Yes, every time I have sex
- ◯ Yes, most of the times I have sex
- ◯ Yes, some of the times I have sex
- ◯ Yes, once or a few times
- ◯ Never

END OF SURVEY
