# Supplementary material for: The World Mental Health International College Student Survey in Canada: Protocol for a Mental Health and Substance Use Trend Study
Source: JMIR Res Protoc. 2022 Jul 29;11(7):e35168. doi: 10.2196/35168 (PMC9377443; doi:10.2196/35168)
Supplement: Multimedia Appendix 2 [file resprot_v11i7e35168_app2.pdf]

Multimedia Appendix 2. French survey instrument.

DSM-5 Santé Mentale Mondiale

Sondage d'Étudiants Universitaire

Point de Comparaison

FORME ABRÉGÉE

VERSION 2

Le 24 septembre, 2019

Ronald C. Kessler, Ph.D.
McNeil Family Professor of Health Care Policy
L'École de médecine de Harvard

Révisé et adapté pour les étudiants Canadien par Daniel Vigo

Le 9 Juillet, 2021

____

Commentaires à: RC Kessler, Department of Health Care Policy, Harvard Medical School, 180 Longwood Avenue, Boston, MA, USA 02115; kessler@hcp.med.harvard.edu; 617-432-3587 (Voix); 617-432-3588 (Fax)

ORDRE DES SECTIONS:

SECTION A: VOTRE MILIEU
SECTION B: VOTRE SANTÉ
SECTION D : ATTENTION ET CONCENTRATION
SECTION E: VOS PROBLÈMES ÉMOTIONNELS
SECTION F: L'ALCOOL ET DROGUES
SECTION G: AUTOMUTILATION
SECTION H: CHERCHER DE L'AIDE
SECTION I: VOTRE MILIEU D'ENFANCE
SECTION J: EXPÉRIENCES RÉCENTES
SECTION K: SEXUALITÉ

Remarque: **S'il vous plait NE PAS cliquer sur la flèche de retour de votre navigateur Internet car vous serez éjecté de l'enquête**. Si vous voulez revoir une question, vous pouvez utiliser le bouton bleu "Retour" qui se situe dans le sondage à plusieurs reprises. Si le bouton n'est pas là, vous ne pouvez pas retourner à la question précédente

SECTION A: VOTRE MILIEU

A1. Quel âge avez-vous?

[LISTE DÉROULANTE] 16 ans ou moins, 17, … , 35, 36 ans et plus

A2. Quel était votre sexe à la naissance?

- ○ Masculin
- ○ Féminin

A2a. Quel est votre genre actuel?

- ○ Masculin
- ○ Féminin
- ○ Non-binaire
- ○ Masculin et féminin
- ○ Autre *(Merci de préciser dans la case ci-dessous)*

____

A3. Quel est votre statut d'étudiant actuel?

- ○ Étudiant(e) universitaire, 1ère année
- ○ Étudiant(e) universitaire, 2ième année
- ○ Étudiant(e) universitaire, 3ième année
- ○ Étudiant(e) universitaire, 4ième année
- ○ Étudiant(e) universitaire, 5ième année
- ○ Étudiant(e) de troisième cycle
- ○ Étudiant(e) non-diplômé
- ○ Récemment diplômé
- ○ Autre *(Merci de préciser dans la case ci-dessous)*

____

A3a. Quel est le statut de vos cours actuellement?

- ○ Entièrement en ligne
- ○ Une combinaison en ligne et en personne
- ○ Entièrement en personne
- ○ Je ne prends pas de cours pour le moment
- ○ Autre *(Merci de préciser dans la case ci-dessous)*

____

A4. Quel est votre race, ethnie, ou héritage? *(Cochez toutes les propositions qui vous concernent)*

- ☐ Blanc
- ☐ Hispanique ou Latino
- ☐ Noir
- ☐ Premières Nations, Métis, ou Inuit
- ☐ Sud-asiatique (par exemple, 'Est-Indien,' 'Pakistanais,' 'Sri lankais', etc.)

- ☐ Sud-est asiatique (par exemple, 'Vietnamien,' 'Cambodgien,' 'Laotien,' 'Thaïlandais,' etc.)
- ☐ Asiatique Occidentale (par exemple, 'Afghan,' 'Iranien,' etc.)
- ☐ Chinois
- ☐ Philippin
- ☐ Arabe
- ☐ Coréen
- ☐ Japonais
- ☐ Autre *(Merci de préciser dans la case ci-dessous)*

____

A5. Quel est votre langue maternelle? *(Cochez toutes les propositions qui vous concernent)*

- ☐ Anglais
- ☐ Français
- ☐ Autre *(Merci de préciser dans la case ci-dessous)*

____

A6. Êtes-vous né au Canada?

- ○ Oui VA À A8
- ○ Non

[PROGRAMMEUR: PASSER MANQUANT AVEC "OUI"]

A7. Quel âge aviez-vous quand vous avez déménagé au Canada pour la première fois? *(Donnez votre meilleure estimation si vous ne vous souvenez pas de votre âge exact. Si vous aviez moins d'un an, selectionner "4 ou moins".)*

[PROGRAMMEUR NOTE: SI R SELECTIONNE UN NUMÉRO PLUS GRAND QUE L'ÂGE ACTUEL OU SI BA1 = MANQUANT, AJOUTEZ UNE BOÎTE "VEUILLEZ SELECTIONNER UN NUMÉRO QUI N'EST PAS PLUS GRAND QUE VOTRE ÂGE ACTUEL."]

[LISTE DÉROULANTE] 4 ans ou moins, 5, … , 35, 36 ans et plus

A8. Combien de vos parents sont nés au Canada?

- ○ Deux
- ○ Un
- ○ Aucun

A8a. Vivez-vous actuellement au Canada?

- ○ Oui
- ○ Non

A9. Quelle est votre situation de logement actuelle?

- ○ Avec vos parents ou autres membres de la famille
- ○ Dans votre propre maison ou appartement
- ○ Dans une résidence appartenant à l'université et opérer par l'université
- ○ Dans une maison de fraternité ou de sororité
- ○ Dans une maison partagée ou appartement partagé

○ Autre *(Merci de préciser dans la case ci-dessous)*

____

CKPT.A10a.

1. A8a = "Oui" OU MANQUANT, VA À A10a.
2. A8a = "Non", VA À A10b.

A10a. Veuillez fournir le code postal de la région où vous vivez actuellement.

____

VA À B1

A10b. Veuillez choisir la région qui décrit le mieux votre lieu de résidence actuel:

[DROPDOWN LIST] Amérique du Nord, Amérique Centrale et Caraïbes, Amérique du Sud, Europe de l'Ouest, Europe Centrale, Europe de l'Est, Asie Centrale, Asie de l'Est et Pacifique, Asie du Sud, Afrique Subsaharienne, Afrique du Nord

FIN DE SECTION

SECTION B: VOTRE SANTÉ

La section suivante concerne votre santé. Elle comprend des questions sur COVID-19 dans le but de comprendre son impact sur le bien-être de notre communauté et d'améliorer les mesures misent en place par le système de santé. Toutes les réponses sont anonymes et ne peuvent pas être retracées aux répondants. Si ces questions sont stressantes ou si vous préférez ne pas y répondre, vous pouvez les ignorer. À la fin de l'étude, vous trouverez des ressources en santé mentale à propos de COVID-19.

B1. Comment qualifieriez-vous votre santé physique en général?

- ○ Excellente
- ○ Très bonne
- ○ Bonne
- ○ Moyenne
- ○ Mauvaise

B2. Comment qualifieriez-vous votre santé mentale en général?

- ○ Excellente
- ○ Très bonne
- ○ Bonne
- ○ Moyenne
- ○ Mauvaise

B3. Au cours des <u>12 derniers mois</u>, comment votre performance à l'école a-t-elle été affectée par les problèmes suivants?

	très sévèrement	Sévèrement	Moyennement	Légèrement	Aucunement
a. Problèmes de santé physique	○	○	○	○	○
b. Problèmes de santé mentale ou émotionnelle (par exemple l'anxiété, la dépression)	○	○	○	○	○
c. Problèmes avec la consommation d'alcool ou de drogue	○	○	○	○	○

B4. Au cours des <u>12 derniers mois</u>, comment votre vie personnelle ou sociale a-t-elle été affectée par les problèmes suivants?

	très sévèrement	Sévèreme nt	Moyenne ment	Légère ment	Aucunement
a. Problèmes de santé physique	○	○	○	○	○
b. Problèmes de santé mentale ou émotionnelle (par exemple l'anxiété, la dépression)	○	○	○	○	○
c. Problèmes avec la consommation d'alcool ou de drogue	○	○	○	○	○

B5. Avez-vous déjà eu dans votre vie l'un des problèmes émotionnels suivants?

		Oui	Non
a.	Dépression	○	○
b.	Maniaco-dépression, manie, ou trouble bipolaire	○	○
c.	Crise de panique	○	○
d.	Problèmes avec l'anxiété (nervosité, soucis, peurs, compulsions, obsessions)	○	○
e.	Autre(s) problème(s) émotionnel(s) sérieux	○	○

B6. Au cours des <u>30 derniers jours</u>, à quelle fréquence avez-vous ressenti chacune des options suivantes?

		Tout le temps ou presque	La plupart du temps	Une partie du temps	De temps en temps	Pas du tout
d.	Douleur dans le dos, le cou, le bras, les jambes ou les articulations (les genoux, les hanches, etc.)	○	○	○	○	○
e.	Mal de tête	○	○	○	○	○
f.	Douleur à d'autre partie du corps	○	○	○	○	○
g.	Tension musculaire	○	○	○	○	○
h.	Vertige	○	○	○	○	○

B7 Santé Canada indique que les symptômes suivants du COVID-19 sont courants: toux, difficulté à respirer, fièvre, frissons, fatigue, courbatures, maux de tête, perte d'odorat ou de goût et symptômes gastro-intestinaux. Avez-vous eu l'un de ces symptômes depuis janvier 2020?

○ Oui, et j'ai testé positif pour COVID-19 (ou j'ai reçu un diagnostic de COVID-19 par un fournisseur de soins)
○ Oui, et j'ai testé négatif pour COVID-19
○ Oui, mais je ne suis pas allé me faire tester ou je n'ai pas pu aller me faire tester pour COVID-19
○ Non

CKPT.B7a.

1. B7 = "Non," VA À B8
2. ALL OTHERS VA À B7a

B7a. Avez-vous (B7= "Oui, et j'ai testé positif pour COVID-19 (ou j'ai reçu un diagnostic de COVID-19 par un fournisseur de soins)": été testé positif or été diagnostiqué/B7= "Oui, et j'ai testé négatif pour COVID-19": été testé négatif /ALL OTHERS: eu ces symptômes) au cours des deux dernières semaines?

○ Oui
○ Non

CKPT.B7b.

1. B7 = "Oui, mais je ne suis pas allé me faire tester ou je n'ai pas pu aller me faire tester pour COVID-19" VA À B7b
2. ALL OTHERS VA À B7c

B7b. Pensez-vous que vous aviez définitivement le COVID-19, que vous l'aviez probablement, que vous l'aviez possiblement, que vous ne l'aviez probablement pas ou que vous ne l'aviez certainement pas?

- ◯ Je l'ai eu définitivement
- ◯ Je l'ai eu probablement
- ◯ Je l'ai eu possiblement
- ◯ Je ne l'ai probablement pas eu
- ◯ Je ne l'ai définitivement pas eu

B7c. Comment vos symptômes ont-ils été traités?

- ◯ Hospitalisé et intubé (c'est-à-dire mis sous respirateur)
- ◯ Hospitalisé mais non-intubé (c'est-à-dire pas mis sous respirateur)
- ◯ Mis en quarantaine ou isolé à domicile sous les ordres d'un professionnel de la santé
- ◯ A été donné des instructions sur l'auto-traitement et a été dit d'éviter les lieux publics, mais n'a pas été dit de se placer en quarantaine ou de s'isoler
- ◯ Non traité

B8. Connaissez-vous quelqu'un qui a été testé positif pour COVID-19 ou quelqu'un qui a reçu un diagnostic de COVID-19 par un fournisseur de soins? (Si vous connaissez des personnes dans plusieurs endroits, choisissez l'option la plus proche de l'endroit où vous vivez actuellement.)

- ◯ Oui, je connais quelqu'un dans la ville où je vis
- ◯ Oui, je connais quelqu'un dans le pays où je vis, mais pas dans la ville
- ◯ Oui, je connais quelqu'un mais pas dans le pays où je vis
- ◯ Non

CKPT.B9.

3. B8 = "NO," GO TO B11
4. ALL OTHERS GO TO B9

B9. Combien de personnes que vous connaissez ont été infectées par le COVID-19?

		**Nombre de personnes**
a.	Quelqu'un vivant avec vous à l'époque	
b.	Quelqu'un de proche ne vivant pas avec vous à ce moment-là (ex : parent, enfant, frère ou sœur, partenaire, autre membre de la famille)	
c.	Quelqu'un qui travaille avec vous	
d.	Quelqu'un qui prend un cours avec vous	
e.	Un ami proche qui ne vit pas, ne travaille pas ou ne prend pas de cours avec vous à ce moment-là	
f.	Toute autre personne que vous connaissez (ex: un voisin, une connaissance)	

B10a. Ont-ils reçu un diagnostic de COVID-19 au cours des deux dernières semaines? (Si vous connaissez plus d'une personne qui a été positive au COVID-19 au cours des deux dernières semaines, pensez à la personne présentant les symptômes les plus graves.)

- ◯ Oui, et ils ont été hospitalisés pour des symptômes sévères
- ◯ Oui, et ils ont / ont eu des symptômes légers à modérés
- ◯ Oui, et ils étaient asymptomatiques
- ◯ Non

CKPT.B10b.

1. B9a = 1 OR MORE OR B9b = 1 OR MORE OR B9c = 1 OR MORE OR B9d = 1 OR MORE OR B9e = 1 OR MORE OR B9f = 1 OR MORE, GO TO B10b
2. ALL OTHERS GO TO B11

B10b. (B9a + B9b + B9c + B9d + B9e +B9f = 1: Cette personne est-elle décédée du COVID-19?/B9a + B9b + B9c + B9d + B9e + B9f = 2: Est-ce que l'une de ces personnes est décédée du COVID-19?/ALL OTHERS: Est-ce qu'une de ces personnes est décédée du COVID-19?)

◯ Oui
◯ Non

B10c. Qui est mort du COVID-19? *(Cochez toutes les propositions qui vous concernent.)*

☐ Un de vos parents
☐ Votre être cher, partenaire ou conjoint
☐ Tout autre parent proche
☐ Un ami proche
☐ Quelqu'un avec qui vous travaillez
☐ Toute autre personne que vous connaissez (par exemple, un voisin, une connaissance)

B11. Est-ce que quelqu'un dans votre résidence/maison/appartement (B9a > "0": d'autre) a des symptômes telles qu'une toux, des difficultés respiratoires, de la fièvre, des frissons, de la fatigue, des courbatures, des maux de tête, une perte d'odorat ou de goût ou des symptômes gastro-intestinaux?

◯ Oui, mais ils ont été testés négatifs pour COVID-19
◯ Oui, mais ils n'ont pas été testés ou je ne sais pas si ils ont été testés
◯ Pas que je sache

CKPT.B12.

1. A3a = "Entièrement en personne" OR "Une combinaison en ligne et en personne", GO TO B12
2. ALL OTHERS GO TO B13

B12. Est-ce que quelqu'un dans vos cours (B9d > "0": d'autre) a des symptômes telles qu'une toux, des difficultés respiratoires, de la fièvre, des frissons, de la fatigue, des courbatures, des maux de tête, une perte d'odorat ou de goût ou des symptômes gastro-intestinaux?

◯ Oui, mais ils ont été testés négatifs pour COVID-19
◯ Oui, et je ne sais pas si ils ont été testés
◯ Pas que je sache

B13. La pandémie COVID-19 a-t-elle affecté votre bien-être émotionnel?

◯ Oui, mais j'ai pu le gérer
◯ Oui, j'étais dépassé et je n'ai pu trouver de l'aide / je suis dépassé et je ne peux pas trouver de l'aide
◯ Oui, j'étais dépassé et j'ai pu trouver de l'aide / je suis dépassé et je reçois de l'aide
◯ Non

B14. Par rapport à avant la pandémie COVID-19, dans quelle mesure diriez-vous que votre vie est «normale» maintenant, 0 étant «complètement perturbée» et 100 étant «complètement normale»?

complètement perturbée								complètement normale		
**0**	**10**	**20**	**30**	**40**	**50**	**60**	**70**	**80**	**90**	**100**

B15. Pensez au mois où votre stress lié au COVID-19 a été le plus élevé. Au cours de ce mois, combien de stress l'épidémie a-t-elle causé par rapport aux problèmes suivants?

		Stress extrêmement sévère	Sévère	Modéré	Léger	Aucun stress
a.	Finances familiales (ex : perte de revenu ou de placements)	○	○	○	○	○
b.	Isolement social accru	○	○	○	○	○
c.	Difficulté à obtenir l'aide necessaire pour vos proches	○	○	○	○	○
d.	Augmentation des disputes avec votre famille ou vos amis	○	○	○	○	○
e.	Craignez que vous soyez infecté	○	○	○	○	○
f.	Craignez que vos proches soient infectés	○	○	○	○	○

B16. Quel est votre statut de vaccination?

- ○ Je suis complètement vacciné (deux doses)
- ○ J'ai eu la première des deux doses
- ○ Je vais me faire vacciner dès que possible
- ○ Je choisis de ne pas me faire vacciner
- ○ Je ne peux pas me faire vacciner en raison d'une maladie, d'une allergie ou d'un traitement

FIN DE SECTION

SECTION D: ATTENTION ET CONCENTRATION

D. La question suivante concerne les difficultés d'organisation, de concentration ou d'impulsivité. À quelle fréquence avez-vous eu chacune des difficultés suivantes au cours des 6 derniers mois ?

	Tout le temps ou presque	La pluspart du temps	De temps en temps	Rarement	Jamais
a. Vous avez évité ou retardé de commencer lorsque vous aviez une tâche qui demandait beaucoup de réflexion	○	○	○	○	○
b. Vous avez eu des problèmes pour vous souvenir de rendez-vous ou d'obligations	○	○	○	○	○
c. Vous aviez du mal à mettre les choses en ordre lorsque vous deviez faire une tâche qui demandait de l'organisation	○	○	○	○	○
d. Vous avez eu du mal à conclure les derniers détails d'un projet une fois les parties difficiles terminées	○	○	○	○	○
e. Vous vous êtes senti trop actif et obligé de faire des choses, comme si vous étiez conduit par un moteur	○	○	○	○	○
f. Vous étiez agitiez ou vous vous tortilliez avec vos mains ou vos pieds lorsque vous deviez vous asseoir pendant longtemps	○	○	○	○	○

CKPT.D2.

1. THREE OR MORE RESPONSES IN D1a-f SERIES = AT LEAST “De temps en temps,” GO TO D2
2. ALL OTHERS GO TO NEXT SECTION

D2. Quel âge aviez-vous la première fois que vous avez eu des problèmes avec l'organisation, la concentration ou l'impulsivité?

[LISTE DÉROULANTE] 4 ou moins, 5, …, 35, 36 ou plus

FIN DE SECTION

SECTION E: PROBLÈMES ÉMOTIONNELS

E1. Les questions suivantes sont à propos des difficultés émotionnelles que vous avez vécues à un moment ou autre de votre vie.

(B7a = "OUI": Plus tôt dans l'étude, vous avez indiqué avoir un historique de dépression. Repensez à une période où vous avez ressenti ces sentiments plus intensément qu'à l'habitude pendant au moins deux semaines consécutives. Pendant ces périodes difficiles, à quelle fréquence avez-vous vécu chacune des expériences suivantes? / TOUS LES AUTRES: Il est commun de vivre des moments difficiles au cours d'une vie pendant lesquelles on se sent triste, déprimé(e), ou découragé (e). Repensez à une période où vous avez ressenti ces sentiments plus intensément qu'à l'habitude pendant au moins deux semaines consécutives. Pendant ces périodes difficiles, à quelle fréquence avez-vous vécu chacune des expériences suivantes? *(Si vous êtes l'une de rares personnes à n'avoir jamais vécu de tels moments, cochez "Jamais" à toutes les questions suivantes.)*

		Tout ou presque tout le temps	La plupart du temps	De temps en temps	Rarement	Jamais
a.	Se sentir triste ou déprimé(e)?	○	○	○	○	○
b.	Se sentir découragé(e) par la manière dont les choses se passent dans votre vie?	○	○	○	○	○
c.	N'avoir peu ou pas d'intérêt pour les choses ou ressentir peu ou pas de plaisir?	○	○	○	○	○
d.	Se sentir abattu(e), bon(ne) à rien, ou sans valeur?	○	○	○	○	○

CKPT.E2.

5. (E1a OU E1b = AU MOINS "LA PLUPART DU TEMPS") OU (E1c = AU MOINS "LA PLUPART DU TEMPS"), VA À E2
6. E1a = "PAS DU TOUT" ET E1b = "PAS DU TOUT" ET E1c = "PAS DU TOUT" ET E1d = "PAS DU TOUT," VA À E7
7. TOUS LES AUTRES VA À E6

E2. Pendant ces périodes, à quelle fréquence avez-vous vécu chacune des expériences suivantes?

		Tout ou presque tout le temps	La plupart du temps	De temps en temps	Rarement	Jamais
a.	Penser souvent à la mort (soit la vôtre, celle de quelqu'un d'autre ou de la mort en générale)?	○	○	○	○	○
b.	Avoir des difficultés à se concentrer ou à prendre des décisions?	○	○	○	○	○
c.	Manger beaucoup trop ou ne pas avoir envie de manger?	○	○	○	○	○
d.	Avoir des difficultés à s'endormir, à rester endormi, se réveiller trop tôt ou dormir trop longtemps?	○	○	○	○	○
e.	Parler ou se déplacer plus lentement qu'à l'habitude?	○	○	○	○	○
f.	Se sentir fatigué€, sans énergie, ou facilement fatigué€?	○	○	○	○	○
g.	Se sentir si dépourvu et misérable que cela a perturbé vos activités à la maison, au travail, à l'école ou dans votre vie sociale?	○	○	○	○	○

CKPT.E3.
1. (CINQ RÉPONSES OU PLUS EN (E1a OU E1b), E1c, E1d, E2a, E2b, E2c, E2d, E2e, E2f = AU MOINS "UN PEU DU TEMPS") ET [(E1a OU E1b = AU MOINS "LA PLUPART DU TEMPS") OU (E1c = AU MOINS "LA PLUPART DU TEMPS")], VA À E3
2. TOUS LES AUTRES VA À E6

E3. **Quel âge aviez-vous la toute première fois que (E1a = AU MOINS "LA PLUPART DU TEMPS" : vous vous êtes senti triste ou déprimé /E1b = AU MOINS "LA PLUPART DU TEMPS" : vous vous êtes senti découragé /TOUS LES AUTRES: vous vous êtes peu d'intéressé ou avez peu apprécié les choses) et avez eu certains des autres problèmes que vous venez de mentionner ayant duré au moins 2 semaines?**

[LISTE DÉROULANTE] 4 ans ou moins, 5, …, 35, 36 ans ou plus

E4. **Au cours de votre vie, pendant environ combien d'années différentes avez-vous eu des problèmes comme ceux-ci, qui ont duré au moins 2 semaines?**

[LISTE DÉROULANTE] 1, 2, …, 35, 36 ou plus

E5. **Au cours des 12 derniers mois, pendant environ combien de mois avez-vous eu des problèmes comme ceux-ci?**

[LISTE DÉROULANTE] 0, 1, …, 11, 12

E6. **Au cours des 30 derniers jours, à quelle fréquence avez-vous vécu chacune des expériences suivantes?**

		Tout ou presque tout le temps	La plupart du temps	De temps en temps	Rarement	Jamais
a.	Se sentir triste ou déprimé(e)?	○	○	○	○	○
b.	Se sentir découragé(e) de la façon dont les choses se passent dans votre vie?	○	○	○	○	○
c.	N'avoir peu ou pas d'intérêt pour les choses ou ressentir peu ou pas de plaisir?	○	○	○	○	○
d.	Se sentir abattu(e), bon(ne) à rien, ou sans valeur?	○	○	○	○	○

E7. **(B7d = "OUI": Plus tôt dans l'étude, vous avez indiqué avoir un historique d'anxiété. Repensez à une période où vous avez ressenti ces sentiments plus intensément qu'à l'habitude pendant au moins un mois. Pendant ces périodes difficiles, à quelle fréquence avez-vous vécu chacune des expériences suivantes? / TOUS LES AUTRES: Il est commun de vivre des moments difficiles au cours d'une vie pendant lesquelles on se sent triste, déprimé(e), ou découragé (e). Repensez à une période où vous avez ressenti ces sentiments plus intensément qu'à l'habitude pendant au moins un mois. Pendant ces périodes difficiles, à quelle fréquence avez-vous vécu chacune des expériences suivantes?** *(Si vous êtes l'une de rares personnes à n'avoir jamais eu tels moments, cochez "Jamais" a toutes les questions suivantes.)*

		Tout ou presque tout du temps	La plupart du temps	De temps en temps	Rarement	Jamais
a.	Se sentir inquiet(e) ou anxieux(se)?	○	○	○	○	○
b.	S'inquiéter de plusieurs choses différentes dans votre vie, comme le travail, la famille, la santé ou l'argent?	○	○	○	○	○
c.	Se sentir plus inquiet(e) que la plupart des gens dans la même situation que vous?	○	○	○	○	○
d.	S'inquiéter excessivement ou trop?	○	○	○	○	○

CKPT.E8.

1. E7a = AU MOINS "LA PLUPART DU TEMPS" ET E7b = AU MOINS "LA PLUPART DU TEMPS," VA À E8
2. E7a = "PAS DU TOUT" ET E7b = "PAST DU TOUT" ET E7c = "PAS DU TOUT" ET E7d = "PAS DU TOUT," VA À E14
3. TOUS LES AUTRES VA À E13

E8. Pendant ces périodes, à quelle fréquence avez-vous vécu chacune des expériences suivantes?

		Tout ou presque tout du temps	La plupart du temps	De temps en temps	Rarement	Jamais
a.	Avoir des difficultés à contrôler votre inquiétude?	○	○	○	○	○
b.	Se sentir agité(e), coincé(e) ou à bout?	○	○	○	○	○
c.	Se sentir fatigué(e), sans énergie, ou facilement fatigué(e)?	○	○	○	○	○
d.	Avoir des difficultés à se concentrer ou à vous vider de vos pensées?	○	○	○	○	○
e.	Se sentir irrité(e), ennuyé(e) ou grincheux(se)?	○	○	○	○	○
f.	Avoir des douleurs musculaires ou des tensions?	○	○	○	○	○
g.	Avoir de la difficulté à s'endormir, à rester endormi(e), avoir un sommeil agité ou insatisfaisant?	○	○	○	○	○
h.	Se sentir si bouleversé€ que cela a perturbé vos activités à la maison, au travail, à l'école ou dans votre vie sociale?	○	○	○	○	○

CKPT.E9.

1. (E7a = AU MOINS "LA PLUPART DU TEMPS") ET (E7b = AU MOINS "LA PLUPART DU TEMPS") ET (TROIS OU PLUS RÉPONSES EN E8b, E8c, E8d, E8e, E8f, E8g = AU MOINS "UNE PARTIE DU TEMPS"), VA À E9
2. TOUS LES AUTRES VA À E13

E9. Durant ces périodes, pendant combien de temps duraient votre inquiétude ou votre anxiété?

○ Moins de 1 mois
○ 1-2 mois
○ 3-5 mois
○ 6-7 mois
○ 8-12 mois

E10. **Quel âge aviez-vous environ <u>la toute première fois</u> que vous vous êtes senti inquiet, anxieux, et avez eu certains des autres problèmes que vous venez de mentionner qui ont duré au moins 1 mois?**

[LISTE DÉROULANTE] 4 ans ou moins, 5, …, 35, 36 ans ou plus

E11. **Au cours de votre vie, pendant environ combien d'années différentes avez-vous eu des problèmes comme ceux-ci, qui ont duré au moins 1 mois?**

[LISTE DÉROULANTE] 1, 2, …, 35, 36 ou plus

E12. **Au cours des <u>12 derniers mois,</u> pendant environ combien de mois avez-vous eu des problèmes comme ceux-ci?**

[LISTE DÉROULANTE] 0, 1, …, 11, 12

E13. **Au cours des 30 <u>derniers jours</u>, à quelle fréquence avez-vous vécu chacune des expériences suivantes?**

		Tout ou presque tout du temps	La plupart du temps	De temps en temps	Rarement	Jamais
a.	Se sentir inquiet(e) ou anxieux(se)?	○	○	○	○	○
b.	S'inquiéter de plusieurs choses différentes dans votre vie, comme le travail, la famille, la santé ou l'argent?	○	○	○	○	○
c.	Se sentir plus inquiet(e) que la plupart des gens dans la même situation que vous?	○	○	○	○	○
d.	S'inquiéter excessivement ou trop?	○	○	○	○	○

E14. **(B7c = "OUI": Plus tôt dans l'étude, vous avez indiqué avoir un historique de crises de panique. Environ combien de crises de panique avez-vous déjà eu dans votre vie? / TOUS LES AUTRES : La prochaine question est à propos de crises de panique (crises d'angoisse). Ce sont des sensations de peur et d'anxiété soudaines et fortes, qui atteignent leur maximum après quelques minutes et sont généralement accompagnées par des réactions physiques tels que un battement de cœur rapide, de la transpiration, se sentir essouffler, se sentir faible, avoir mal au ventre, etc. Parfois, les personnes qui ont des crises de panique ont l'impression de perdre le contrôle, devenir fou, ou mourir soudainement. Avec cette définition, environ combien de crises de panique avez-vous eu dans votre vie?** *(Vous pouvez utiliser n'importe quel numéro entre 0 et 999 pour répondre.)*

_____ NUMÉRO DE CRISES DE PANIQUE

CKPT.E15.
1. E14 ≥ 1 ET ≠ MANQUANT, VA À E15
2. TOUS LES AUTRES VA À CKPT.E23

E15. **Généralement, lesquels des problèmes suivants avez-vous pendant ces attaques?** *(Cochez toutes les propositions qui vous concernent.)*

☐ Le cœur qui bat très vite ou très fort
☐ Transpiration
☐ Tremblements, secousses musculaires

☐ Le souffle coupé
☐ La nausée, avoir envie de vomir
☐ Douleur, gêne au niveau de la poitrine
☐ La sensation d'être en train de vous étouffer
☐ Sensation de vertige, de tête vide, d'étourdissement, ou d'évanouissement
☐ Des frissons ou des bouffées de chaleur
☐ Sensation d'engourdissement ou de picotement
☐ Peur de perdre le contrôle ou devenir fou
☐ Peur de mourir
☐ Avoir la sensation que ce qui vous entoure n'est pas réel ou que vous êtes en train de rêver
☐ Avoir la sensation que vous n'êtes pas vraiment ici, comme si vous étiez en train de voir un film de vous-même

CKPT.E16.

3. (E14 = 1 OU 2) ET (QUATRE OU PLUS RÉPONSES COCHEZ EN E15), VA À E22
4. QUATRE OU PLUS RÉPONSES COCHEZ EN E15, VA À E16
5. TOUS LES AUTRES VA À CKPT.E23

E16. Les attaques comme celles-ci surviennent parfois sans provocation ("à l'improviste"), et d'autres fois elles apparaissent dans des situation ou la personne éprouve une peur intense (par exemple avoir le vertige lorsque l'on est en hauteur) ou est en danger réel (par exemple, avoir un accident de véhicule motorisé). Dans votre cas, quand vos attaque sont-elles survenues ?

○ Toutes vos attaques sont survenues sans provocation ("à l'improviste")
○ Certaines attaques sont apparues "à l'improviste" et d'autres dans des situations où vous éprouviez une peur intense ou que vous étiez en réel danger.
○ Toutes vos attaques sont survenues dans des situations où vous éprouviez une peur intense ou que vous étiez en réel danger.

CKPT.E17.

1. E16 = "TOUS VOS ATTAQUES ONT EU LIEU SANS PROVOCATION," VA À E18
2. E16 = "CERTAINES DE VOS ATTAQUES ONT EU LIEU SANS PROVOCATION ET AUTRES DANS LES SITUATIONS OÙ VOUS AVEZ UNE FORTE PEUR OU ÉTIEZ EN DANGER RÉEL," VA À E17
3. E16 = "TOUS VOS ATTAQUES ONT EU LIEU DANS LES SITUATIONS OÙ VOUS AVEZ UNE FORTE PEUR OU ÉTIEZ EN DANGER RÉEL," VA À E22
4. TOUS LES AUTRES VA À CKPT.E23

E17. Au cours de votre vie, environ combien d'attaques sont survenues "à l'improviste"? *(Vous pouvez utiliser n'importe quel numéro entre 1 et 999 pour répondre.)*

______ NUMÉRO D'ATTAQUES

CKPT.E18.

1. E16 = "CERTAINES DE VOS ATTAQUES ONT EU LIEU SANS PROVOCATION ET AUTRES DANS LES SITUATIONS OÙ VOUS AVEZ UNE FORTE PEUR OU ÉTIEZ EN DANGER RÉEL" ET E17 = 0-2, VA À E22
2. TOUS LES AUTRES VA À E18

E18. (E16 = "CERTAINES DE VOS ATTAQUES ONT EU LIEU SANS PROVOCATION ET AUTRE DANS LES SITUATIONS OÙ VOUS AVEZ UNE FORTE PEUR OU ÉTIEZ EN DANGER RÉEL": Lorsque vos attaques "à l'improviste" étaient plus sévères ou plus fréquentes, à quelle fréquence avez-vous vécu les expériences suivantes? / TOUS LES AUTRES: Lorsque vos attaques étaient plus sévères ou plus fréquentes, à quelle fréquence avez-vous vécu les expériences suivantes?

		Tout ou presque tout le temps	La plupart du temps	De temps en temps	Rarement	Jamais
a.	S'inquiéter d'avoir une autre attaque?	○	○	○	○	○
b.	Éviter d'aller à certains endroits, éviter de faire certaines choses ou changer vos activités habituelles parce que vous aviez peur d'avoir une autre attaque?	○	○	○	○	○

CKPT.E19.

1. E18a OU E18b = AU MOINS "UN PEU DU TEMPS," VA À E19
2. TOUS LES AUTRES VA À E22

E19. (E16 = "CERTAINES DE VOS ATTAQUES ONT EU LIEU SANS PROVOCATION ET AUTRE DANS LES SITUATIONS OÙ VOUS AVEZ UNE FORTE PEUR OU ÉTIEZ EN DANGER RÉEL": Lorsque vos attaques "à l'improviste" étaient plus sévères ou plus fréquentes, pendant combien de mois avez-vous été inquiet d'avoir une autre attaque? / TOUS LES AUTRES : Lorsque vos attaques étaient plus sévères ou plus fréquentes, pendant combien de mois avez-vous été inquiet d'avoir une autre attaque?

- ○ Moins de 1 mois
- ○ 1-2 mois
- ○ 3-5 mois
- ○ 6-7 mois
- ○ 8-12 mois

E20. (E16 = "CERTAINES DE VOS ATTAQUES ONT EU LIEU SANS PROVOCATION ET AUTRE DANS LES SITUATIONS OÙ VOUS AVEZ UNE FORTE PEUR OU ÉTIEZ EN DANGER RÉEL": Quel âge aviez-vous environ <u>la toute première fois</u> que vous avez eu une crise de panique "à l'improviste"? / TOUS LES AUTRES: Quel âge aviez-vous environ <u>la toute première fois</u> que vous avez eu une crise de panique?)

[LISTE DÉROULANTE] 4 ans ou moins, 5, …, 35, 36 ans ou plus

E21. (E16 = "CERTAINES DE VOS ATTAQUES ONT EU LIEU SANS PROVOCATION ET AUTRE DANS LES SITUATIONS OÙ VOUS AVEZ UNE FORTE PEUR OU ÉTIEZ EN DANGER RÉEL": Au cours de votre vie, pendant environ combien d'années différentes avez-vous eu au moins une crise de panique "à l'improviste"? / TOUS LES AUTRES: Au cours de votre vie, pendant environ combien d'années différentes avez-vous eu au moins une crise de panique?)

[LISTE DÉROULANTE] 1, 2, …, 35, 36 ou plus

NOTE: In the original World Mental Health College Student Survey, Question E22 asked: "About how many days out of 365 in the past year did you have one or more of these attacks?" This question was edited in the current version of the survey to instead ask about the number of months in the past year. These need to be converted to the same metric when doing analysis.

E22. (E16 = "CERTAINES DE VOS ATTAQUES ONT EU LIEU SANS PROVOCATION ET AUTRE DANS LES SITUATIONS OÙ VOUS AVEZ UNE FORTE PEUR OU ÉTIEZ EN DANGER RÉEL": Au cours des <u>12 derniers mois,</u> pendant environ combien de mois avez-vous eu une ou plusieurs crises de panique "à l'improviste"? / TOUS LES AUTRES: Au cours des <u>12 derniers mois,</u> pendant environ combien de mois avez-vous eu une ou plusieurs crises de panique?)

[LISTE DÉROULANTE] 0, 1, …, 11, 12

CKPT.E22a.

1. E22 = 1-12, VA À E22a
2. TOUS LES AUTRES VA À CKPT.E23

E22a. (E16 = "CERTAINES DE VOS ATTAQUES ONT EU LIEU SANS PROVOCATION ET AUTRE DANS LES SITUATIONS OÙ VOUS AVEZ UNE FORTE PEUR OU ÉTIEZ EN DANGER RÉEL": Au cours des 30 <u>derniers jours</u>, pendant environ combien de jours avez-vous eu une ou plusieurs crise de panique "à l'improviste"? / TOUS LES AUTRES: Au cours des 30 <u>derniers jours</u>, pendant environ combien de jours avez-vous eu une ou plusieurs crise de panique?)

[DROPDOWN LIST] 0, 1, …, 29, 30

CKPT.E24.
1. B5b = "OUI," VA À E24
2. TOUS LES AUTRES VA À E23

E23. La question suivante consiste à savoir si au cours de votre vie vous avez eu un épisode durant plusieurs jours ou plus où votre humeur était bien plus élevée que d'habitude et que vous étiez bien plus excitable ou surexcité(e) que d'habitude. Nous ne parlons pas d'une réaction attendue à quelque chose qui était drôle ou excitant, mais d'un épisode où vous étiez vraiment différent(e) que d'habitude dans vos réactions aux choses. Veuillez lire attentivement la description suivante de ces épisodes:

I. Ces épisodes durent généralement de plusieurs jours à plusieurs semaines. Pendant ces épisodes, les personnes ressentent une ou plusieurs des expériences suivantes et sont:

- **Beaucoup plus heureuses, d'une bien meilleure humeur ou beaucoup plus enthousiastes que d'habitude**
- **Beaucoup plus confiance en elles ou beaucoup plus optimistes que d'habitude**
- **Ou bien plus irritables, grincheuses ou irascibles que d'habitude**

II. Pendant ces épisodes, les personnes:

- **Ont beaucoup plus d'énergie, sont beaucoup plus actives que d'habitude ou sont plus surexcitées que d'habitude**
- **Sont beaucoup plus bavardes, plus ouvertes et plus extraverties que d'habitude**
- **Ou sont beaucoup plus productives ou impliquées que d'habitude dans le travail, les études ou dans d'autres activités**

III. Les personnes font parfois les choses qui sont inhabituelles pour elles durant ces épisodes, comme une ou plusieurs des choses suivantes:

- **Dépenser trop d'argent dans des choses dont elles n'ont pas besoin**
- **S'engager dans des relations dans lesquelles elles ne s'impliqueraient pas normalement**
- **Faire d'autres choses qu'elles ne feraient pas normalement parce qu'elles seraient trop gênées**

Avec cette définition en tête, pensez-vous avoir déjà eu un épisode de ce type dans votre vie?

◯ Oui
◯ Non

CKPT.E25.
1. E24 = "OUI," VA À E25
2. TOUS LES AUTRES VA À E36

E24. **(B5b = "OUI": Plus tôt dans l'étude, vous avez indiqué avoir un historique de maniaco-dépression, ou de troubles bipolaires. Repensez à un épisode intense et typique qui a duré plusieurs jours et où votre humeur était beaucoup plus élevée que d'habitude et ou que vous étiez beaucoup plus excitable ou surexciter que d'habitude. / TOUS LES AUTRES: Repensez à un épisode intense et typique qui a duré plusieurs jours de cette sorte. Combien de temps pendant cet épisode…**

		Tout le temps ou presque	La plupart du temps	De temps en temps	Rarement	Jamais
a.	Étiez-vous de bien meilleure humeur, beaucoup plus heureux(se) ou plus excitable que d'habitude?	○	○	○	○	○
b.	Étiez-vous beaucoup plus irritable ou susceptible que d'habitude?	○	○	○	○	
c.	Étiez-vous beaucoup plus confiant en soi, optimiste que d'habitude, ou pensiez-vous être capable de tout?	○	○	○	○	○

CKPT.E26.
1. E25a OU E25b = AU MOINS "UNE PARTIE DU TEMPS," VA À E26
2. TOUS LES AUTRES VA À E36

E25. **Combien de temps pendant cet épisode étiez-vous…**

		Tout le temps ou presque	La plupart du temps	De temps en temps	Rarement	Jamais
a.	Beaucoup plus actif(ve) ou énergique que d'habitude?	○	○	○	○	○
b.	Beaucoup plus surexcité(e) ou tendu(e) que d'habitude?	○	○	○	○	○
c.	Beaucoup plus intéressé(e), préoccupé(e) ou productif(ve) que d'habitude dans vos études ou au travail?	○	○	○	○	○
d.	Beaucoup plus sociable ou extraverti(e) que d'habitude?	○	○	○	○	○
e.	Beaucoup plus occupé(e) par des pensées ou des comportements sexuels que d'habitude?	○	○	○	○	○

E26. **Combien de temps pendant cet épisode…**

		Tout le temps ou presque	La plupart du temps	De temps en temps	Rarement	Jamais
a.	Avez-vous dormi beaucoup moins que d'habitude sans pour autant être fatigué(e) ou avoir envie de dormir?	○	○	○	○	○
b.	Avez-vous tellement parler que les personnes autour de vous n'ont rien pu dire?	○	○	○	○	○
c.	Avez-vous eu des pensées qui allaient si vite qu'il vous était difficile d'en suivre le fil?	○	○	○	○	○
d.	Avez-vous eu des difficultés à vous concentrer sur ce que vous faisiez?	○	○	○	○	○

e.	Avez-vous pris de mauvaises décisions qui auraient pu vous causer des problèmes?	◯	◯	◯	◯	◯

CKPT.E28.

1. (E25a OU E25b = AU MOINS "UNE PARTIE DU TEMPS") ET (UNE OU PLUS DES RÉPONSES DANS LA SÉRIE E26a-e = AU MOINS "UNE PARTIE DU TEMPS") ET (DEUX OU PLUS DES RÉPONSES EN E25c, (E26a OU E26b OU E26c OU E26d OU E26e), E27a, E27b, E27c, E27d, E27e = AU MOINS "UNE PARTIE DU TEMPS"), VA À E28
2. TOUS LES AUTRES VA À E36

E27. Environ quel âge aviez-vous environ <u>la toute première fois</u> que vous avez eu un tel épisode d'excitation qui a duré plusieurs jours ou plus?

[LISTE DÉROULANTE] 4 ans ou moins, 5, …, 35, 36 ans ou plus

E28. Au cours de votre vie, pendant environ combien d'années différentes avez-vous eu un tel épisode d'excitation qui a duré plusieurs jours ou plus?

[LISTE DÉROULANTE] 1, 2, …, 35, 36 ou plus

E29. Quel a été le plus grand nombre de jours <u>successifs au cours desquels</u> vous avez eu un épisode de ce type qui a duré plusieurs jours ou plus

_____ JOUR(S) SUCCESSIF(S)

E30. A quel point ont les difficultés de ce type perturbé vos activités à la maison, au travail, à l'école ou dans votre vie personnelle?

◯ Extrêmement
◯ Beaucoup
◯ Quelque
◯ Un peu
◯ Pas du tout

E31. À quelle fréquence quelqu'un a-t-il remarqué ou commenté que vous étiez beaucoup plus énergique, énervé(e), productif(ve), ou sociable que d'habitude?

◯ Souvent
◯ Parfois
◯ Rarement
◯ Jamais

E32. Avez-vous déjà été hospitalisé(e) pour un de ces épisodes?

◯ Oui
◯ Non

NOTE: In the original World Mental Health College Student Survey, Question E34 asked: "About how many days out of 365 in the past year did you have an episode of this sort?" This question was edited in the current version of the survey to instead ask about the number of months in the past year. These need to be converted to the same metric when doing analysis.

E33. Au cours des <u>12 derniers mois,</u> pendant combien de mois avez-vous eu un épisode de ce type?

[LISTE DÉROULANTE] 0, 1, …, 11, 12

CKPT.E35.
1. E34 = 1-11, VA À E35
2. TOUS LES AUTRES VA À E36

E33a. Avez-vous eu un épisode de ce type au cours des 30 derniers jours?

◯ Oui
◯ Non

E34. Au cours de votre vie, avez-vous déjà eu des crises de colères oú vous perdez soudainement contrôle et vous avez cassé quelque chose, brisé quelque chose, frappé quelqu'un, essayé de blesser quelqu'un ou menacé quelqu'un?

◯ Oui
◯ Non

CKPT.E37.
1. E36= « OUI », VA À E37
2. TOUS LES AUTRES VA À E39

E35. Au cours des 12 derniers mois, pendant combien de mois avez-vous eu une ou plusieurs de ces crises de colères?

[LISTE DÉROULANTE] 0, 1, …, 11, 12

CKPT.E38.
1. E37= 1-11, VA À E38
2. TOUS LES AUTRES VA À E39

E35a. Avez-vous eu une de ces attaques au cours des 30 derniers jours?

◯ Yes
◯ No

E36. Au cours de votre vie, avez-vous vécu des périodes qui ont duré 1 mois ou plus après une expérience particulièrement stressante où vous aviez des souvenirs ou rêves perturbants, vous vous sentiez nerveux(se), sentiez émotionnellement distant(e) ou déprimé(e), et vous aviez du mal à vous endormir ou à vous concentrer?

◯ Oui
◯ Non

CKPT.E40.
1. E39 = "OUI," VA À E40
2. TOUS LES AUTRES VA À E42

E37. Au cours des 12 derniers mois, pendant combien de mois avez-vous eu des réactions de ce type suite à une expérience particulièrement stressante?

[LISTE DÉROULANTE] 0, 1, …, 11, 12

CKPT.E41.

1. E40 = 1-11, VA À E41
2. TOUS LES AUTRES VA À E42

E37a. Avez-vous eu des réactions de ce type au cours des <u>30 derniers jours</u>?

- ◯ Yes
- ◯ No

E38. Dans votre vie, avez-vous déjà eu un moment où vous aviez été plus craintif(ve), anxieux(se) ou timide que d'autres personnes autour de vous dans un milieu sociale (par exemple, rencontrer de nouvelles personnes, aller à une fête, manger en public, parler en classe)?

- ◯ Oui
- ◯ Non

CKPT.E43.

1. E42 = "OUI," VA À E43
2. TOUS LES AUTRES VA À E47

E39. À quelle fréquence essayez-vous d'éviter ces situations sociales?

- ◯ Tout ou presque tout le temps
- ◯ La plupart du temps
- ◯ De temps en temps
- ◯ Rarement
- ◯ Jamais

E40. À quelle fréquence est-ce que votre peur, anxiété ou votre évitement des situations sociales ont-ils interféré avec votre vie?

- ◯ Extrêmement
- ◯ Beaucoup
- ◯ Assez
- ◯ Un peu
- ◯ Pas du tout

E41. Au cours des <u>12 derniers mois, pendant combien de mois</u> étiez-vous beaucoup plus craintif(ve), anxieux(se) ou timide que d'autres personnes face aux situations sociales?

[LISTE DÉROULANTE] 0, 1, …, 11, 12

CKPT.E46.

1. E45 = 1-11, VA À E46
2. TOUS LES AUTRES VA À E47

E41a. Avez-vous eu cette peur ou cette anxiété au cours des <u>30 derniers jours</u>?

- ◯ Oui

○ Non

E42. **Au cours de votre vie, avez-vous déjà vécu des périodes qui ont duré 3 mois ou plus où vous avez mangez en excès au moins une fois par semaine; c'est-à-dire où votre alimentation était hors de contrôle et où vous avez mangé de très grandes quantités de nourriture en peu de temps (2 heures ou moins)?**

○ Oui
○ Non

CKPT.E48.

1. E47 = "OUI," VA À E48
2. TOUS LES AUTRES VA À E50

E43. **Au cours des <u>12 derniers mois,</u> pendant combien de mois avez-vous mangez en excès, tel que décrit?**

[LISTE DÉROULANTE] 0, 1, …, 11, 12

CKPT.E49.

1. E48 = 1-11, VA À E49
2. TOUS LES AUTRES VA À E50

E43a. **Avez-vous mangez en excès au cours des <u>30 derniers jours</u> tel que décrit?**

○ Yes
○ No

E44. **Au cours de votre vie, avez-vous déjà vécu des périodes qui ont duré 3 mois ou plus où vous vous êtes fait vomir ou avez pris des laxatifs ou fait d'autres choses pour éviter de prendre du poids après avoir mangé en excès?**

○ Oui
○ Non

CKPT.E51.

1. E50 = "OUI," VA À E51
2. TOUS LES AUTRES VA À E53

E45. **Au cours des 12 derniers mois, pendant combien de mois vous êtes-vous fait vomir, ou avez pris des laxatifs, ou fait d'autres choses pour éviter de prendre du poids après avoir mangé en excès?**

[LISTE DÉROULANTE] 0, 1, …, 11, 12

CKPT.E52.

1. E51 = 1-11, VA À E52
2. TOUS LES AUTRES VA À E53

E45a. **Au cours des 30 derniers jours, vous êtes-vous fait vomir, ou avez pris des laxatifs, ou fait d'autres choses pour éviter de prendre du poids après avoir mangé en excès?**

○ Oui

○ Non

E46. **Au cours de votre vie, avez-vous déjà eu des expériences inhabituelles comme voir ou entendre des choses que les autres personnes ne pouvaient pas voir ou entendre (en dehors des fois où vous étiez à moitié endormi(e) ou vous aviez consommer de l'alcool ou des drogues)?**

○ Oui
○ Non

E47. **Au cours de votre vie, avez-vous déjà eu des pensées étranges comme croire que votre esprit était contrôlé par des forces extérieures, comme penser que quelqu'un ou quelque chose vous envoyait personnellement des messages (à la télévision), ou que quelqu'un ou quelque chose complotait contre vous pour vous faire du mal?**

○ Oui
○ Non

FIN DE SECTION

SECTION F: L'ALCOOL ET LES DROGUES

F1. Les questions suivantes concernent la consommation d'alcool. À quelle fréquence consommez-vous une boisson contenant de l'alcool?

- ○ 4 fois ou plus par semaine
- ○ 2-3 fois par semaine
- ○ 2-4 fois par mois
- ○ Une fois par mois ou moins
- ○ Jamais

CKPT.F2.
1. F1 = "JAMAIS," VA À F10
2. TOUS LES AUTRES VA À F2

F2. Lors d'un jour typique où vous buvez de l'alcool, combien de boissons alcoolisées consommez-vous ?

- ○ 10 ou plus
- ○ 7 à 9
- ○ 5 ou 6
- ○ 4
- ○ 3
- ○ 2
- ○ 1
- ○ Je ne bois jamais d'alcool [PROGRAMMEUR NOTE: L'OPTION DE RÉPONSE S'AFFICHE UNIQUEMENT SI F1 = MANQUANT]

CKPT.F2a.
1. F1 = MANQUANT ET F2 = MANQUANT OU "JE NE BOIS JAMAIS D'ALCOOL", VA À F11
2. TOUS LES AUTRES VA À F2a

F2a. Pendant une semaine normale, combien de boissons alcoolisées consommez-vous?

- ○ Plus que 15
- ○ Entre 11 et 15
- ○ Moins que 10

F2b. À quelle fréquence consommez-vous (A2 = « Homme » : 4 ou plus / TOUS LES AUTRES : 3 ou plus) boissons alcoolisées lors d'une même occasion?

- ○ Tous les jours ou presque
- ○ Environ une fois par semaine
- ○ Environ une fois par mois
- ○ Moins d'une fois par mois
- ○ Jamais

CKPT.F3.
1. F2b = "NEVER," GO TO F4
2. ALL OTHERS GO TO F3

F3. À quelle fréquence consommez-vous (A2 = « Homme » : 5 ou plus / TOUS LES AUTRES : 4 ou plus) boissons alcoolisées lors d'une même occasion?

- ○ Tous les jours ou presque

- ○ Environ une fois par semaine
- ○ Environ une fois par mois
- ○ Moins d'une fois par mois
- ○ Jamais

CKPT.F3a.
1. F3 = "NEVER," GO TO F4
2. ALL OTHERS GO TO F3a

F3a. À quelle fréquence consommez-vous 6 ou plus boissons alcoolisées lors d'une même occasion?

- ○ Tous les jours ou presque
- ○ Environ une fois par semaine
- ○ Environ une fois par mois
- ○ Moins d'une fois par mois
- ○ Jamais

F4. Au cours des <u>12 derniers mois,</u> à quelle fréquence avez-vous…

		Tous les jours ou presque	Une fois par semaine	Une fois par mois	Moins d'une fois par mois	Jamais
a.	constaté que vous n'étiez pas capable d'arrêter de boire après avoir commencé?	○	○	○	○	○
b.	été empêché(e) de faire ce qui était normalement attendu de vous à cause de votre consommation d'alcool?	○	○	○	○	○
c.	eu besoin d'une boisson alcoolisée le matin pour vous remettre d'une soirée de beuverie?	○	○	○	○	○
d.	eu un sentiment de culpabilité ou de remords après avoir bu de l'alcool?	○	○	○	○	○
e.	été incapable de se souvenir de ce qui s'était passé la veille parce que vous aviez bu de l'alcool?	○	○	○	○	○

F5. Avez-vous ou quelqu'un d'autre été blessé à cause de votre consommation d'alcool?

- ○ Oui, dans les 12 derniers mois
- ○ Oui, mais pas dans des 12 derniers mois
- ○ Non

F6. Est-ce qu'un membre de votre famille, un ami, un médecin ou un autre professionnel de la santé a déjà exprimé son inquiétude au sujet de votre consommation d'alcool, ou vous a suggéré de la réduire?

- ○ Oui, dans les 12 derniers mois
- ○ Oui, mais pas dans les 12 derniers mois
- ○ Non

F6a. Au cours des 12 derniers mois, avez-vous …

Oui **Non**

a.	eu des moments où vous avez fini par boire plus ou pour plus longtemps que prévu?	○	○
b.	essayé de réduire votre consommation d'alcool ou d'arrêter de boire plusieurs fois, mais sans succès?	○	○
c.	passé beaucoup de temps à boire, à être malade ou à combattre les effets secondaires de votre consommation?	○	○
d.	voulu tellement boire un verre que vous ne pouviez penser à rien d'autre?	○	○
e.	trouvé que boire - ou être malade à cause de votre consommation - causait souvent des problèmes avec votre famille, à la maison, au travail ou à l'école?	○	○
f.	continué à boire malgré des problèmes avec votre famille ou vos amis?	○	○
g.	Réduit ou discontinué des activités importantes ou intéressantes pour boire?	○	○
h.	été dans des situations sous influence ou après avoir bu qui augmentaient vos chances de vous blesser (par exemple, conduire, nager, avoir des rapports sexuels à risque)	○	○
i.	continué à boire même si cela vous a rendu déprimé ou anxieux ou même si cela a ajouté à un autre problème de santé?	○	○
j.	dû boire beaucoup plus qu'autrefois pour obtenir l'effet souhaité? Ou avez-vous constaté que votre nombre habituel de boissons avait beaucoup moins d'effet qu'avant?	○	○
k.	trouvé que lorsque les effets de l'alcool se dissipaient, vous aviez du mal à dormir, des tremblements, de l'agitation, des nausées, de la transpiration, un cœur battant, une crise ou des choses ressenties qui n'étaient pas là?	○	○

CKPT.F7.

1. F3a = AU MOINS "MOINS QUE MENSUEL," VA À F7
2. UNE OU PLUS RÉPONSES DANS LA SÉRIE F4a-e = AU MOINS "MOINS QUE MENSUEL," VA À F7
3. F5 OU F6 = "OUI, AU COURS DES 12 DERNIERS MOIS" OU "OUI, MAIS PAS AU COURS DES 12 DERNIERS MOIS," VA À F7
4. TOUS LES AUTRES VA À F11

F7. [(F3a = AU MOINS "MOINS QUE MENSUEL") ET (TOUS RÉSPONSES DANS LA SÉRIE F4a-e = "JAMAIS" OU MANQUANT) ET (F5 = "NON" OU MANQUANT) ET (F6 = "NON" OU MANQANT) ET (A2 = "HOMME"): Vous avez indiqué avoir consommé 5 boissons ou plus lors d'une même occasion./(F3a = AU MOINS "MOINS QUE MENSUEL") ET (TOUS RÉSPONSES DANS LA SÉRIES F4a-e = "JAMAIS" OU MANQUANT) ET (F5 = "NON" OU MANQUANT) ET (F6 = "NON" OU MANQUANT) ET (A2 = "FEMME" OU MANQUANT): Vous avez indiqué avoir consommé 4 boissons ou plus lors d'une même occasion./(F3a = "JAMAIS" OU MANQUANT) ET (F4a = AU MOINS "MOINS QUE MENSUEL") ET (F4b ET F4c ET F4d ET F4e = "JAMAIS" OU MANQUANT) ET (F5 = "NON" OU MANQUANT) ET (F6 = "NON" OU MANQUANT): Vous avez indiqué que vous n'étiez pas capable d'arrêter de boire de l'alcool une fois que vous avez commencé. / (F3a = "JAMAIS" OU MANQUANT) ET (F4b = AU MOINS "MOINS QUE MENSUEL") ET (F4a ET F4c ET F4d ET F4e = "JAMAIS" OU MANQUANT) ET (F5 = "NON" OU MANQUANT) ET (F6 = "NON" OU MANQUANT): Vous avez indiqué que vous avez été empêché(e) de faire ce qui était normalement attendu de vous à cause de votre consommation d'alcool./(F3a = "JAMAIS" OU MANQUANT) ET (F4c = AU MOINS "MOINS QUE MENSUEL") ET (F4a ET F4b ET F4d ET F4e = "JAMAIS" OU MANQUANT) ET (F5 = "NON" OU MANQUANT) ET (F6 = "NON" OU MANQUANT): Vous avez indiqué que vous avez eu besoin d'une boisson alcoolisée le matin pour vous remettre d'une soirée de beuverie./(F3a = "JAMAIS" OU MANQUANT) ET (F4d = AU MOINS "MOINS QUE MENSUEL") ET (F4a ET F4b ET F4c ET F4e = "JAMAIS" OU MANQUANT) ET (F5 = "NON" OU MANQUANT) ET (F6 = "NON" OU MANQUANT): Vous avez indiqué que vous avez eu un sentiment de culpabilité ou de remords après avoir bu de l'alcool./(F3a = "JAMAIS" OU MANQUANT) ET (F4e = AU MOINS "MOINS QUE MENSUEL") ET (F4a ET F4b ET F4c ET F4d = "JAMAIS" OU MANQUANT) ET (F5 = "NON" OU MANQUANT) ET (F6 = "NON" OU MANQUANT): Vous avez indiqué que vous avez été incapable de se souvenir de ce qui s'était passé la veille parce que vous aviez bu de l'alcool. / (F3a = "JAMAIS" OU MANQUANT) ET (TOUS LES RÉPONSES DANS LA SÉRIE F4a-e = "JAMAIS" OU MANQUANT) ET (F5 = "OUI, AU COURS DES 12 DERNIERS MOIS" OU "OUI, MAIS PAS AU COURS DES 12 DERNIERS MOIS") ET (F6 = "NON" OU MANQUANT): Vous avez indiqué que vous avez ou quelqu'un d'autre a été blessé à cause de votre consommation d'alcool./(F3a = "JAMAIS" OU MANQUANT) ET (TOUS

LES RÉPONSES DANS LA SÉRIE IN F4a-e = "JAMAIS" OU MANQUANT) ET (F5 = "NON" OU MANQUANT) ET (F6 = "OUI, AU COURS DES 12 DERNIERS MOIS" OU "OUI, MAIS PAS AU COURS DES 12 DERNIERS MOIS"): Vous avez indiqué que d'autres personnes ont exprimé une inquiétude au sujet de votre consommation d'alcool, ou vous a suggéré de la réduire./TOUS LES AUTRES: Vous avez indiqué avoir vécu quelques expériences associés à la consommation d'alcool (liée a la fréquence de consommation, quantité consommée, comportements qui vous ont inquiété ou qui ont inquiété d'autres personnes).] Environ quel âge aviez-vous <u>la toute première fois</u> que vous avez vécu [(DEUX OU PLUS RÉPONSES EN F3a, DANS LA SÉRIE F4a-e = AU MOINS "MOINS QUE MENSUEL") OU (UNE RÉPONSE EN F3a, DANS LA SÉRIE F4a-e = AU MOINS "MOINS QUE MENSUEL" ET F5 OU F6 = "OUI, AU COURS DES 12 DERNIERS MOIS" OU "OUI, MAIS PAS AU COURS DES 12 DERNIERS MOIS") OU (F5 ET F6 = "OUI, AU COURS DES 12 DERNIERS MOIS" OU "OUI, MAIS PAS AU COURS DES 12 DERNIERS MOIS"): n'importe quelles de ces expériences/TOUS LES AUTRES: cette expérience]?

[LISTE DÉROULANTE] 4 ans ou moins, 5, …, 35, 36 ans ou plus

F8. Au cours de votre vie, pendant environ combien d'années différentes avez-vous vécu [(DEUX OU PLUS RÉPONSES EN F3, DANS LA SÉRIE F4a-e = AU MOINS "MOINS QUE MENSUEL") OU (UNE RÉPONSE EN F3, DANS LA SÉRIE F4a-e = AU MOINS "MOINS QUE MENSUEL" ET F5 OU F6 = "OUI, AU COURS DES 12 DERNIERS MOIS" OU "OUI, MAIS PAS AU COURS DES 12 DERNIERS MOIS") OU (F5 ET F6 = "OUI, AU COURS DES 12 DERNIERS MOIS" OU "OUI, MAIS PAS AU COURS DES 12 DERNIERS MOIS"): n'importe quelles de ces expériences/TOUS LES AUTRES: cette expérience]? *(Si moins d'une année complète, choisissez "1.")*

[LISTE DÉROULANTE] 1, 2, …, 35, 36 ou plus

F9a. Au cours des 30 <u>derniers jours</u>, avez-vous vécu [(DEUX OU PLUS RÉPONSES EN F3, DANS LA SÉRIE F4a-e = AU MOINS "MOINS QUE MENSUEL") OU (UNE RÉPONSE EN F3, DANS LA SÉRIE F4a-e = AU MOINS "MOINS QUE MENSUEL" ET F5 OU F6 = "OUI, AU COURS DES 12 DERNIERS MOIS" OU "OUI, MAIS PAS AU COURS DES 12 DERNIERS MOIS") OU (F5 ET F6 = "OUI, AU COURS DES 12 DERNIERS MOIS" OU "OUI, MAIS PAS AU COURS DES 12 DERNIERS MOIS"): n'importe de quel de ces expériences associées à la consommation d'alcool /TOUS LES AUTRES: cette expérience associée à la consommation d'alcool?

- ○ Oui
- ○ Non

F10.1. Repensez aux périodes de votre vie lorsque vous consommiez le plus souvent les produits suivants pour des raisons <u>médicales,</u> <u>avec</u> ordonnance d'un médecin et comme étant <u>prescrits</u>. Pendant ces périodes, à quelle fréquence consommiez-vous chaque produit suivants?

		Tous les jours ou presque	3-4 jours par semaine	1-2 jours par semaine	1-3 jours par mois	Moins d'une fois par mois	Jamais
a.	Cannabis **avec** ordonnance d'un médecin et **pris comme prescrit**	○	○	○	○	○	○
b.	Tous stimulants pharmaceutiques tel que l'amphétamine ou méthylphénidate, ou modafinil, **avec** ordonnance d'un médecin et **comme prescrits**	○	○	○	○	○	○
c.	Toutes opioïdes pharmaceutiques (par exemple, l'oxycodone, la morphine, l'hydromorphone, la mépéridine, les timbres de fentanyl, la codéine ou tout produit contenant de la codéine tel que le Tylenol #1,	○	○	○	○	○	○

#2, et #3), **avec** ordonnance d'un médecin et **comme prescrits**						
d. Toutes autres drogues pharmaceutiques tel que benzodiazépines [par exemple, lorazepam (Activan), alprazolam (Xanax), diazepam (Valium), clonazepam] ou relaxants musculaires (par exemple, méthocarbamol or baclofèn) ou autre **avec** ordonnance d'un médecin et **comme prescrits**	○	○	○	○	○	○

F10. Maintenant, repensez aux périodes de votre vie lorsque vous consommiez le plus souvent les produits suivant <u>sans</u> ordonnance d'un médecin ou prit à des doses plus grandes que <u>prescrit</u>. Pendant ces périodes, à quelle fréquence consommiez-vous chaque produit suivants?

	**Tous les jours ou presque**	**3-4 jours par semaine**	**1-2 jours par semaine**	**1-3 jours par mois**	**Moins d'une fois par mois**	**Jamais**
a. Tous stimulants pharmaceutiques tel que l'amphétamine ou méthylphénidate, ou modafinil, **sans** ordonnance d'un médecin ou prit à des **doses plus grandes que prescrits** pour être 'high', 'buzzé' ou gelé	○	○	○	○	○	○
b. Toutes opioïdes pharmaceutiques (par exemple, l'oxycodone, la morphine, l'hydromorphone, la mépéridine, les timbres de fentanyl, la codéine ou tout produit contenant de la codéine tel que le Tylenol #1, #2, et #3), **sans** ordonnance d'un médecin ou prit à des **doses plus grandes que prescrits**	○	○	○	○	○	○
c. Toutes autres drogues pharmaceutiques tel que benzodiazépines [par exemple, lorazepam (Activan), alprazolam (Xanax), diazepam (Valium), clonazepam] ou relaxants musculaires (par exemple, méthocarbamol or baclofèn) ou autre **sans** ordonnance du médecin ou prit à des **doses plus grandes que prescrits** pour être 'high', 'buzzé' ou gelé	○	○	○	○	○	○
d. Cannabis (marijuana, pot, haschich, beuh, etc.) pour des raisons non-médicales	○	○	○	○	○	○
e. Tous stimulants de la rue, tel que la cocaïne, la méthamphétamine, le crack, crystal meth, et tous autres stimulants obtenus "dans la rue"	○	○	○	○	○	○

f.	Toutes opioïdes de la rue, tel que l'héroïne, fentanyl ou toutes autres opioïdes obtenues "dans la rue"	○	○	○	○	○	○
g.	Toutes autres drogues de la rue (e.g., LSD, ecstasy, PCP, champignon hallucinogène, autres)	○	○	○	○	○	○

CKPT.F12.

1. UNE OU PLUS RÉPONSES DANS LA SÉRIE F11.1a-d = AU MOINS "MOINS D'UNE FOIS PAR MOIS," VA À F12
2. TOUS LES AUTRES VA À CKPT.F18

F11. Repensez à l'année durant laquelle votre consommation de drogues a le plus perturbé votre vie. Pendant cette année-là, à quelle fréquence avez-vous rencontré les problèmes suivants?

		Tous les jours ou presque	3-4 jours par semaine	1-2 jours par semaine	1-3 jours par mois	Moins d'une fois par mois	Jamais
a.	À quelle fréquence votre consommation de drogues a-t-elle interféré avec vos responsabilités à la maison, au travail ou à l'école?	○	○	○	○	○	○
b.	À quelle fréquence avez-vous continué à consommer des substances, malgré les disputes ou autres problèmes sérieux avec votre famille ou vos amis?	○	○	○	○	○	○
c.	À quelle fréquence avez-vous été sous l'influence dans une situation où vous auriez pu vous faire mal?	○	○	○	○	○	○
d.	À quelle fréquence avez-vous considérablement diminué vos activités importantes avec votre famille, vos amis, ou au travail à cause de votre consommation de drogues?	○	○	○	○	○	○
e.	À quelle fréquence avez-vous consommé plus que prévu ou passé plus de temps que prévu à consommer?	○	○	○	○	○	○

F12. Pendant cette année-là, à quelle fréquence avez-vous rencontré les problèmes suivants?

		Tous les jours ou presque	3-4 jours par semaine	1-2 jours par semaine	1-3 jours par mois	Moins d'une fois par mois	Jamais
a.	À quelle fréquence avez-vous passé beaucoup de temps à obtenir de la drogue, à utiliser de la drogue, ou à récupérer des effets de votre consommation de substances?	○	○	○	○	○	○
b.	À quelle fréquence avez-vous eu une envie irrésistible ou un fort désir de consommer des substances?	○	○	○	○	○	○
c.	À quelle fréquence avez-vous ressenti le besoin de réduire ou	○	○	○	○	○	○

	d'arrêter votre consommation de substances?						
d.	À quelle fréquence avez-vous continué à utiliser des substances, alors que cela causait ou empirait un problème de santé physique ou émotionnelle?	○	○	○	○	○	○
e.	À quelle fréquence avez-vous développé une tolérance? C'est-à-dire, la même quantité n'a plus le même effet, ou il faut des quantités notablement plus fortes pour obtenir le même effet?	○	○	○	○	○	○
f.	À quelle fréquence avez-vous eu des symptômes de sevrage tels que des troubles du sommeil, des problèmes émotionnelles, de l'agitation, des sueurs ou de la nausée lorsque vous avez essayé de réduire votre consommation de drogue, ou avez-vous continué à consommer pour éviter les symptômes de sevrage?	○	○	○	○	○	○

CKPT.F14.

1. DEUX OU PLUS RÉPONSES DANS LES SÉRIES F12a-e, F13a-f = AU MOINS "MOINS D'UNE FOIS PAR MOIS," VA À F14
2. TOUS LES AUTRES VA À CKPT.F18.

F13. Vous avez indiqué avoir eu des problèmes liés à votre consommation de drogues. Environ quel âge aviez-vous environ <u>la toute première fois</u> que vous avez eu n'importe quelles de ces problèmes?

[LISTE DÉROULANTE] 4 ans ou moins, 5, …, 35, 36 ans ou plus

F14. **Au cours de votre vie, pendant environ combien d'années différentes avez-vous eu ces problèmes?**

[LISTE DÉROULANTE] 1, 2, …, 35, 36 ou plus

CKPT.F17.

1. F16 = 1-11, VA À F17
2. TOUS LES AUTRES VA À CKPT.F18

F15a2. Laquelle de ces expériences avez-vous vécues au cours des 30 derniers jours et / ou 12 derniers mois?

		Oui dans les 30 derniers jours	Oui dans les 12 derniers mois mais non dans les 30 derniers jours	Non dans les 12 derniers mois
a.	Vous avez passé beaucoup de temps à obtenir ou utiliser des drogues ou récupérer de la consommation de drogues	○	○	○
b.	Vous avez un fort désir ou une envie d'utiliser	○	○	○
c.	Vous avez ressenti le besoin de réduire ou d'arrêter votre consommation de drogue	○	○	○

d. Vous avez continué à utiliser même si cela a causé ou aggravé un problème de santé physique ou émotionnel	○	○	○
e. Vous avez développé une tolérance; c'est-à-dire que soit le même montant n'avait plus le même effet, soit vous deviez en utiliser beaucoup plus pour obtenir le même effet	○	○	○
f. Votre consommation de drogue ou votre état d'influence ont interféré avec vos responsabilités à la maison, au travail ou à l'école	○	○	○
g. Vous avez continué à utiliser même si cela provoquait des disputes ou d'autres problèmes graves avec votre famille ou vos amis	○	○	○
h. Vous avez considérablement réduit des activités importantes en famille, entre amis ou au travail en raison de votre consommation de drogue	○	○	○
i. You greatly reduced important activities with family, friends, or at work because of your drug use	○	○	○
j. Vous avez utilisé plus ou passé plus de temps que prévu lorsque vous avez commencé	○	○	○
k. Vous avez eu des troubles du sommeil, des problèmes émotionnels, de l'agitation, de la transpiration ou des nausées lorsque vous avez essayé de réduire votre consommation, ou vous avez continué à utiliser afin d'éviter ces symptômes de sevrage?	○	○	○

CKPT.F18.

1. (DEUX OU PLUS RÉSPONSES DANS LA SÉRIE F11.1a-g = AU MOINS "MOINS QU'UNE FOIS PAR MOIS") ET (DEUX OU PLUS RÉSPONSES DANS LES SÉRIES F12a-e, F13a-f = AU MOINS "MOINS QU'UNE FOIS PAR MOIS"), VA À F18
2. F11a OU F11.1d = AU MOINS "MOINS QU'UNE FOIS PAR MOIS," VA À F19
3. UN OU PLUS RÉSPONSES DANS LA SÉRIE F11a-d ET/OU DANS LA SÉRIE F11.1a-g = AU MOINS "MOINS QU'UNE FOIS PAR MOIS," VA À F22
4. F1 = AU MOINS "MENSUEL OU MOINS" OU F2 = 1 OU PLUS, VA À F22
5. TOUS LES AUTRES VA À LA PROCHAINE SECTION

F18. Plus tôt, vous avez indiqué avoir consommé plus qu'une drogue. Quelle drogue a causé la majorité des expériences que vous avez décrit plus haut? *(il peut y en avoir plusieurs, cochez toutes les propositions qui vous concernent)*

- [ ] Cannabis pour des raisons non-médicales
- [ ] Stimulants (y compris des stimulants pharmaceutiques, sans ordonnance et les drogues de la rue)
- [ ] Opioïdes (y compris des opioïdes pharmaceutiques, sans ordonnance et les drogues de la rue)
- [ ] Toutes autres drogues sur ordonnance ou sans ordonnance
- [ ] Toutes autres drogues de la rue

CKPT.F19.

1. F11a OU F11.1d = AU MOINS "MOINS QU'UNE FOIS PAR MOIS," VA À F19
2. TOUS LES AUTRES VA À F22

F19. Vous avez indiqué avoir consommé du cannabis. Sachiez-vous que le cannabis a deux ingredients actifs principaux , le tétrahydrocannabinol (THC) et le cannabidiol (CBD), et que leurs effets sont différents?

- ○ Oui, je sais qu'il existe deux ingrédients actifs et que leurs effets sont différents
- ○ Oui, je sais qu'il existe deux ingrédients actifs mais pas que leurs effets sont différents
- ○ Non, je ne savais pas qu'il existe deux ingrédients actifs

CKPT.F20.

1. F19 ≠ "NON, JE NE SUIS PAS CONSCIENT DE LEURS EXISTENCE", VA À F20
2. TOUS LES AUTRES VA À F21

F20. Si vous pourriez choisir, choisissiez-vous une teneur plus élevée en tétrahydrocannabinol (THC) ou cannabidiol (CBD)?

- ○ THC plus élevée
- ○ CBD plus élevée
- ○ Ça n'a pas d'importance ou je ne suis pas sûr

F21. Parmi les affirmations suivantes, laquelle est(sont) vraie(s) à propos de votre consommation de cannabis? *(Cochez toutes les propositions qui vous concernent)*

- ☐ J'utilise/j'ai utilisé du cannabis synthétique (spice, K2, etc.)
- ☐ Je fume du cannabis principalement (i.e., un joint)
- Je fume du cannabis et du tabac ensemble, en combinaison
- ☐ Quand je fume du cannabis, j'inspire profondement et/ou je retiens mon souffle pour augmenter l'effet
- Principalement je vape les feuilles ou les fleurs de la plante ou je consomme des mangeables
- ☐ Je vape principalement de l'huile de cannabis, du cannabis concentré, ou je consomme d'autres formes de cannabis
- ☐ Je consomme du cannabis seulement une fois par semaine, le week-end, ou moins

F22. En ce qui concerne la conduite ou l'utilisation de machinerie lourdes

- ○ Je ne conduis jamais et n'utilise jamais de la machinerie lourde après avoir consommé de l'alcool ou des drogues (ou je ne conduis pas ou n'utilise jamais de machines)
- ○ Je conduis ou j'utilise de la machinerie lourde après avoir consommé de l'alcool ou des drogues qu'exceptionnellement
- ○ Je conduis ou j'utilise de la machinerie lourde après avoir consommé de l'alcool ou des drogues parfois

CKPT.F23.

1. A2 = "FEMME" ET F1 = AU MOINS "MENSUEL OU MOINS" OU F2 = 1 OU PLUS, VA À F23
2. A2 = "FEMME" ET UNE OU PLUS RÉSPONSES DANS LA SÉRIE F11.1a-g = AU MOINS "MOINS QU'UNE FOIS PAR MOIS," VA À F23
3. TOUS LES AUTRES VA À CKPT.F24

F23. Avez-vous consommé de l'alcool ou des drogues pendant votre grossesse ou allaitement. Pour cette question, considérer seulement les drogues de la rue, les autres drogues sans ordonnance d'un médecin, ou les drogues prisent dans des quantités plus grandes que prescrit?

- ○ Je n'ai jamais consommé de l'alcool ou des drogues en étant enceinte ou allaitement
- ○ Parfois, j'ai consommé de l'alcool ou des drogues en étant enceinte ou allaitement
- ○ Rarement, j'ai consommé de l'alcool ou des drogues en étant enceinte ou allaitement

- Je n’ai jamais été enceinte ou allaitante

CKPT.F24.
1. F11c OU F11.1b OU F11.1f = AU MOINS “MOINS QUE MENSUEL,” VA À F24
2. TOUS LES AUTRES VA À LA PROCHAINE SECTION

F24. Vous avez indiqué avoir consommé des opioïdes. Parmi les affirmations suivantes, laquelle est vraie à propos de votre première consommation d’opioïdes?

- Ils m’ont été prescrits pour la douleur lié au cancer
- Ils m’ont été prescrits pour la douleur non cancéreuse (par exemple, lésion sportif, douleur du dos, etc.)
- Ils ont été prescrits à un membre de la famille, une colocataire ou une connaissance proche qui les ont partagés avec moi ou de qui je les ai pris
- Une personne qui les consommaient pour se detendre, faire la fête, ou pour une raison non-médicale me les a offerts
- Je les ai acheté sans ordonance, dans la rue ou je les ai pris de quelqu’un sans leur connaissance
- Autre. Veuillez expliquer:

CKPT.F25.
1. F24= “Ils m’ont été prescrit pour la douleur non cancéreuse”, VA À F24
2. TOUS LES AUTRES VA À LA PROCHAINE SECTION

F25. Quelle sorte de douleur non cancéreuse avez-/aviez-vous?
- Des muscles, des articulations, du tissue mous, résultant par exemple de blessure sportives ou professionnelles, fibromyalgie, de douleurs chronique de dos ou cous, etc.
- Neuropathique, due par exemple au diabète, à une lésion nerveuse, ou infections
- Douleur visceral, due par exemple à des lesions ou à une maladie des organes internes
- Douleur postopérative
- Autre

F26. Quand vous avez consulté un médecin pour la première fois pour une telle douleur, avez-vous reçu des médicaments non-opioïdes (tel que l’acétaminophène ou des anti-inflammatoires non-stéroïdiens, y compris l’ibuprofène, le diclofénac, le célécoxib, etc. ou des antidépresseurs) et des interventions non pharmacologiques (tel que l’exercice, le yoga, le Tai-Chi, le massage, ou l’acupuncture)?

- Oui, j’ai eu des expériences positives et bénéfiques avec des médicaments anti-inflammatoires non-opioïdes et des interventions non pharmacologiques (c’est-à-dire des interventions surveiller de manière adéquate pendant un mois ou plus) **avant** recourir aux opioïdes
- Oui, j’ai brièvement essayé des médicaments anti-inflammatoires non-opioïdes et des intervention non pharmacologiques (i.e., pour des jours à des semaines), mais rapidement conclus qu’ils ne marchaient pas pour moi
- Non, on m’a prescrit des opioïdes directement

F27. Vous souvenez-vous du nom et de la dose quotidienne que vous avez eu quand vous avez commencé votre traitement aux opioïdes?
- Morphine, 50mg ou moins
- Morphine, plus de 50mg
- Morphine, ne me souviens pas de la dose
- Codéine, 300mg ou moins
- Codéine, plus de 300mg
- Codéine, ne me souviens pas de la dose
- Oxycodone, 30mg ou moins
- Oxycodone, plus de 30mg
- Oxycodone, ne me souviens pas de la dose
- Hydromorphone, 10mg ou moins
- Hydromorphone, plus de 10mg

- Hydromorphone, ne me souviens pas de la dose
- Tramadol, 300mg ou moins
- Tramadol, plus de 300mg
- Tramadol, ne me souviens pas de la dose
- Timbre transdermique de Fentanyl, 25mcg/h ou moins
- Timbre transdermique de Fentanyl, plus de 25mcg/h
- Fentanyl, ne me souviens pas de la dose
- Autre, ne me souviens pas

F28. Pendant votre traitement aux opioïdes, votre médecin a-t-il maintenu la drogue et la dose initiale, passé à un autre opioïde, ou augmenté la dose de manière significative?

- Mon médecin a maintenu la dose initiale et ceci a bien contrôler ma douleur
- Mon médecin a maintenu la dose initiale malgré ne pas réussir à contrôler la douleur
- Mon médecin est passé à un ou plusieurs opioïdes à cause de douleurs persistantes
- Mon médecin a augmenté la dose de manière significative à cause de douleurs persistantes
- Ne sais pas

F29. Votre médecin a-t-il tenté de diminuer progressivement ou d'arrêter votre traitement à base d'opioïdes?

- Oui, nous avons réussi à réduire et arrêter mon traitement à base d'opioïdes. Je n'ai jamais utilisé d'opioïdes depuis.
- Oui, nous avons réussi à réduire et arrêter mon traitement à base d'opioïdes. J'ai cependant eu besoin de recommencer à cause de douleurs.
- Oui, nous avons réussi à réduire la dose à la plus faible dose, que je prends encore maintenant.
- Oui, nous avons essayé de réduire la dose mais nous n'avons pas réussi à cause de douleurs ou à cause des effets d'abstinence, alors je prends encore des opioïdes.
- Oui, nous avons essayé de réduire la dose mais nous n'avons pas réussi à cause de douleurs ou à cause des effets d'abstinence, alors j'ai été dirigé(e) un programme multidisciplinaire spécialisé.
- Non, mon médecin n'a pas essayé de réduire la dose, je prends encore la même dose ou une dose plus forte.
- Non, mon médecin n'a pas essayé de réduire la dose. J'ai finalement arrêté de consommer les opioïdes moi-même.

F30. Votre douleur est-elle sous contrôle maintenant?

- Oui, complètement
- Oui, partiellement
- Pas du tout

F31. Consommez-vous des opioïdes maintenant?

- Oui, des opioïdes pharmaceutiques de le rue
- Oui, des opioïdes pharmaceutiques, tel que prescrits
- Oui, des opioïdes sur ordonnance, des opioïdes sans ordonnance ou en plus grandes quantités que prescrits
- Non

FIN DE SECTION

SECTION G: AUTOMUTILATION

G1. Au cours de votre vie, avez-vous déjà souhaité être mort ou souhaité de vous endormir et ne jamais vous réveiller?

- ○ Oui
- ○ Non

G2. Au cours de votre vie, avez-vous déjà pensé à mettre fin à vos jours?

- ○ Oui
- ○ Non

CKPT.G3.

1. G1 OU G2 = "OUI," VA À G3
2. TOUS LES AUTRES VA À G16

G3. Quel âge aviez-vous environ <u>la toute première fois</u> que vous avez (G1 = "OUI" ET G2 = "NON" OU MANQUANT: souhaité être mort ou de vous endormir et ne jamais vous réveiller/G1 = "NON" OU MANQUANT ET G2 = "OUI": eu des pensées suicidaires/TOUS LES AUTRES: souhaité être mort, souhaité de vous endormir et ne jamais vous réveiller, ou eu des pensées suicidaires)?

[LISTE DÉROULANTE] 4 ans ou moins, 5, …, 35, 36 ans ou plus

G4. Au cours de votre vie, pendant environ combien d'années différentes avez-vous (G1 = "OUI" ET G2 = "NON" OU MANQUANT: souhaité être mort ou de vous endormir et ne jamais vous réveiller/G1 = "NON" OU MANQUANT ET G2 = "OUI": eu des pensées suicidaires/TOUS LES AUTRES: soit souhaité être mort, souhaité de vous endormir et ne jamais vous réveiller, ou eu des pensées suicidaires)?
(Si moins d'une année complète, choisissez "1.")

[LISTE DÉROULANTE] 1, 2, …, 35, 36 ou plus

G5. Au cours des <u>12 derniers mois,</u> pendant environ combien de mois avez-vous (G1 = "OUI" ET G2 = "NON" OU MANQUANT: souhaité être mort ou de vous endormir sans jamais vous réveiller/G1 = "NON" OU MANQUANT ET G2 = "OUI": eu des pensées suicidaires/TOUS LES AUTRES: soit souhaité être mort, souhaité de vous endormir et ne jamais vous réveiller, ou eu des pensées suicidaires)?
[LISTE DÉROULANTE] 0, 1, …, 11, 12

CKPT.G6.

1. G5 = 1-12, VA À G6
2. TOUS LES AUTRES VA À G7

G6. Au cours des 30 <u>derniers jours</u>, à quelle fréquence avez-vous (G1 = "OUI" ET G2 = "NON" OU MANQUANT: souhaité être mort ou de vous endormir et ne jamais vous réveiller/G1 = "NON" OU MANQUANT ET G2 = "OUI": avez eu des pensées suicidaires/TOUS LES AUTRES: avez souhaité être mort ou souhaité vous endormir et ne jamais vous réveiller ou avez eu des pensées suicidaires)?

- ○ Tout ou presque tout le temps
- ○ La plupart du temps
- ○ Une partie du temps
- ○ De temps en temps
- ○ Jamais

G7. **Avez-vous déjà réfléchi à la manière dont vous pourriez mettre fin à vos jour (par exemple, prendre des medicaments, vous tirer dessus avec une arme à feu) ou conçu un scenario concernant la manière de mettre fin à vos jours?**

- ○ Oui
- ○ Non

CKPT.G8.

1. G7 = "NON" OU MANQUANT, VA À G12
2. TOUS LES AUTRES VA À G8

G8. **Quel âge aviez-vous environ <u>la toute première fois</u> que vous avez réfléchi à la manière dont vous pourriez mettre fin à vos jours ou conçu un scenario concernant la manière de mettre fin à vos jours?**

[LIST DÉROULANTE] 4 ans ou moins, 5, …, 35, 36 ans ou plus

G9. **Au cours de votre vie, pendant environ combien d'années différentes avez-vou réfléchi à la manière dont vous pourriez mettre fin à vos jours ou conçu un scenario concernant la manière de mettre fin à vos jours?** *(Si moins d'une année complète, choisissez "1.")*

[LISTE DÉROULANTE] 1, 2, …, 35, 36 ou plus

G10. **Au cours des <u>12 derniers mois,</u> pendant environ combien de mois avez-vous réfléchi à la manière dont vous pourriez mettre fin à vos jours ou conçu un scenario concernant la manière de mettre fin à vos jours?**

[LISTE DÉROULANTE] 0, 1, …, 11, 12

G11. **Dans quelle mesure pensez-vous mettre ce plan en œuvre dans les <u>12 prochains mois</u>?**

- ○ Très probable
- ○ Assez probable
- ○ Peu probable
- ○ Pas du tout probable

G12. **Repensez à la semaine de votre vie quand vous avez le plus réfléchi (G1 = "OUI" ET G2 = "NON" OU MANQUANT: à vouloir être mort/G1 = "NON" OU MANQUANT ET G2 = "OUI": à mettre fin à vos jours/TOUS LES AUTRES: à vouloir être mort ou à mettre fin à vos jours). Combien de jours pendant cette semaine avez-vous eu ces pensées?**

[LISTE DÉROULANTE] 1, …, 6, 7

G13. **(G12 ≥ 2 ET ≠ MANQUANT: Au cours de cette semaine, pendant combien de temps est-ce que ces pensées ont-elles généralement durées les jours quand vous les avez eus? /TOUS LES AUTRES: Au cours de cette semaine, pendant combien de temps est-ce que ces pensées ont-elles généralement durées les jours quand vous les avez eus?)**

- ○ Seulement quelque secondes ou minutes
- ○ Moins de 1 heure
- ○ 1-4 heures
- ○ 5-8 heures
- ○ 9 ou plusieurs heures

G14. Pendant cette semaine, étiez-vous capable de contrôler ces pensées ou de les faire sortir de votre esprit lorsque vous le vouliez?

- ◯ Facile
- ◯ Un peu difficile
- ◯ Quelque peu difficile
- ◯ Assez difficile
- ◯ Impossible; incapable de contrôler les pensées

CKPT.G15.

1. G1 OU G2 = "OUI," VA À G15
2. TOUS LES AUTRES VA À G16

G15. Les personnes qui pensent au suicide ou qui ont envie de mourir font parfois des choses dangereuses pour tenter leur destin (par exemple, prendre beaucoup de drogues, conduire trop vite, se porter volontaire pour des missions dangereuses, se comporter imprudemment). Dans votre vie, à quelle fréquence avez-vous déjà fait des choses dangereuses comme celle-ci pour tenter votre destin?

- ◯ Très souvent
- ◯ Souvent
- ◯ Parfois
- ◯ Rarement
- ◯ Jamais

G16. Avez-vous déjà essayé de mettre fin à votre vie (vous blesser délibérément avec une intention de mourir)?

- ◯ Oui
- ◯ Non

CKPT.G17.

1. G16 = "NON" OU MANQUANT, VA À G20
2. TOUS LES AUTRES VA À G17

G17. Quel âge aviez-vous environ <u>la toute première fois</u> que vous avez essayé de mettre fin à votre vie?

[LISTE DÉROULANTE] 4 ans ou moins, 5, …, 35, 36 ans ou plus

G18. Au cours de votre vie, combien de fois avez-vous déjà essayé de mettre fin à votre vie?

_____ TENTATIVE(S)

G19. Au cours des <u>12 derniers mois</u>, combien de fois avez-vous déjà essayé de mettre fin à votre vie?

_____ TENTATIVE(S) AU COURS DES 12 DERNIERS MOIS

G20. Au cours de votre vie, avez-vous déjà fait quelque chose pour vous blesser délibérément, pour vous faire du mal intentionnellement, sans pour autant vouloir mourir (par exemple, vous couper, vous frapper, vous brûler)?

- ◯ Oui

○ Non

CKPT.G21.

1. G20 = "NON" OU MANQUANT, VA À LA PROCHAINE SECTION
2. TOUS LES AUTRES VA À G21

G21. Quel âge aviez-vous environ <u>la toute première fois</u> que vous avez fait quelque chose pour vous faire du mal intentionnellement, sans pour autant vouloir mourir?

[LISTE DÉROUALNTE] 4 ans ou moins, 5, …, 35, 36 ans ou plus

G22. Au cours de votre vie, combien de fois avez-vous fait quelque chose pour vous faire du mal intentionnellement, sans pour autant vouloir mourir?

[LISTE DÉROULANTE]

○ 1-2 fois
○ 3-5 fois
○ 6-10 fois
○ 11-20 fois
○ 21-30 fois
○ 31-50 fois
○ 51-100 fois
○ 101 fois ou plus

G23. Au cours des <u>12 derniers mois,</u> combien de fois avez-vous fait quelque chose pour vous faire du mal intentionnellement, sans pour autant vouloir mourir?

[LISTE DÉROULANTE]

○ 0 fois
○ 1-2 fois
○ 3-4 fois
○ 5-10 fois
○ 11-20 fois
○ 21-30 fois
○ 31-50 fois
○ 51-100 fois
○ 101 fois ou plus

FIN DE SECTION

SECTION H: CHERCHER DE L'AIDE

H1. **Dans votre vie, avez-vous déjà reçu du soutien psychologique ou des médicaments pour un problème émotionnel ou lié à votre consommation d'alcool ou de drogues?**

		Oui	Non
a.	Soutien psychologique	○	○
b.	Médicaments	○	○

CKPT.H2.
1. H1a = "NON" OU MANQUANT ET H1b = "NON" OU MANQUANT, VA À H6
2. TOUS LES AUTRES VA À H2

H2. **Quel âge aviez-vous environ <u>la toute première fois</u> que vous avez reçu du soutien psychologique ou des médicaments pour un problème émotionnel ou lié à votre consommation d'alcool ou de drogues?**

[LISTE DÉROULANTE] 4 ans ou moins, 5, …, 35, 36 ans ou plus

H3. **Au cours des <u>12 derniers mois,</u> pendant environ combien de mois avez-vous reçu du soutien psychologique ou des médicaments?**

[LISTE DÉROULANTE] 0, 1, …, 11, 12

CKPT.H4.
1. H3 = 1-12, VA À H5
2. TOUS LES AUTRES VA À H4

H4. **Quel âge aviez-vous environ <u>la toute dernière fois</u> que vous avez reçu du soutien psychologique ou des médicaments pour un problème émotionnel ou lié à votre consommation d'alcool ou de drogues?**

[LISTE DÉROULANTE] 4 ans ou moins, 5, …, 35, 36 ans ou plus

H5. **Recevez-vous toujours ce traitement ou avez-vous arrêté?**

○ Toujours en traitement
○ Traitement arrêté

CKPT.H6.
1. H5 = "TOUJOURS EN TRAITEMENT," VA À H9
2. TOUS LES AUTRES VA À H6

H6. **Comment qualifierez-vous votre volonté de changer tout problème émotionnel ou lié à votre consommation d'alcool ou de drogues?**

○ Je n'ai pas de problème émotionnel ou lié à ma consommation d'alcool ou de drogues que je doive changer
○ J'ai un problème, mais je ne suis pas encore certain de vouloir faire quelque chose pour le changer
○ J'ai un problème et j'ai l'intention de le changer
○ J'ai un problème et je travaille déjà activement pour le changer

- ◯ J'ai eu un problème mais je m'en suis occupé(e) et les choses vont mieux maintenant

CKPT.H7.
1. H3 = 1-12, VA À H9
2. TOUS LES AUTRES VA À H7

H7. Au cours des <u>12 derniers mois</u>, y a-t-il eu un moment pendant lequel vous avez senti besoin d'un soutien psychologique ou des médicaments pour un problème émotionnel ou lié à votre consommation d'alcool ou de drogues?

- ◯ Oui
- ◯ Non ⟶ VA À H9

[PROGRAMMEUR: PASSER MANQUANT AVEC "NON"]

H8. Quelle importance pensez-vous qu'a eu chacune de des raisons suivantes pour NE PAS avoir chercher de l'aide pour votre (vos) problème(s)?

		Grande importance	Important	Moyenne importance	Peu d'importance	Pas importance
a.	Vous n'étiez pas sûr(e) que les traitements disponibles étaient efficaces	◯	◯	◯	◯	◯
b.	Vous vouliez gérer le problème vous-même	◯	◯	◯	◯	◯
c.	Vous étiez trop gêné(e)	◯	◯	◯	◯	◯
d.	Vous avez parlé à des amis ou à des proches à la place	◯	◯	◯	◯	◯
e.	C'est trop cher	◯	◯	◯	◯	◯
f.	Vous n'étiez pas sûr(e) d'où aller ou qui voir	◯	◯	◯	◯	◯
g.	Vous avez eu des problèmes de temps, de transport ou d'horaire	◯	◯	◯	◯	◯
h.	Vous craigniez que cela nuise à votre carrière scolaire ou professionnelle	◯	◯	◯	◯	◯
i.	Vous craigniez que les gens vous considèrent différent et vous traiterez différemment s'ils savaient que vous étiez en traitement	◯	◯	◯	◯	◯
j.	Une autre raison *(Merci de préciser dans la case ci-dessous)*	◯	◯	◯	◯	◯

H9. Avez-vous déjà chercher pour de l'information, de l'aide ou des traitements, pour n'importe quel problème émotionnel ou lié à votre consommation sur l'internet, à travers des applications ou des outils en ligne?

- ◯ Oui
- ◯ Non

H10. Maintenant ou à l'avenir, si vous vouliez recevoir du soutien pour gérer votre stress, de l'aide pour faire face aux problèmes émotionnels ou liés à la consommation, ou psychothérapie, dans quelle mesure pensez-vous accéder à une ressource en santé mentale sur l'internet, tel qu'une application ou un outil en ligne?

- ○ Très probable
- ○ Assez probable
- ○ Peu probable
- ○ Pas du tout probable

FIN DE SECTION

SECTION I: VOTRE MILIEU D'ENFANCE

I1. Votre mère est-elle toujours en vie ou est-elle décédée?

○ En vie
○ Décédée

CKPT.I1a.
1. I1 = "VIVANT" OU MANQUANT, VA À I1c
2. TOUS LES AUTRES VA À I1b

I1b. Quel était son niveau d'éducation?

○ Aucun
○ École primaire
○ École secondaire
○ Post-secondaire mais sans diplôme universitaire (CEGEP)
○ Diplôme universitaire
○ Doctorat
○ Je ne sais pas

VA À I2

I1c. Quel est son niveau d'éducation?

○ Aucun
○ École primaire
○ École secondaire
○ Post-secondaire mais sans diplôme universitaire (CEGEP)
○ Diplôme universitaire
○ Doctorat
○ Je ne sais pas

I2. Votre père est-il toujours en vie ou est-il décédé?

○ En vie
○ Décédé

CKPT.I2a.
1. I2 = "VIVANT" OU MANQUANT, VA À I2c
2. TOUS LES AUTRES VA À I2b

I2b. Quel était son niveau d'éducation?

○ Aucun
○ École primaire
○ École secondaire
○ Post-secondaire mais sans diplôme universitaire (CEGEP)
○ Diplôme universitaire

- ○ Doctorat
- ○ Je ne sais pas

VA À CKPT.I3a

I2c. Quel est son niveau d'éducation?

- ○ Aucun
- ○ École primaire
- ○ École secondaire
- ○ Post-secondaire mais sans diplôme universitaire (CEGEP)
- ○ Diplôme universitaire
- ○ Doctorat
- ○ Je ne sais pas

CKPT.I3a.

1. I1 = "DÉCÉDÉE" ET I2 = "VIVANTE" OU MANQUANT, VA À I3a
2. I1 = "ALIVE" OU MANQUANT ET I2 = "DECEASED," VA À I3b
3. I1 = "DÉCÉDÉE" ET I2 = "DÉCÉDÉE," VA À I3c
4. I1 = "VIVANTE" OU MANQUANT ET I2 = "VIVANTE" OU MANQUANT, VA À I3d

I3a. Lorsque votre mère est décédée, est-ce que vos parents étaient mariés, ou étaient-ils séparés ou divorcés?

- ○ Mariés
- ○ Séparés
- ○ Divorcés
- ○ Ils n'ont jamais été mariés

VA À I4

I3b. Lorsque votre père est décédé, est-ce que vos parents étaient mariés, ou étaient-ils séparés ou divorcés?

- ○ Mariés
- ○ Séparés
- ○ Divorcés
- ○ Ils n'ont jamais été mariés

VA À I4

I3c. Lorsque votre premier parent est décédé, est-ce que vos parents étaient mariés, ou étaient-ils séparés ou divorcés?

- ○ Mariés
- ○ Séparés
- ○ Divorcés
- ○ Ils n'ont jamais été mariés

VA À I4

I3d. Vos parents sont-ils mariés, séparés ou divorcés?

- ○ Mariés
- ○ Séparés
- ○ Divorcés
- ○ Ils n'ont jamais été mariés

I4. Avant l'âge de 18 ans, à quelle fréquence avez-vous vécu chacune des expériences suivantes?

		Très souvent	Souvent	Parfois	Rarement	Jamais
a.	Un de vos parents (ou une des personnes qui vous ont élevée) avait un grave problème émotionnel ou de santé mentale	○	○	○	○	○
b.	Un de vos parents (ou une des personnes qui vous ont élevée) avait un grave problème d'alcool ou de drogues	○	○	○	○	○
c.	Un de vos parents (ou une des personnes qui vous ont élevée) a tenté de se suicider ou est décédé(E) suite à un suicide	○	○	○	○	○
d.	Un de vos parents (ou une des personnes qui vous ont élevée) a été impliqué(e) dans des activités criminelles	○	○	○	○	○
e.	Vos parents (ou une des personnes qui vous ont élevée) se frappaient ou étaient violents l'un envers l'autre	○	○	○	○	○
f.	Quelqu'un dans votre famille vous a frappé si fort que cela a laissé des bleus ou des marques	○	○	○	○	○

I5. Avant l'âge de 18 ans, à quelle fréquence avez-vous vécu chacune des expériences suivantes?

		Très souvent	Souvent	Parfois	Rarement	Jamais
a.	Vous avez vécu de la maltraitance physique à la maison	○	○	○	○	○
b.	Quelqu'un dans votre famille vous répétait des choses blessantes ou insultantes	○	○	○	○	○
c.	Vous avez vécu de la maltraitance psychologique ou émotionnelle à la maison	○	○	○	○	○
d.	Vous vous êtes sentis spécial ou important à cause de quelqu'un dans votre famille	○	○	○	○	○
e.	Quelqu'un dans votre famille vous a touché sexuellement, ou vous a forcé à les toucher de manière sexuelle contre votre gré	○	○	○	○	○

I6. Avant l'âge de 18 ans, à quelle fréquence avez-vous vécu chacune des expériences suivantes?

		Très souvent	Souvent	Parfois	Rarement	Jamais
a.	Vous avez vécu de la maltraitance sexuelle à la maison	○	○	○	○	○
b.	Vous avez été sérieusement négligé(e) à la maison (par exemple, personne ne s'occupait de vous ou ne vous protégeait ou s'assurait que vous aviez ce dont vous aviez besoin)	○	○	○	○	○
c.	Vous avez dû faire des tâches ménagères ou des corvées trop difficiles ou dangereuses pour quelqu'un de votre âge	○	○	○	○	○
d.	Vous vous sentiez aimé(e) et entouré(e) par votre famille/à la maison	○	○	○	○	○
e.	Les personnes de votre famille veillaient sur vous et prenaient soin de vous	○	○	○	○	○
f.	Vous vous sentiez émotionnellement proche avec les membres de votre famille	○	○	○	○	○

I7. Avez-vous été victime d'abus physique ou sexuel en dehors de votre domicile ou par des personnes hors famille avant l'age de 17 ans (inclus)?

○ Non
○ Oui, physiquement
○ Oui, sexuellement
○ Oui, physiquement et sexuellement

FIN DE SECTION

SECTION J: EXPERIENCES RÉCENTES

J1. Avez-vous vécu une des situations stressantes suivantes au cours des 12 derniers mois?

		Oui	Non
a.	Une maladie ou blessure mettant en jeu la vie d'un ami proche ou d'un membre de votre famille	○	○
b.	La mort d'un ami ou d'un membre de votre famille	○	○
c.	Une séparation avec un(e) partenaire amoureux(se)	○	○
d.	Vous avez découvert qu'un partenaire amoureux(se) vous a trompé(e)	○	○
e.	Une trahison sérieuse de la part d'une personne proche	○	○
f.	Des disputes persistantes et importantes ou des séparations persistantes sérieuses d'autres amis proches ou membres de votre famille	○	○

J2. Avez-vous vécu une des situations stressantes suivantes au cours des 12 derniers mois?

		Oui	Non
a.	Vous avez été impliqué(e) dans un accident mettant votre vie en jeu	○	○
b.	Vous avez été victime d'intimidation ou d'harcèlement	○	○
c.	Vous avez été attaqué(e) physiquement de manière sérieuse (par exemple, vol avec arme)	○	○
d.	Vous avez été agressé(e) sexuellement ou violé(e)	○	○
e.	Vous avez été victime d'harcèlement sexuel (par exemple, quelqu'un a fait des remarques sexuelles inappropriées à propos de vous ou de votre corps)	○	○
f.	Vous avez eu des problèmes avec la police	○	○
g.	Vous avez passé du temps en prison	○	○
h.	Vous avez eu un problème légal sérieux	○	○
i.	Tout autre évènement stressant *(Décrivez brièvement dans la boite de texte ci-dessous)*	○	○

J3. Quel niveau de stress avez-vous actuellement par rapport aux domaines suivants de votre vie?

		Très sévère	Sévère	Modéré	Léger	Aucun
a.	Votre situation financière	○	○	○	○	○
b.	Votre santé	○	○	○	○	○
c.	Votre performance académique					
d.	Votre vie amoureuse	○	○	○	○	○
e.	Vos relations avec votre famille	○	○	○	○	○
f.	Vos relations avec les personnes au travail ou à l'école	○	○	○	○	○
g.	La santé des personnes qui vous sont chères	○	○	○	○	○
h.	Les problèmes ou difficultés des personnes qui vous sont chères	○	○	○	○	○
i.	Votre vie en générale	○	○	○	○	○

J4. Les prochaines questions sont à propos de votre vie sociale. À quelle fréquence parlez-vous au téléphone, envoyez-vous des SMS (textos) ou chattez-vous en ligne avec vos amis ou vos proches?

- ◯ Presque tous les jours
- ◯ Quelques fois par semaines
- ◯ Quelques fois par mois
- ◯ Une fois par mois
- ◯ Moins d'une fois par mois
- ◯ Jamais

J5. À quelle fréquence vous réunissez-vous en personne avec vos amis pour des activités sociales ou de loisir?

- ◯ Presque tous les jours
- ◯ Quelques fois par semaines
- ◯ Quelques fois par mois
- ◯ Une fois par mois
- ◯ Moins d'une fois par mois
- ◯ Jamais

CKPT.J6.

1. J4 OU J5 = AU MOINS "UNE FOIS PAR MOIS," VA À J6
2. TOUS LES AUTRES VA À J7

J6. Avec environ combien d'amis et de proches téléphonez-vous, envoyez-vous des SMS (textos), chattez-vous en ligne ou réunissez-vous en personne au moins une fois par mois?

_____ NOMBRE D'AMIS ET MEMBRES DE VOTRE FAMILLE

J7. À quelle fréquence assistez-vous aux réunions des groupes religieux, fraternels, sociaux ou de loisirs auxquels vous appartenez?

- ◯ Presque tous les jours
- ◯ Quelques fois par semaines
- ◯ Quelques fois par mois
- ◯ Une fois par mois
- ◯ Moins d'une fois par mois
- ◯ Jamais

J8. À quel point pouvez-vous vous fiez à votre famille et vos amis pour du soutien et de l'aide si vous avez un problème personnel sérieux?

- ◯ Beaucoup
- ◯ Quelque peu
- ◯ Un peu → VA À J10
- ◯ Pas du tout → VA À J10

[PROGRAMMEUR: PASSER MANQUANT AVEC "BEAUCOUP"]

J9. Auprès de combien de personnes pourriez-vous trouver du soutien et du réconfort si vous aviez un problème personnel sérieux?

______ NOMBRE DE PERSONNES

J10. Combien de personnes avez-vous dans votre vie personnelle auprès de qui vous vous sentiriez à l'aise de parler de vos sentiments les plus intimes sans vous sentir embarrassé(e) si vous aviez un problème sérieux *(Si aucun, entrez "0".)*

______ NOMBRE DE PERSONNES

J11. À quelle fréquence est-ce que les personnes de votre vie personnelle demandent-ils trop de vous?

- ◯ Souvent
- ◯ Parfois
- ◯ Rarement
- ◯ Jamais

J12. À quelle fréquence est-ce que les personnes de votre vie personnelle se disputent-ils avec vous ou dissent-ils des choses qui vous font vous sentir mal à votre égard?

- ◯ Souvent
- ◯ Parfois
- ◯ Rarement
- ◯ Jamais

FIN DE SECTION

SECTION K: SEXUALITÉ

K1. Quelle est votre orientation sexuelle?

- ◯ Hétérosexuelle
- ◯ Homosexuelle, gay ou lesbienne
- ◯ Bisexuelle
- ◯ Asexuelle
- ◯ Pas certain
- ◯ Autre *(Merci de préciser dans la case ci-dessous)*

____

K2. Parmi les expression suivantes, laquelle décrit le mieux votre attirance sexuelle envers les femmes?

- ◯ Fortement attiré(e) sexuellement
- ◯ Très attiré(e) sexuellement
- ◯ Assez attiré(e) sexuellement
- ◯ Un peu attiré(e) sexuellement
- ◯ Pas du tout attiré(e) sexuellement

K3. Parmi les expression suivantes, laquelle décrit le mieux votre attirance sexuelle envers les hommes?

- ◯ Fortement attiré(e) sexuellement
- ◯ Très attiré(e) sexuellement
- ◯ Assez peu attiré(e) sexuellement
- ◯ Un peu attiré(e) sexuellement
- ◯ Pas du tout attiré(e) sexuellement

K4. Au cours des 5 dernières années, avec qui avez-vous eu une activité sexuelle? *(Nous utilisons "activité sexuelle" pour parler de n'importe quel type de contact sexuel, pas seulement un rapport sexuel génital mais aussi un rapport sexuel oral ou de masturbation.)*

- ◯ Hommes seulement
- ◯ Femmes seulement
- ◯ Non binaire uniquement
- ◯ Plus d'un de ceux énumérés
- ◯ Je n'ai pas eu d'activité sexuelle

K5. Quel est votre statut marital?

- ◯ Marié(e) ⟶ VA À CKPT.K7.
- ◯ Séparé(e)
- ◯ Divorcé(e)
- ◯ Veuf(ve)
- ◯ Jamais marié(e)

[PROGRAMMEUR: PASSER MANQUANT AVEC "JAMAIS MARIÉ"]

K6. Laquelle des affirmations suivantes décrit le mieux votre situation amoureuse ou relationnelle actuelle?

- ◯ Vivant avec quelqu'un dans une relation semblable à un mariage ou fiancé(e)
- ◯ Dans une relation stable, mais pas marié(e) ou fiancé(e)
- ◯ Avoir des relations engagées avec plusieurs personnes
- ◯ Fréquentant une ou plusieurs personnes, mais pas dans une relation stable
- ◯ Pas de fréquentation ou de relation

CKPT.K7.

1. (K4 ≠ "JE N'AI PAS EU DES RELATIONS SEXUELLES"), VA À K7
2. TOUS LES AUTRES TERMINE L'ENQUÊTE

K7. Avez-vous déjà consommé du Viagra (sildenafil), Cialis (tadalafil) ou autre médicaments similaires pour améliorer votre fonction sexuelle??

- ◯ Oui, à chaque fois que j'ai des fréquentations sexuelles
- ◯ Oui, la plupart du temps
- ◯ Oui, quelque fois
- ◯ Oui, de temps en temps
- ◯ Jamais

FIN DE L'ENQUÊTE
