# Supplementary material for: The World Mental Health International College Student Survey in Canada: Protocol for a Mental Health and Substance Use Trend Study
Source: JMIR Res Protoc. 2022 Jul 29;11(7):e35168. doi: 10.2196/35168 (PMC9377443; doi:10.2196/35168)
Supplement: Multimedia Appendix 3 [file resprot_v11i7e35168_app3.pdf]

THE UNIVERSITY OF BRITISH COLUMBIA

Faculty of Medicine
The University of British Columbia
317-2194 Health Sciences Mall
Vancouver, BC V6T 1Z3

Phone 604 822 2421
Fax 604 822 6061
www.med.ubc.ca

Multimedia Appendix 3. Study cover letter and consent form.

Welcome to the UBC Student Mental Health Survey! The following cover letter is provided to make sure that you understand this study and your rights as a participant.

Title: "An integrated online approach to mental health and substance use in university students"

Principal Investigator. Dr. Daniel Vigo, Department of Psychiatry, Faculty of Medicine at UBC.

Purpose. The purpose of the study is to understand the mental health and substance use needs of the UBC student population. The data collected will provide information and direction to the research team as they design online tools to improve mental health services available to university students. This information will also help UBC improve available services and tailor them to the needs of the student body.

Task Requirements. You will be asked to answer questions regarding your health status, with a focus on mental health, substance use, risk and protective behaviours. Additionally, you will be asked for some basic demographic information, which will only be used to describe the people who take part in the study as a group. The survey includes questions concerning COVID-19. Studying the dissemination of COVID-19 and analyzing how it impacts mental health is key to understanding the overall mental health and substance use needs of UBC students, and to developing adequate services.

As researchers, we cannot provide mental health services. However, a list of resources that you can access if you need help will be provided at the end of the survey. Also, in some situations the automated survey system may provide you with additional resource suggestions tailored to your specific needs in the form of emails. Your answers are anonymous and we will not be able to identify you and contact you directly, however the automated system may offer you additional information and even offer help accessing services if it detects specific responses that indicate severe distress and/or urgency.

Duration. It will take 15 to 30 minutes to complete this survey.

Remuneration. Survey participants will be entered into a draw for a $1,000 Amazon, VISA or UBC Bookstore gift card.

Anonymity/Confidentiality. In order to obtain data that can be generalizable to the whole student body, UBC's Planning and Institutional Research office has created random samples of students stratified along demographic and administrative variables, and invited these students to participate. Your responses to the survey will be completely anonymous, and it will not be possible for the research team or UBC to link any of your personal contact information to your

survey responses, which will be stored separately. All student data will be stored on secured servers located in Canada.

Right to Withdraw. Your participation in this study is entirely voluntary. You have the right to refuse to answer any specific question on any of the forms. You also have the right to withdraw your consent and stop taking part in this study at any time, without penalty. However, since all responses are anonymous your survey responses cannot be individually identified and removed once submitted.

Future Use of Data. Your anonymous research data may be deposited into a publicly accessible location at the time of publication. This can enhance the transparency of the research data and allows for external validation and fraud control, but it also allows others to access the data for re-analysis of this study or to do other kinds of analyses in the future beyond those you are consenting to in this study. In any case, your data will remain anonymous and it will not be possible to link data with individuals.

Important Information. If you have any questions or would like more information about this study, please contact Daniel Vigo at 604-822-8048 or by e-mail at daniel.vigo@ubc.ca. If you have any concerns or complaints about your rights as a research participant and/or your experiences while participating in this study, contact the Research Participant Complaint Line in the UBC Office of Research Ethics at 604-822-8598 or if long distance e-mail RSIL@ors.ubc.ca or call toll free 1-877-822-8598.

Ethics ID Number. H19-02538

To consent please click on the blue button below.

I have read in full and wish to proceed
