# Supplementary material for: The World Mental Health International College Student Survey in Canada: Protocol for a Mental Health and Substance Use Trend Study
Source: JMIR Res Protoc. 2022 Jul 29;11(7):e35168. doi: 10.2196/35168 (PMC9377443; doi:10.2196/35168)
Supplement: Multimedia Appendix 4 [file resprot_v11i7e35168_app4.pdf]

THE UNIVERSITY OF BRITISH COLUMBIA

Faculty of Medicine
The University of British Columbia
317-2194 Health Sciences Mall
Vancouver, BC V6T 1Z3

Phone 604 822 2421
Fax 604 822 6061
www.med.ubc.ca

Multimedia Appendix 4. Sample recruitment emails and follow-up scripts.

Invitation Email

Email subject: Please Participate in this UBC Student Mental Health Study to Help Build Better Mental Health Supports for University Students

Sent on behalf of Daniel Vigo, Principal Investigator and Ainsley Carry, Vice President, Students

Dear (name of student),

University life is an exciting time, but it can also be stressful. At UBC, we know emotional wellbeing is a key step towards academic achievement and a fulfilling life. We hope to create an environment where you can feel comfortable facing these challenges and are able to seek support. In order to achieve this, we need your help to better understand the mental health and substance use landscape of our students. This is particularly important as we face the COVID-19 pandemic, which will certainly have an impact on the emotional wellbeing of our community.

We are reaching out to request your participation in the UBC Student Mental Health Survey, to help us build a better mental health system for university students. This survey is a part of the research study “An integrated online approach to mental health and substance use in university students”. The data collected will provide information and direction to design online tools and improve the services available to you.

The survey will take 15 to 30 minutes to complete, and to thank you for your time and effort you will be entered in a draw for a ***$1,000 UBC Bookstore, Amazon, or VISA gift card.***

Click here to start the survey

You have been randomly selected to participate in this one-time survey. It is anonymous, confidential, and at no time will the surveyors or UBC be able to match identities to responses. Given the importance of obtaining valid results, we will send reminder emails and contact a subset of non-responders at a later time.

To participate in the survey, click here

Warm Regards,

Dr. Daniel Vigo
Department of Psychiatry, Faculty of Medicine at the University of British Columbia
Phone: [redacted] / Email: [redacted]

Dr. Ainsley Carry
Vice President, Students, University of British Columbia
Email: [redacted]

Follow the link to opt out of future emails:
Click here to unsubscribe

THE UNIVERSITY OF BRITISH COLUMBIA

Faculty of Medicine
The University of British Columbia
317-2194 Health Sciences Mall
Vancouver, BC V6T 1Z3

Phone 604 822 2421
Fax 604 822 6061
www.med.ubc.ca

Reminder Email

Email subject: Reminder: Please Participate in the UBC Student Mental Health Study!

Sent on behalf of Daniel Vigo, Principal Investigator and Ainsley Carry, Vice President Students

Dear (name of student),

This is a reminder to participate in the UBC Student Mental Health Survey. This one-time survey is part of the research study "An integrated online approach to mental health and substance use in university students" and will help us build a better mental health system. Please note that your survey responses were automatically saved, allowing **you to finish the survey by returning to where you left off.** To thank you for your time, you will be entered in a draw for a **$1,000 UBC Bookstore, Amazon, or VISA gift card.**

Please click here to start your survey.

(If you have already started the survey, the link above will redirect you to where you left off)

Warm Regards,

Dr. Daniel Vigo
Department of Psychiatry, Faculty of Medicine at the University of British Columbia (UBC)
Phone: [redacted]
Email: [redacted]

Dr. Ainsley Carry
Vice President, Students, University of British Columbia (UBC)
Email: [redacted]

Follow the link to opt out of future emails:
Click here to unsubscribe

THE UNIVERSITY OF BRITISH COLUMBIA

Faculty of Medicine
The University of British Columbia
317-2194 Health Sciences Mall
Vancouver, BC V6T 1Z3

Phone 604 822 2421
Fax 604 822 6061
www.med.ubc.ca

Follow-Up Email for NRa Phone Group

Email Subject: Please Participate in the UBC Student Mental Health Study and help us build a better mental health system together!

Sent on behalf of Daniel Vigo, Principal Investigator and Ainsley Carry, Vice President Students

Dear (name of student),

We recently emailed you to request your participation in the UBC Student Mental Health Survey, a part of the research study "An integrated online approach to mental health and substance use in university students". The link to that survey is closed, but we really need to get additional responses to make the results valid. You are part of a small number of students that are being provided a new survey link and a final opportunity to win a **$1,000 UBC Bookstore, Amazon, or VISA gift card.**

Please click here to complete your survey.

This one-time survey will help us build a better mental health system together. It is anonymous, confidential, and at no time will the surveyors or UBC be able to match identities to responses. The survey will take 15 to 30 minutes to complete and to thank you for your time and acknowledge your contribution to this collective effort, you will be entered in a draw for a **$1,000 UBC Bookstore, Amazon, or VISA gift card.**

Your responses are extremely important to improve the services UBC provides to students, so in case you are not monitoring your UBC email, we will also try calling you tomorrow. **If you do not want to receive the call, please click here.**

Warm Regards,

Dr. Daniel Vigo
Department of Psychiatry, Faculty of Medicine at the University of British Columbia (UBC)
Phone: [redacted] / Email: [redacted]

Dr. Ainsley Carry
Vice President, Students, University of British Columbia (UBC)
Email: [redacted]

THE UNIVERSITY OF BRITISH COLUMBIA

Faculty of Medicine
The University of British Columbia
317-2194 Health Sciences Mall
Vancouver, BC V6T 1Z3

Phone 604 822 2421
Fax 604 822 6061
www.med.ubc.ca

Follow-Up Email for NRb No Phone Group

Email Subject: We need your help to improve mental health resources for UBC students

Dear (name of student),

My name is Daniel Vigo. I am part of UBC's Faculty of Medicine, and Principal Investigator of the study "An integrated online approach to mental health and substance use in university students". Our goal is to better understand and serve the mental health needs of students like you, which is why you've probably seen several email invitations to complete the UBC Student Mental Health Survey. I wanted to personally follow up and explain why your response is so important to the future of UBC's mental health system.

We currently only have responses from students who are eager to complete surveys, which we know are not representative of the entire UBC population. In order to generate valid results, we need participation from students who did not respond to our initial emails. You are part of a select group of students I am following up with. I understand you may be busy, but we really care about your input.

If you are willing to help us by answering our questions, please click here.

If you would like additional details you can call us at 604-218-2142 or we can call you if you prefer (please respond to this email with your phone and a preferred time). In any case, thank you for your time and contribution to building better mental health resources for UBC students.

All participants will also be entered in a draw for a **$1,000 UBC Bookstore, Amazon, or VISA gift card.**

Warm Regards,

Dr. Daniel Vigo
Department of Psychiatry, Faculty of Medicine / UBC
Phone: [redacted] / Email: [redacted]

If you want to stop receiving emails about this survey click here

THE UNIVERSITY OF BRITISH COLUMBIA

Faculty of Medicine
The University of British Columbia
317-2194 Health Sciences Mall
Vancouver, BC V6T 1Z3

Phone 604 822 2421
Fax 604 822 6061
www.med.ubc.ca

Call Script

Hi (student's name)

My name is (RA's name) from UBC Faculty of Medicine. I hope you are having a good day/afternoon/etc. I'm calling because you were chosen at random to participate in a UBC student mental health survey. We really need your answers to get results that are representative of all students. We know you are very busy, so you will be entered in a draw for a $1,000 Amazon, VISA, or UBC Bookstore gift card to thank you for your time. We sent you an email with additional information and a link to the survey last night. Would you be willing to do it?

(…)

Would you like more information?

[If they want to do it and/or want more information] The UBC Student Mental Health & Substance Use Survey is part of Dr. Daniel Vigo's study "An integrated online approach to mental health and substance use in university students". Your name was selected from a student contact list provided by UBC Student Services. This is a one-time, completely anonymous online survey that takes 15 to 30 minutes to complete. The regular survey has closed, but you are one of the people chosen for follow-up. We have sent you an email earlier today with a new survey link. We really need you to participate so we can gather information about students who don't readily complete email surveys. Would you like the complete the survey via the email link?

[If they don't want to do it nor want more information] Thanks anyway. Is there a specific reason not to participate, so we can learn and improve our efforts in the future?

(Caller registers reason in an anonymous form)

[If the person answering the phone says that the student doesn't live there anymore and offers to provide the caller with the new telephone number left by the student the RA will accept and make a note of it. Then the RA will call the new number and destroy the note. If the Student picks up the phone, this script will recommence at the top.]

Thanks again! Goodbye.

THE UNIVERSITY OF BRITISH COLUMBIA

Faculty of Medicine
The University of British Columbia
317-2194 Health Sciences Mall
Vancouver, BC V6T 1Z3

Phone 604 822 2421
Fax 604 822 6061
www.med.ubc.ca

Voicemail

Hi (student's name),

This is (RA's name) from UBC Faculty of Medicine. I'm calling because you were chosen to participate in the UBC Student Mental Health Survey. We've sent you a final email with a survey link and extra information yesterday at 6pm from UBC Mental Health. Essentially, we really need your answers to get results that are representative of all UBC students, and not just those who are eager to complete online surveys. To thank you for your time you'll have a chance to win a $1,000 Amazon, VISA or UBC Bookstore gift card. If you have any questions you can reply to yesterday's 6pm email from UBC Mental Health, or call me back at this number: [redacted]. Thank you for your time, have a great day!

Text message

Hi (student's name), you were chosen to participate in the UBC Student Mental Health Survey. We sent you an email with additional info last night. Please follow the UBC survey link in that email for a chance to win a $1,000 UBC Bookstore, Amazon, or VISA gift card!
