# Supplementary material for: The World Mental Health International College Student Survey in Canada: Protocol for a Mental Health and Substance Use Trend Study
Source: JMIR Res Protoc. 2022 Jul 29;11(7):e35168. doi: 10.2196/35168 (PMC9377443; doi:10.2196/35168)
Supplement: Multimedia Appendix 5 [file resprot_v11i7e35168_app5.pdf]

THE UNIVERSITY OF BRITISH COLUMBIA

Faculty of Medicine
The University of British Columbia
317-2194 Health Sciences Mall
Vancouver, BC V6T 1Z3

Phone 604 822 2421
Fax 604 822 6061
www.med.ubc.ca

Multimedia Appendix 5. Risk mitigation protocol.

Protocols for risk mitigation and improved service access

As a standard general precaution, all participants will be provided with information and contact details for UBC Counselling Services and 24/7 mental health support that they can access as needed. Furthermore, given that participants will complete the online Qualtrics survey platform, we plan to assess the level and immediacy of risk for psychological distress and suicidal intent by examining participant responses to survey questions associated with manic episodes, psychotic experiences, impairments from any mental disorders, and increased risk of suicide.

Protocol for participants with increased likelihood of acting on a recent suicide plan in the coming year (1):

Participants acknowledging any suicide plan during the past 12 months (response to question G10 different than 0) plus reporting that acting upon such plan during the coming 12 months is "somewhat likely" or "very likely" (question G11) will be presented upon survey completion with a text box by the Qualtrics system (see below) that contains (a) information on relevant clinical resources and support services available 24/7, as well as (b) a request for the student's consent and permission to contact UBC Counselling Services on their behalf to obtain an expedited appointment (i.e. within one week) (see attached pop-up box). If the student consents and provides contact details, an email with the student information and willingness to be contacted will automatically be sent to the UBC Counselling Services point-person inbox (see email 6) and a safeguard inbox (see email 6). The safeguard inbox is accessible by only the UBC Principal Investigator and the UBC Research Coordinator, will not be monitored regularly, and will delete emails every two weeks. These participants will also be sent out an email that provides a detailed list of relevant 24/7 resources, and again requests permission to contact Counselling Services on their behalf both immediately (email 5) and 28 days later (email 7).

<u>Popup box 1</u>
Thank you for participating in this survey. As we mentioned, your answers to the questions are anonymous to protect your privacy. But we still want to help within our possibilities. Your responses indicate that you may be experiencing emotional distress. The study team wants to make sure you are receiving the help you need.

We would like to request your contact details, and your permission to reach out to UBC Counselling Services on your behalf and contact you. This would enable you to obtain an expedited appointment. To consent to this please tick the "I give permission to reach out to UBC

Counselling Services on my behalf and contact me" box, include your contact details in the blank spaces below and click "Next".

(tick box) I give permission to reach out to UBC Counselling Services on my behalf and contact me

Please provide your contact details
(text box)

In the meantime, we will send you an email with information on resources available to you.

Please know that Crisis Services Canada provides confidential, non-judgmental, free emotional support, 24 hours a day, 7 days a week, for people experiencing feelings of distress or despair. Call 1-800-SUICIDE (784-2433) to speak to with a crisis responder any time.

In addition, we encourage you to call a friend or family member, to call 911, or to visit the emergency department of a local hospital if you are feeling like you cannot keep yourself safe.

COVID-19-related mental health needs
If COVID-19 has affected your mental health or if responding to COVID-19 related questions has added to your stress, please consider the resources available for UBC students in the following site (some of which are also highlighted in the list below):

Your mental health during the COVID-19 Outbreak: students.ubc.ca/covid19/mental-health-during-covid-19-outbreak

<u>Email 1</u>
Dear STUDENT,

Thank you for participating in the "UBC Student Mental Health Survey". Your responses indicate that you may be experiencing emotional distress. The study team wants to make sure you are receiving the help you need.

We would like to request your contact details, and your permission to reach out to UBC Counselling Services on your behalf and contact you. This would enable you to obtain an expedited appointment. To consent to this please respond to this email and provide your phone number (you don't need to respond to this email if you already consented and sent your contact details when you completed our survey on the Qualtrics system).

Please know that Crisis Services Canada provides confidential, non-judgmental, free emotional support, 24 hours a day, 7 days a week, for people experiencing feelings of distress or despair. Call 1-800-SUICIDE (784-2433) to speak with a crisis responder any time.

We encourage you to call a friend or family member, to call 911, or to visit the emergency department of a local hospital if you are feeling like you cannot keep yourself safe. Please also note the additional services listed below.

If you reply to this email, you will be contacting UBC Counselling Services, not the research team. Please do not alter the automated subject line to ensure proper reception. For questions about the survey, the draw, etc., please contact the research team at: e.mental.health@ubc.ca

Dr. Daniel Vigo
Department of Psychiatry, Faculty of Medicine at the University of British Columbia (UBC)
Phone: [redacted]
Email: [redacted]

COVID-19-related mental health needs
If COVID-19 has affected your mental health or if responding to COVID-19 related questions has added to your stress, please consider the resources available for UBC students in the following site (some of which are also highlighted in the list below):

Your mental health during the COVID-19 Outbreak: students.ubc.ca/covid19/mental-health-during-covid-19-outbreak

Crisis Centre BC
If you or someone you know is feeling hopeless or thinking about suicide, call or chat online with a crisis responder: 1-800-784-2433 (1-800-SUICIDE) 24hs; or crisiscentrechat.ca (noon to 1 am)

UBC Counselling Services
Provides assessment, wellness planning and referral to the most appropriate level of support as well as drop-in counselling. Call 604-822-3811 to book a phone or video appointment. Hours can be found at: students.ubc.ca/health/counselling-services

UBC Student Health Services, your family physician, or a local medical clinic
A medical professional should be the first step for people requiring medication. To book an initial mental health appointment with UBC Student Health Services call 604-822-7011. Hours can be found at: students.ubc.ca/health/student-health-service

S.A.F.E.R. (Suicide Attempt Follow-up, Education & Research)
Counselling services provided through the Access and Assessment Centre at Vancouver General Hospital. To access services call 604-675-3700. For more information visit www.vch.ca/Pages/Suicide-Attempt-Follow-up,-Education---Research-SAFER.aspx?res_id=474

UBC Student Assistance Program (SAP)
Provides personal counselling and life coaching, free for UBC students. Services are provided after hours and weekends, in multiple languages, online, on the app, or by phone. Call free 1-833-590-1328 in North America. Call collect 1-604-757-9734 outside North America. Log in to the Aspiria website by using [redacted] *for both the student code and password.*

Here2Talk
Provides counselling and community referral services to students registered at B.C. post-secondary institutions. Support is available 24/7 via app, phone and web. Call 604-642-5212 or visit https://here2talk/ca/home

***Vancouver Coastal Regional Distress Line**: 604-872-3311 (available 24/7)*
***First Nations and Inuit Helpline**: 1-855-242-3310 (available 24/7)*
***BC Mental Health Support Line**: 310-6789 (available 24/7: do not add 604, 778 or 250 before the number)*

Email 2
Dear Counselling Services,

As per our risk mitigation Protocol 1, a student has provided us with permission to contact Student Counselling on their behalf to obtain urgent follow-up.

This student has provided the following contact details:
[Name]
[phone number]
[Email Address]

Could the UBC Student Counselling Services designate please contact the student (within one business day) to schedule an expedited in-person appointment (within one week). Let us know if there is any issue with this timeline to make alternative arrangements.

Please email** [redacted] **to confirm communication with the student.

Thank you,

Dr. Daniel Vigo
Department of Psychiatry, Faculty of Medicine at the University of British Columbia (UBC)
Phone: [redacted]
Email: [redacted]

Email 3
Dear STUDENT,

Thank you for participating in the "UBC Student Mental Health Survey" last month. Your responses indicate that you may be experiencing emotional distress, and we wanted to send one final follow up email to make sure you are receiving the help you need.

If you have not already provided us with your contact details and your permission to reach out to UBC Student Counselling Services on your behalf and contact you, we would like to request this information. This would enable you to obtain an expedited appointment. To consent to this please respond to this email and provide your phone number (you don't need to respond to this email if you already consented and sent your contact details when you completed our survey on the Qualtrics system or by responding to the first follow up email we sent you).

Please know that Crisis Services Canada provides confidential, non-judgmental, free emotional support, 24 hours a day, 7 days a week, for people experiencing feelings of distress or despair. Call 1-800-SUICIDE (784-2433) to speak with a crisis responder any time.

We encourage you to call a friend or family member, to call 911, or to visit the emergency department of a local hospital if you are feeling like you cannot keep yourself safe. Please also note the additional services listed below.

If you reply to this email, you will be contacting UBC Counselling Services, not the research team. Please do not alter the automated subject line to ensure proper reception. For questions about the survey, the draw, etc., please contact the research team at: e.mental.health@ubc.ca

Dr. Daniel Vigo
Department of Psychiatry, Faculty of Medicine at the University of British Columbia (UBC)
Phone: [redacted]
Email: [redacted]

COVID-19-related mental health needs
If COVID-19 has affected your mental health or if responding to COVID-19 related questions has added to your stress, please consider the resources available for UBC students in the following site (some of which are also highlighted in the list below):

Your mental health during the COVID-19 Outbreak: students.ubc.ca/covid19/mental-health-during-covid-19-outbreak

Crisis Centre BC
If you or someone you know is feeling hopeless or thinking about suicide, call or chat online with a crisis responder: 1-800-784-2433 (1-800-SUICIDE) 24hs; or crisiscentrechat.ca (noon to 1 am)

UBC Counselling Services
Provides assessment, wellness planning and referral to the most appropriate level of support as well as drop-in counselling. Call 604-822-3811 to book a phone or video appointment. Hours can be found at: students.ubc.ca/health/counselling-services

UBC Student Health Services, your family physician, or a local medical clinic
A medical professional should be the first step for people requiring medication. To book an initial mental health appointment with UBC Student Health Services call 604-822-7011. Hours can be found at: students.ubc.ca/health/student-health-service

S.A.F.E.R. (Suicide Attempt Follow-up, Education & Research)
Counselling services provided through the Access and Assessment Centre at Vancouver General Hospital. To access services call 604-675-3700. For more information visit http://www.vch.ca/Pages/Suicide-Attempt-Follow-up,-Education---Research-SAFER.aspx?res_id=474

UBC Student Assistance Program (SAP)
Provides personal counselling and life coaching, free for UBC students. Services are provided after hours and weekends, in multiple languages, online, on the app, or by phone. Call free 1-833-590-1328 in North America. Call collect 1-604-757-9734 outside North America. Log in to the Aspiria website by using [redacted] *for both the student code and password.*

Here2Talk
Provides counselling and community referral services to students registered at B.C. post-secondary institutions. Support is available 24/7 via app, phone and web. Call 604-642-5212 or visit https://here2talk/ca/home

Vancouver Coastal Regional Distress Line*: 604-872-3311 (available 24/7)*
First Nations and Inuit Helpline*: 1-855-242-3310 (available 24/7)*
BC Mental Health Support Line*: 310-6789 (available 24/7: do not add 604, 778 or 250 before the number)*

Email 4
Dear STUDENT,

You are receiving this email because UBC Counselling Services was unable to reach you via telephone or voicemail to schedule an expedited appointment as per the "UBC Student Mental Health & Substance Use Survey" follow up.

Please call us at 604-822-3811 or drop in at 1040 Brock Hall, 1874 East Mall during the hours posted on our website (students.ubc.ca/health/counselling-services) to schedule an appointment.

Sincerely,

UBC Counselling Services

Follow-up protocol for indication of suicidal thoughts
After receiving an automated email with the consenting student's contact information, the designated point person from UBC Counselling Services will contact the student directly. This contact protocol will include up to 2 phone calls. If the second phone call is unanswered a voicemail will be left. For students without a voicemail set up, email 8 wil be sent to their provided email address.

If Student Counselling envisions any issue with the proposed timeline the research team will receive an email outlining the problem and will develop an alternative approach to provide an emergency support plan for the student on a case by case basis.

Protocol for participants with a history of manic episodes (2):

Students screening positive for or reporting a lifetime manic episode (Shown question E27 and/or response to question B5b = yes) will receive an automated popup box and email thanking them for their participation and noting that due to their specific present and/or past problems they may find it helpful to contact UBC Student Health Services and/or UBC Counselling Services.

Popup box 2

Thank you for participating in this survey. As we mentioned, your answers to the questions are anonymous to protect your privacy. But we still want to help within our possibilities. Your responses indicate that you may be experiencing mood-related issues. Should you feel the level of distress is difficult to manage and you don't have the support that you need, there are a range of available options. Please connect with one of the following services for mental health and substance use supports.

COVID-19-related mental health needs

If COVID-19 has affected your mental health or if responding to COVID-19 related questions has added to your stress, please consider the resources available for UBC students in the following site (some of which are also highlighted in the list below):

Your mental health during the COVID-19 Outbreak: students.ubc.ca/covid19/mental-health-during-covid-19-outbreak

Mindhealth BC

Provides online assessment and recommendations regarding the most appropriate level of support. Visit www.mindhealthbc.ca

UBC Student Health Services, your family physician, or a local medical clinic

A medical professional should be the first step for people requiring medication. To book an initial mental health appointment with UBC Student Health Services call 604-822-7011. Hours can be found at: students.ubc.ca/health/student-health-service

UBC Counselling Services

Provides assessment, wellness planning and referral to the most appropriate level of support as well as drop-in counselling. Call 604-822-3811 to book a phone or video appointment. Hours can be found at: students.ubc.ca/health/counselling-services

UBC Student Assistance Program (SAP)

Provides personal counselling and life coaching, free for UBC students. Services are provided after hours and weekends, in multiple languages, online, on the app, or by phone. Call free 1-833-590-1328 in North America. Call collect 1-604-757-9734 outside North America. Log in to the Aspiria website by using [redacted] *for both the student code and password.*

Here2Talk
Provides counselling and community referral services to students registered at B.C. post-secondary institutions. Support is available 24/7 via app, phone and web. Call 604-642-5212 or visit https://here2talk/ca/home

Crisis Centre BC
If you or someone you know is feeling hopeless or thinking about suicide, call or chat online with a crisis responder: 1-800-784-2433 (1-800-SUICIDE) 24hs; or crisiscentrechat.ca (noon to 1 am)

Vancouver Coastal Regional Distress Line*: 604-872-3311 (available 24/7)*
First Nations and Inuit Helpline*: 1-855-242-3310 (available 24/7)*
BC Mental Health Support Line*: 310-6789 (available 24/7: do not add 604, 778 or 250 before the number)*

Email 5
Dear STUDENT,

Thank you for participating in the "UBC Student Mental Health Survey". Our goal is to better understand the mental health and substance use needs of the student body. As mentioned when you completed the survey, should you ever feel that your level of distress is difficult to manage and you don't have the support that you need, there are a range of available options. Please connect with one of the following services for mental health and substance use supports.

Warm regards,

Dr. Daniel Vigo
Department of Psychiatry, Faculty of Medicine at the University of British Columbia (UBC)
Phone: [redacted]
Email: [redacted]

COVID-19-related mental health needs
If COVID-19 has affected your mental health or if responding to COVID-19 related questions has added to your stress, please consider the resources available for UBC students in the following site (some of which are also highlighted in the list below):

Your mental health during the COVID-19 Outbreak: students.ubc.ca/covid19/mental-health-during-covid-19-outbreak

Mindhealth BC
Provides online assessment and recommendations regarding the most appropriate level of support. Visit www.mindhealthbc.ca

UBC Student Health Services, your family physician, or a local medical clinic
A medical professional should be the first step for people requiring medication. To book an initial mental health appointment with UBC Student Health Services call 604-822-7011. Hours can be found at: students.ubc.ca/health/student-health-service

UBC Counselling Services
Provides assessment, wellness planning and referral to the most appropriate level of support as well as drop-in counselling. Call 604-822-3811 to book a phone or video appointment. Hours can be found at: students.ubc.ca/health/counselling-services

UBC Student Assistance Program (SAP)
Provides personal counselling and life coaching, free for UBC students. Services are provided after hours and weekends, in multiple languages, online, on the app, or by phone. Call free 1-833-590-1328 in North America. Call collect 1-604-757-9734 outside North America. Log in to the Aspiria website by using [redacted] for both the student code and password.

Here2Talk
Provides counselling and community referral services to students registered at B.C. post-secondary institutions. Support is available 24/7 via app, phone and web. Call 604-642-5212 or visit https://here2talk/ca/home

Crisis Centre BC
If you or someone you know is feeling hopeless or thinking about suicide, call or chat online with a crisis responder: 1-800-784-2433 (1-800-SUICIDE) 24hs; or crisiscentrechat.ca (noon to 1 am)

You can find out about additional services by visiting the UBC Counseling Services and UBC Student Health Services websites (https://students.ubc.ca/health/student-health-service AND students.ubc.ca/health/counselling-services). In addition, we encourage you to call a friend or family member, to call 1-800-784-2433, to call 911, or to visit the emergency department of a local hospital if you are feeling like you cannot keep yourself safe.

Vancouver Coastal Regional Distress Line*: 604-872-3311 (available 24/7)*
First Nations and Inuit Helpline*: 1-855-242-3310 (available 24/7)*
BC Mental Health Support Line*: 310-6789 (available 24/7: do not add 604, 778 or 250 before the number)*

Protocol for participants with history of psychotic experiences (3):

Students reporting a lifetime history of psychotic experiences (response to question E46 and/or question E47 = yes) will receive an automated popup box and email thanking them for their participation and noting that due to their specific present and/or past problems they may find it helpful to contact Student Health Services and/or Counselling.

Popup box 3

Thank you for participating in this survey. As we mentioned, your answers to the questions are anonymous to protect your privacy. But we still want to help within our possibilities. Your responses indicate that you have a history of troubling thoughts and/or experiences. Should you feel the level of distress is difficult to manage and you don't have the support that you need, there are a range of available options. Please connect with one of the following services for mental health and substance use supports.

COVID-19-related mental health needs

If COVID-19 has affected your mental health or if responding to COVID-19 related questions has added to your stress, please consider the resources available for UBC students in the following site (some of which are also highlighted in the list below):

Your mental health during the COVID-19 Outbreak: students.ubc.ca/covid19/mental-health-during-covid-19-outbreak

Mindhealth BC

Provides online assessment and recommendations regarding the most appropriate level of support. Visit www.mindhealthbc.ca

UBC Student Health Services, your family physician, or a local medical clinic

A medical professional should be the first step for people requiring medication. To book an initial mental health appointment with UBC Student Health Services call 604-822-7011. Hours can be found at: students.ubc.ca/health/student-health-service

UBC Counselling Services

Provides assessment, wellness planning and referral to the most appropriate level of support as well as drop-in counselling. Call 604-822-3811 to book a phone or video appointment. Hours can be found at: students.ubc.ca/health/counselling-services

UBC Student Assistance Program (SAP)

Provides personal counselling and life coaching, free for UBC students. Services are provided after hours and weekends, in multiple languages, online, on the app, or by phone. Call free 1-833-590-1328 in North America. Call collect 1-604-757-9734 outside North America. Log in to the Aspiria website by using [redacted] *for both the student code and password.*

Here2Talk
Provides counselling and community referral services to students registered at B.C. post-secondary institutions. Support is available 24/7 via app, phone and web. Call 604-642-5212 or visit https://here2talk/ca/home

Crisis Centre BC
If you or someone you know is feeling hopeless or thinking about suicide, call or chat online with a crisis responder: 1-800-784-2433 (1-800-SUICIDE) 24hs; or crisiscentrechat.ca (noon to 1 am)

Vancouver Coastal Regional Distress Line*: 604-872-3311 (available 24/7)*
First Nations and Inuit Helpline*: 1-855-242-3310 (available 24/7)*
BC Mental Health Support Line*: 310-6789 (available 24/7: do not add 604, 778 or 250 before the number)*

Email 6
Dear STUDENT,

Thank you for participating in the "UBC Student Mental Health Survey". Our goal is to better understand the mental health and substance use needs of the student body. As mentioned when you completed the survey, should you ever feel that your level of distress is difficult to manage and you don't have the support that you need, there are a range of available options. Please connect with one of the following services for mental health and substance use supports.

Warm regards,

Dr. Daniel Vigo
Department of Psychiatry, Faculty of Medicine at the University of British Columbia (UBC)
Phone: [redacted]
Email: [redacted]

COVID-19-related mental health needs
If COVID-19 has affected your mental health or if responding to COVID-19 related questions has added to your stress, please consider the resources available for UBC students in the following site (some of which are also highlighted in the list below):

Your mental health during the COVID-19 Outbreak: students.ubc.ca/covid19/mental-health-during-covid-19-outbreak

Mindhealth BC
Provides online assessment and recommendations regarding the most appropriate level of support. Visit www.mindhealthbc.ca

UBC Student Health Services, your family physician, or a local medical clinic
A medical professional should be the first step for people requiring medication. To book an initial mental health appointment with UBC Student Health Services call 604-822-7011. Hours can be found at: students.ubc.ca/health/student-health-service

UBC Counselling Services
Provides assessment, wellness planning and referral to the most appropriate level of support as well as drop-in counselling. Call 604-822-3811 to book a phone or video appointment. Hours can be found at: students.ubc.ca/health/counselling-services

UBC Student Assistance Program (SAP)
Provides personal counselling and life coaching, free for UBC students. Services are provided after hours and weekends, in multiple languages, online, on the app, or by phone. Call free 1-833-590-1328 in North America. Call collect 1-604-757-9734 outside North America. Log in to the Aspiria website by using [redacted] for both the student code and password.

Here2Talk
Provides counselling and community referral services to students registered at B.C. post-secondary institutions. Support is available 24/7 via app, phone and web. Call 604-642-5212 or visit https://here2talk/ca/home

Crisis Centre BC
If you or someone you know is feeling hopeless or thinking about suicide, call or chat online with a crisis responder: 1-800-784-2433 (1-800-SUICIDE) 24hs; or crisiscentrechat.ca (noon to 1 am)

You can find out about additional services by visiting the UBC Counseling Services and UBC Student Health Services websites (https://students.ubc.ca/health/student-health-service AND students.ubc.ca/health/counselling-services). In addition, we encourage you to call a friend or family member, to call 1-800-784-2433, to call 911, or to visit the emergency department of a local hospital if you are feeling like you cannot keep yourself safe.

***Vancouver Coastal Regional Distress Line**: 604-872-3311 (available 24/7)*
***First Nations and Inuit Helpline**: 1-855-242-3310 (available 24/7)*
***BC Mental Health Support Line**: 310-6789 (available 24/7: do not add 604, 778 or 250 before the number)*

Protocol for participants with history of suicide attempts, a recent suicide attempt (past year), suicide plan during the past 12 months, or suicidal ideation during the past 30 days (4):

Participants indicating any lifetime history of suicide attempts (response to question G16 = yes), any suicide plan during the past 12 months (response to question G10 different than 0), any suicide attempt during the past 12 months (response to question G19 different than 0), or any suicidal ideation during the past 30 days (response to question G6 different than “None of the time”) will be sent an automated popup box and email by the Qualtrics survey system with specific counseling and related health service options and contact information immediately upon completing the survey.

Popup box 4

Thank you for participating in this survey. As we mentioned, your answers to the questions are anonymous to protect your privacy. But we still want to help within our possibilities. Your responses indicate that you may be experiencing emotional distress. Should you feel the level of distress is difficult to manage and you don’t have the support that you need, there are a range of available options. Please connect with one of the following services for mental health and substance use support.

COVID-19-related mental health needs

If COVID-19 has affected your mental health or if responding to COVID-19 related questions has added to your stress, please consider the resources available for UBC students in the following site (some of which are also highlighted in the list below):

Your mental health during the COVID-19 Outbreak: students.ubc.ca/covid19/mental-health-during-covid-19-outbreak

Mindhealth BC

Provides online assessment and recommendations regarding the most appropriate level of support. Visit www.mindhealthbc.ca

UBC Counselling Services

Provides assessment, wellness planning and referral to the most appropriate level of support as well as drop-in counselling. Call 604-822-3811 to book a phone or video appointment. Hours can be found at: students.ubc.ca/health/counselling-services

UBC Student Health Services, your family physician, or a local medical clinic

A medical professional should be the first step for people requiring medication. To book an initial mental health appointment with UBC Student Health Services call 604-822-7011. Hours can be found at: students.ubc.ca/health/student-health-service

S.A.F.E.R. (Suicide Attempt Follow-up, Education & Research)
Counselling services provided through the Access and Assessment Centre at Vancouver General Hospital. To access services call 604-675-3700. For more information visit www.vch.ca/Pages/Suicide-Attempt-Follow-up,-Education---Research-SAFER.aspx?res_id=474

UBC Student Assistance Program (SAP)
Provides personal counselling and life coaching, free for UBC students. Services are provided after hours and weekends, in multiple languages, online, on the app, or by phone. Call free 1-833-590-1328 in North America. Call collect 1-604-757-9734 outside North America. Log in to the Aspiria website by using [redacted] for both the student code and password.

Here2Talk
Provides counselling and community referral services to students registered at B.C. post-secondary institutions. Support is available 24/7 via app, phone and web. Call 604-642-5212 or visit https://here2talk/ca/home

Crisis Centre BC
If you or someone you know is feeling hopeless or thinking about suicide, call or chat online with a crisis responder: 1-800-784-2433 (1-800-SUICIDE) 24hs; or crisiscentrechat.ca (noon to 1 am)

***Vancouver Coastal Regional Distress Line**: 604-872-3311 (available 24/7)*
***First Nations and Inuit Helpline**: 1-855-242-3310 (available 24/7)*
***BC Mental Health Support Line**: 310-6789 (available 24/7: do not add 604, 778 or 250 before the number)*

Email 7
Dear STUDENT,

Thank you for participating in the "UBC Student Mental Health Survey". Our goal is to better understand the mental health and substance use needs of the student body. As mentioned when you completed the survey, should you ever feel that your level of distress is difficult to manage and you don't have the support that you need, there are a range of available options. Please connect with one of the following services for mental health and substance use supports.

Warm regards,

Dr. Daniel Vigo
Department of Psychiatry, Faculty of Medicine at the University of British Columbia (UBC)

Phone: [redacted]
Email: [redacted]

COVID-19-related mental health needs
If COVID-19 has affected your mental health or if responding to COVID-19 related questions has added to your stress, please consider the resources available for UBC students in the following site (some of which are also highlighted in the list below):

Your mental health during the COVID-19 Outbreak: students.ubc.ca/covid19/mental-health-during-covid-19-outbreak

Mindhealth BC
Provides online assessment and recommendations regarding the most appropriate level of support. Visit www.mindhealthbc.ca

UBC Counselling Services
Provides assessment, wellness planning and referral to the most appropriate level of support as well as drop-in counselling. Call 604-822-3811 to book a phone or video appointment. Hours can be found at: students.ubc.ca/health/counselling-services

UBC Student Health Services, your family physician, or a local medical clinic
A medical professional should be the first step for people requiring medication. To book an initial mental health appointment with UBC Student Health Services call 604-822-7011. Hours can be found at: students.ubc.ca/health/student-health-service

S.A.F.E.R. (Suicide Attempt Follow-up, Education & Research)
Counselling services provided through the Access and Assessment Centre at Vancouver General Hospital. To access services call 604-675-3700. For more information visit www.vch.ca/Pages/Suicide-Attempt-Follow-up,-Education---Research-SAFER.aspx?res_id=474

UBC Student Assistance Program (SAP)
Provides personal counselling and life coaching, free for UBC students. Services are provided after hours and weekends, in multiple languages, online, on the app, or by phone. Call free 1-833-590-1328 in North America. Call collect 1-604-757-9734 outside North America. Log in to the Aspiria website by using [redacted] *for both the student code and password.*

Here2Talk
Provides counselling and community referral services to students registered at B.C. post-secondary institutions. Support is available 24/7 via app, phone and web. Call 604-642-5212 or visit https://here2talk/ca/home

Crisis Centre BC

If you or someone you know is feeling hopeless or thinking about suicide, call or chat online with a crisis responder: 1-800-784-2433 (1-800-SUICIDE) 24hs; or crisiscentrechat.ca (noon to 1 am)

You can find out about additional services by visiting the UBC Counseling Services and UBC Student Health Services websites (https://students.ubc.ca/health/student-health-service AND students.ubc.ca/health/counselling-services). In addition, we encourage you to call a friend or family member, to call 1-800-784-2433, to call 911, or to visit the emergency department of a local hospital if you are feeling like you cannot keep yourself safe.

***Vancouver Coastal Regional Distress Line**: 604-872-3311 (available 24/7)*
***First Nations and Inuit Helpline**: 1-855-242-3310 (available 24/7)*
***BC Mental Health Support Line**: 310-6789 (available 24/7: do not add 604, 778 or 250 before the number)*

Protocol for participants with severe impairment resulting from any mental or substance use disorder (5):

Students screening positive for severe impairments resulting from any mental or substance use disorder (response to question B3b, B3c, B4b, or B4c = "Very severe interference" or "severe interference") will receive an automated popup box and email thanking them for their participation and noting that due to their high levels of distress they may find it helpful to contact Counselling services.

Popup box 5

Thank you for participating in this survey. As we mentioned, your answers to the questions are anonymous to protect your privacy. But we still want to help within our possibilities. Your responses indicate that you may be experiencing emotional distress. Should you feel the level of distress is difficult to manage and you don't have the support that you need, there are a range of available options. Please connect with one of the following services for mental health and substance use supports.

COVID-19-related mental health needs

If COVID-19 has affected your mental health or if responding to COVID-19 related questions has added to your stress, please consider the resources available for UBC students in the following site (some of which are also highlighted in the list below):

Your mental health during the COVID-19 Outbreak: students.ubc.ca/covid19/mental-health-during-covid-19-outbreak

Mindhealth BC

Provides online assessment and recommendations regarding the most appropriate level of support. Visit www.mindhealthbc.ca

UBC Student Health Services, your family physician, or a local medical clinic

A medical professional should be the first step for people requiring medication. To book an initial mental health appointment with UBC Student Health Services call 604-822-7011. Hours can be found at: students.ubc.ca/health/student-health-service

UBC Counselling Services

Provides assessment, wellness planning and referral to the most appropriate level of support as well as drop-in counselling. Call 604-822-3811 to book a phone or video appointment. Hours can be found at: students.ubc.ca/health/counselling-services

UBC Student Assistance Program (SAP)

Provides personal counselling and life coaching, free for UBC students. Services are provided after hours and weekends, in multiple languages, online, on the app, or by phone. Call free 1-

833-590-1328 in North America. Call collect 1-604-757-9734 outside North America. Log in to the Aspiria website by using [redacted] *for both the student code and password.*

Here2Talk
Provides counselling and community referral services to students registered at B.C. post-secondary institutions. Support is available 24/7 via app, phone and web. Call 604-642-5212 or visit https://here2talk/ca/home

Crisis Centre BC
If you or someone you know is feeling hopeless or thinking about suicide, call or chat online with a crisis responder: 1-800-784-2433 (1-800-SUICIDE) 24hs; or crisiscentrechat.ca (noon to 1 am)

Vancouver Coastal Regional Distress Line*: 604-872-3311 (available 24/7)*
First Nations and Inuit Helpline*: 1-855-242-3310 (available 24/7)*
BC Mental Health Support Line*: 310-6789 (available 24/7: do not add 604, 778 or 250 before the number)*

Email 8
Dear STUDENT,

Thank you for participating in the "UBC Student Mental Health Survey". Our goal is to better understand the mental health and substance use needs of the student body. As mentioned when you completed the survey, should you ever feel that your level of distress is difficult to manage and you don't have the support that you need, there are a range of available options. Please connect with one of the following services for mental health and substance use supports.

Warm regards,

Dr. Daniel Vigo
Department of Psychiatry, Faculty of Medicine at the University of British Columbia (UBC)
Phone: [redacted]
Email: [redacted]

COVID-19-related mental health needs
If COVID-19 has affected your mental health or if responding to COVID-19 related questions has added to your stress, please consider the resources available for UBC students in the following site (some of which are also highlighted in the list below):

Your mental health during the COVID-19 Outbreak: students.ubc.ca/covid19/mental-health-during-covid-19-outbreak

Mindhealth BC
Provides online assessment and recommendations regarding the most appropriate level of support. Visit www.mindhealthbc.ca

UBC Student Health Services, your family physician, or a local medical clinic
A medical professional should be the first step for people requiring medication. To book an initial mental health appointment with UBC Student Health Services call 604-822-7011. Hours can be found at: students.ubc.ca/health/student-health-service

UBC Counselling Services
Provides assessment, wellness planning and referral to the most appropriate level of support as well as drop-in counselling. Call 604-822-3811 to book a phone or video appointment. Hours can be found at: students.ubc.ca/health/counselling-services

UBC Student Assistance Program (SAP)
Provides personal counselling and life coaching, free for UBC students. Services are provided after hours and weekends, in multiple languages, online, on the app, or by phone. Call free 1-833-590-1328 in North America. Call collect 1-604-757-9734 outside North America. Log in to the Aspiria website by using [redacted] *for both the student code and password.*

Here2Talk
Provides counselling and community referral services to students registered at B.C. post-secondary institutions. Support is available 24/7 via app, phone and web. Call 604-642-5212 or visit https://here2talk/ca/home

Crisis Centre BC
If you or someone you know is feeling hopeless or thinking about suicide, call or chat online with a crisis responder: 1-800-784-2433 (1-800-SUICIDE) 24hs; or crisiscentrechat.ca (noon to 1 am)

You can find out about additional services by visiting the UBC Counseling Services and UBC Student Health Services websites (https://students.ubc.ca/health/student-health-service AND students.ubc.ca/health/counselling-services). In addition, we encourage you to call a friend or family member, to call 1-800-784-2433, to call 911, or to visit the emergency department of a local hospital if you are feeling like you cannot keep yourself safe.

Vancouver Coastal Regional Distress Line*: 604-872-3311 (available 24/7)*
First Nations and Inuit Helpline*: 1-855-242-3310 (available 24/7)*
BC Mental Health Support Line*: 310-6789 (available 24/7: do not add 604, 778 or 250 before the number)*

Standard General Protocol (6):

Popup box 6- All participants who do not trigger a specific protocol will be shown a box containing the following message:

Please take the opportunity to save this information by cutting and pasting it to an editable file of your choosing, printing the screen, or clicking here to receive it in email format. You or a friend of yours may find it useful in the future.

Thank you for participating in this survey. University life is an exciting time, but it can also be stressful, and it is normal to feel distress and other negative emotions. Should you feel the level of distress is difficult to manage and you don't have the support that you need, there are a range of available options. Please connect with one of the following services for mental health and substance use support.

COVID-19-related mental health needs
If COVID-19 has affected your mental health or if responding to COVID-19 related questions has added to your stress, please consider the resources available for UBC students in the following site (some of which are also highlighted in the list below):

Your mental health during the COVID-19 Outbreak: students.ubc.ca/covid19/mental-health-during-covid-19-outbreak

Mindhealth BC
Provides online assessment and recommendations regarding the most appropriate level of support. Visit www.mindhealthbc.ca

UBC Student Assistance Program (SAP)
Provides personal counselling and life coaching, free for UBC students. Services are provided after hours and weekends, in multiple languages, online, on the app, or by phone. Call free 1-833-590-1328 in North America. Call collect 1-604-757-9734 outside North America. Log in to the Aspiria website by using [redacted] for both the student code and password.

Here2Talk
Provides counselling and community referral services to students registered at B.C. post-secondary institutions. Support is available 24/7 via app, phone and web. Call 604-642-5212 or visit https://here2talk/ca/home

UBC Counselling Services
Provides assessment, wellness planning and referral to the most appropriate level of support as well as drop-in counselling. Call 604-822-3811 to book a phone or video appointment. Hours can be found at: students.ubc.ca/health/counselling-services

Crisis Centre BC
If you or someone you know is feeling hopeless or thinking about suicide, call or chat online with a crisis responder: 1-800-784-2433 (1-800-SUICIDE) 24hs; or crisiscentrechat.ca (noon to 1 am)

Vancouver Coastal Regional Distress Line*: 604-872-3311 (available 24/7)*
First Nations and Inuit Helpline*: 1-855-242-3310 (available 24/7)*
BC Mental Health Support Line*: 310-6789 (available 24/7: do not add 604, 778 or 250 before the number)*
